# Evaluation of the pollution pressures posed by groups of chemicals on British riverine invertebrate populations

**DOI:** 10.1111/brv.70075

**Published:** 2025-09-21

**Authors:** Imogen P. Poyntz‐Wright, Xavier A. Harrison, Charles R. Tyler

**Affiliations:** ^1^ Biosciences, Geoffrey Pope Building, University of Exeter Stocker Road Exeter EX4 4QD UK; ^2^ Centre for Ecology and Conservation, University of Exeter Penryn TR10 9FE UK

**Keywords:** chemicals, rivers, invertebrates, risk assessment, toxicity

## Abstract

Globally, rivers receive a diverse range of chemicals, including metals, pesticides, persistent organic pollutants, petrochemicals, human and veterinary pharmaceuticals and personal care products. However, the extent to which these different chemical groups affect riverine invertebrate communities is not well defined. Here we set out to evaluate the available evidence for associations between British riverine invertebrate communities and different chemical groups (and individual members of these chemical groups). Our assessment comprised three elements, (*i*) an evaluation of whether environmental concentrations of these chemicals exceed the lowest effect concentrations (ECs) based on laboratory tests, (*ii*) an assessment of associations between chemical groups and changes in British riverine invertebrate communities using the existing published literature, and (*iii*) calculated potential risk of toxicity of the chemical groups to invertebrates based on measured exposures (Environmental Agency monitoring data) and laboratory‐based measurements of the lethal concentration required to kill half of the tested population (LC_50_). Our conclusions indicate that metal and pesticide pollutants (including the veterinary medicine fipronil) are of greatest concern for British riverine invertebrate communities. Petrochemicals were also of potential concern, however, risk calculations indicate this risk is lower than that for metals and pesticides. All other chemical groups assessed appeared to be of relatively low risk to British riverine invertebrates based on the available information. However, the concentrations of some pharmaceuticals and personal care products in British rivers exceeded the lowest ECs for some invertebrate species and require further investigation. Given the widespread concern regarding declines in freshwater invertebrates, studies on chemical impacts on invertebrate populations in British rivers are surprisingly limited and further targeted studies are warranted.

## I. INTRODUCTION

Freshwater environments have seen the greatest decline globally in biodiversity with species populations reductions by over 80% since 1970, compared with 38% and 36% for terrestrial and marine environments, respectively (Living Planet Report, 2016, 2020). Extinction rates for well‐studied freshwater animals are estimated to be as high as 4% per decade, which is five times greater than species losses in terrestrial systems (Living Planet Report, 2020). Factors underlying freshwater biodiversity declines are complex and include overexploitation, habitat destruction and degradation, and pollution, all of which are linked to human activities. Global regions identified with the greatest threats to freshwater diversity include the USA, Europe (excluding Scandinavia and northern Russia), the Indian subcontinent, eastern China and the Middle East (Vörösmarty *et al*., 2010; Keller *et al*., 2015; Ehalt Macedo *et al*., 2021; Jones *et al*., 2021). In Britain, riverine invertebrate populations have been severely impacted by land‐use change, invasive species, acidification, chemicals and climate change (Inverarity, Rosehill & Brooker, 1983; Aldridge, Elliott & Moggridge, 2004; Kowalik *et al*., 2007; Ormerod & Durance, 2009; Maynard & Lane, 2012; Jourdan *et al*., 2018). In recent decades, certain riverine invertebrate communities in Britain have shown signs of improvement (Vaughan & Ormerod, 2012; Outhwaite *et al*., 2020). However, some invertebrate populations, particularly molluscs, continue to decline (Vaughan & Ormerod, 2012; Outhwaite *et al*., 2020; Whelan *et al*., 2022).

Surprisingly, chemical pollution with the exception of some wastewater chemicals, has received relatively little attention regarding long‐term impacts on freshwater invertebrate populations. There is little doubt, however, that freshwater systems in Britain have been impacted significantly by water pollution associated with industrialisation and human population growth (Vaughan & Ormerod, 2012). Furthermore, even with improvements in water quality in Britain in recent decades through reductions/closure in some of the major polluting industries (e.g. coal gas production) and improvements in legislation resulting in better regulation of water pollution (e.g. Control of Pollution Act 1974, EU Urban Wastewater Treatment Directive, Environmental Permitting Regulations 2023, and voluntary schemes e.g., Agri‐environment), chemical pollution is still adversely impacting British rivers (Environment Agency, 2008b; Amisah & Cowx, 2011; Buglife, 2017; JNCC, 2021; The Rivers Trust, 2021).

Riverine biodiversity fundamentally underpins the health of freshwater systems and the ecosystem services they provide (Chakraborty, 2021) and there is a clear need to understand better to what extent chemical pollution, and which chemicals, are impacting populations of riverine invertebrates (Durance & Ormrod, 2009; Vaughan & Ormerod, 2012). Here we assess the evidence for effects of chemical groups on British riverine invertebrates through an analysis of the available literature and primary data analysis. In our approach, for different chemical groups (including pesticides, metals, petrochemicals, persistent organic pollutants, human pharmaceuticals, veterinary pharmaceuticals and human personal care products) we first determined whether their measured concentrations in British rivers exceeded the lowest effect concentrations [including lowest observed effect concentration (LOEC), lethal concentration required to kill 1%, 10%, or 50% of the population (LC_01_, LC_10_, LC_50_, respectively), effect concentration at which 10%, 20%, 25%, or 50% effect is observed (EC_10_, EC_20_, EC_25_, EC_50_, respectively)] for invertebrates based on laboratory exposures (using population‐relevant endpoints: growth, development, reproduction and mortality). To avoid possible confusion across these terms, herein the lowest effect concentrations are referred to simply as an ‘effect concentration’ (EC). Data for ECs for British invertebrates were gathered from the *ECOTOX* database. The *ECOTOX* database is the most comprehensive publicly available data source for individual chemical effects on invertebrate species. The database, as of 2021, contained data for 12,326 chemicals with over 1 million data entries (Olker *et al*., 2022). However, the database has limitations with over 6100 of the chemicals it contains having only a single entry (Olker *et al*., 2022). Data are particularly lacking for the effects of pharmaceuticals, persistent organic pollutants and veterinary chemicals on British invertebrates. We therefore conducted additional literature searches using *Web of Science* and *Google Scholar* to identify peer‐reviewed articles relating to measured concentrations of pharmaceuticals, persistent organic pollutants (POPs) and veterinary chemicals in British rivers. Search terms included the chemical name and the two most common test genera used for invertebrate testing (*Daphnia* and *Gammarus*) (see online Supporting Information, Appendix S1). We then determined the risk posed by different chemical groups through comparing the chemical concentrations recorded in English rivers (by the UK Environment Agency between 2000 and 2023) with laboratory acute ECs (LC_50_ 96 h); see Appendix S2 for further details. Together, this information was used for different chemical classes, and for individual chemicals within those classes, to assess the likelihood of an impact on populations of British riverine invertebrates over the past two decades. Chemical classes and individual chemicals that had environmental concentrations equal to or greater than the median LC_50_ 96 h for British invertebrates were deemed to have the greatest potential for harm to British invertebrates. This analysis identified the 20 chemicals of greatest concern for populations of riverine invertebrates in British rivers.

## II. CHEMICAL CONCENTRATIONS IN BRITISH RIVERS AND THE POTENTIAL FOR IMPACTS ON INVERTEBRATE POPULATIONS BASED ON LABORATORY‐DERIVED EFFECT CONCENTRATIONS

Sources of chemical pollution entering British rivers include wastewater treatment works (WwTWs) effluents, agricultural run‐off, mine tailings and urban and road run‐off. In the UK Environment Agency's 2020 assessment of rivers, 12% of England's rivers failed water quality standards (The Rivers Trust, 2021). No river assessed in England by the The Rivers Trust (2021) was designated as having surface water at ‘good’ chemical status as defined by at least one chemical exceeding its EQS (environmental quality standard; UKTAG, 2007). In 2020, there were 403,171 raw sewage spills into English rivers from storm overflows (The Rivers Trust, 2021). Clearly, chemical discharges remain a threat to British riverine invertebrate populations.

### (1) Pesticides

#### (a) River concentrations and laboratory effect concentrations

Pesticides are widely present in British rivers, with 109 pesticides detected between years 2007 and 2017 (Mohaupt, Völker & Altenburger, 2020). Other pesticides are likely present in British rivers but have not been detected due to analytical detection limits or a lack of targeted analysis. The concentrations of pesticides recorded in British rivers vary widely, from 5 × 10^−6^ to 5200 μg/l (Table S1), however, only a few pesticides have been recorded at concentrations exceeding 1000 μg/l, including tributyltin, lindane and chlorpyrifos (Table S1; Raven & George, 1989; Dowson *et al*., 1996). These concentrations, however, arose from spill events and thus are not representative of concentrations across British rivers. For some pesticides, field measurements are rather limited with a concentration derived from a single sample (and geographical location), as is the case for flusilazole and ethofumesate, and these values are unlikely to be representative of concentrations found more widely in British rivers. Nevertheless, the lowest ECs for British freshwater invertebrates have been exceeded in British rivers for 25% of pesticides for which information is currently available, including those highlighted in Table 1 (11 insecticides, one fungicide and one fungicide/molluscicide). Notably, only four of the pesticides in Table 1 (chlorpyrifos, cypermethrin, deltamethrin and imidacloprid) are amongst those used most often in arable farming and amenities in the UK (including Northern Ireland) between the years 2000 and 2020 (FERA, 2000, 2002, 2004, 2006, 2008, 2010, 2012, 2014, 2015, 2016a,b, 2018, 2020a; the use of imidacloprid and chlorpyrifos on UK crops was banned in 2018 and 2020, respectively). This underscores that pesticide usage data do not allow reliable predictions of ecological impacts on riverine invertebrates, as less‐frequently used pesticides may still pose significant risks. To illustrate this, among pesticides for which we found both UK river concentration and laboratory effect data (Table S1), dimethoate and pirimicarb had field concentrations comparable to the most commonly used pesticides between 2000 and 2020, although the lowest ECs for dimethoate and pirimicarb were significantly higher than their measured field concentrations, suggesting a lower immediate risk. Note that the *ECOTOX* database provides ECs only for a limited number of species that occur in UK freshwater rivers (Olker *et al*., 2022) and these data may not be capturing the most sensitive taxa. Among the pesticides with field concentrations exceeding ECs for riverine invertebrates, three pesticides have been consistently high and likely represent a threat to riverine invertebrate populations in Britain: diazinon, fenitrothion and malathion.

**Table 1:** Examples of pesticides from Table S1 where the lowest effect concentrations (ECs) for British freshwater invertebrates derived from laboratory‐based studies are exceeded in British rivers.

Chemical	Field concentrations (μg/l)	Number of samples	References for field concentrations	EC for growth (μg/l)	EC for development (μg/l)	EC for reproduction (μg/l)	EC for mortality (μg/l)
Chlorpyrifos	0.2–2500	27	Raven & George (1989)	3 (*Ischnura elegans* – arthropod; 6 days^1^)	**0.0075** (***Daphnia magna* – arthropod; 72 h** ^1^)	**0.021** (***Ceriodaphnia dubia* – arthropod; 7 days** ^4^)	**0.02** (***Paratya australiensis* – arthropod; 48 h** ^5^)
Cypermethrin	0.000005–0.001296	280	Environment Agency (2019a)	950 (*Alona guttata* – arthropod; 7 days^1^)		**0.000002** (***Daphnia magna* – arthropod; 21 days** ^1^)	0.002 (*Palaemonetes argentinus* – arthropod; 21 days^1^)
Deltamethrin	0.05	24	Long *et al*. (1998)	**0.005** (***Ceriodaphnia dubia* – arthropod; 8 days** ^1^)	**0.02** (***Daphnia magna* – arthropod; 21 days** ^1^)	**0.0054** (***Ceriodaphnia dubia* – arthropod; 8 days** ^4^)	**0.000006** (***Chironomus riparius* – arthropod; 28 days** ^1^)
Diazinon	0.01–0.86	24	Long *et al*. (1998)	**0.53** (***Daphnia magna* – arthropod; 21 days** ^3^)		**0.0002** (***Daphnia magna* – arthropod; 21 days** ^3^)	**0.00035** (***Daphnia magna* – arthropod; 21 days** ^2^)
	0.0061		Proctor *et al*. (2019)				
	<0.01–0.23	700	Croll (1991)				
Fenitrothion	0.01–0.22	24	Long *et al*. (1998)	**0.011** (***Daphnia magna* – arthropod; 21 days** ^1^)	3.1 (*Aedes aegypti* – arthropod; 24 h^3^)	**0.011** (***Daphnia magna* – arthropod; 21 days** ^1^)	0.23 (*Daphnia magna* – arthropod; 21 days^1^)
	0.001–0.14	9187	Comber *et al*. (2012)				
Fenvalerate	0.08–0.14	24	Long *et al*. (1998)	0.01 (*Limnephilus lunatus –* arthropod; 243.52 days^1^)	**0.096** (***Limnephilus lunatus* – arthropod; 244 days** ^1^)	**0.01** (***Cloeon dipterum* – arthropod; 29 days** ^1^)	0.0039 (*Ceriodaphnia quadrangular* – arthropod; 0.0417 days^7^)
Fipronil	0.98 (max)	2603	Spurgeon *et al*. (2021)	19.5 (*Daphnia magna* – arthropod; 21 days^1^)			**0.257** (***Hexagenia* sp. *–* arthropod; 96 h** ^2^)
Imidacloprid	0–0.13	23	Buglife (2017)	1.64 (*Chironomus riparius* – arthropod; 10 days^6^)	0.521 (*Chironomus riparius* – arthropod; 28 days^1^)	**0.07** (***Gammarus fossarum* – arthropod; 48 h** ^1^)	**0.0000109** (***Cloeon* sp. – arthropod; 96 h** ^7^)
	0.053		Proctor *et al*. (2019)				
	0.0058–0.0175	4	Casado *et al*. (2018)				
	<0.001–0.36	1325	Perkins *et al*. (2021)				
	0.0011–0.255	162	Egli *et al*. (2023)				
Lindane	0.01–0.17	24	Long *et al*. (1998)	6.11 (*Gammarus pulex* – arthropod; 14 days^1^)		**10.5** (***Ceriodaphnia dubia* – arthropod; 7 days** ^1^)	**0.8** (***Chironomus riparius* – arthropod; 10 days** ^2^)
	<0.10–0.55	700	Croll (1991)				
	<0.02–3700	180	Dowson *et al*. (1996)				
Malathion	0.01–0.11	24	Long *et al*. (1998)	**0.1** (***Daphnia magna* – arthropod; 21 days** ^1^)		**0.1** (***Daphnia magna* – arthropod; 21 days** ^1^)	0.25 (*Daphnia magna* – arthropod; 21 days^1^)
	0.001–0.109	9493	Comber *et al*. (2012)				
Parathion	0.01–0.05	24	Long *et al*. (1998)	0.85 (*Daphnia magna* – arthropod; 21 days^1^)		0.24 (*Daphnia magna* – arthropod; 21 days^3^)	**0.00031** (***Daphnia magna* – arthropod; 24 h** ^2^)
Tebuconazole	0.07	1	Environment Agency (2005)	**20** (***Attheyella crassa* – crustacean; 21 days** ^1^)	**192** (***Daphnia longispina* – arthropod; 21 days** ^1^)	**25** (***Daphnia magna* – arthropod; 21 days** ^1^)	**13.5** (***Daphnia galeata* – arthropod; 28 days** ^1^)
	0.0114–0.0141	4	Casado *et al*. (2018)				
	210	300	Spurgeon *et al*. (2021)				
Tributyltin	<0.04–5200	180	Dowson *et al*. (1996)				**72** (***Brachionus calyciflorus* – rotiferan; 24 h** ^2^)

^1^ LOEC, lowest observed effect concentration.

^2^ LC_50_, lethal concentration for 50%.

^3^ EC_50_, effect concentration for 50%.

^4^ EC_25_, effect concentration for 25%.

^5^ LC_01_, lethal concentration for 1%.

^6^ EC_10_, effect concentration for 10%.

^7^ EC_20_, effect concentration for 20%. For full list of pesticides see Table S1. Bold text is where measured field concentrations exceed ECs. All EC information was obtained from the *ECOTOX* database.

#### (b) Observed effects on invertebrates in British rivers

Various pesticide spills have been shown to impact riverine invertebrate populations. Following an accidental spill of TBT (tributyltin, an anti‐fouling agent) and lindane (an insecticide) from a timber‐processing unit, into the river Bourne, in southern England, discharge concentrations of 5200 μg/l and 3700 μg/l, respectively, resulted in the eradication of invertebrates in the immediate vicinity of the spill (Dowson *et al*., 1996). Lindane‐based pesticides are now banned from use in the UK. Similarly, invertebrate populations were eradicated following a spill event in the river Roding that resulted in chlorpyrifos concentrations reaching 2500 μg/l (Raven & George, 1989). Sustained discharges of pesticides into British rivers from surface water run‐off and/or point‐source discharges have also had impacts on invertebrate communities across eastern England from 1990 to the present day (see Poyntz‐Wright *et al*., 2023). Sustained concentrations of both acetylcholinesterase and gamma‐aminobutyric acid receptor‐acting pesticides (organophosphates, carbamates and organochlorines) have been correlated with variation in riverine invertebrate communities in the UK Midlands over several decades (Poyntz‐Wright *et al*., 2024). Such impacts of pesticides on riverine invertebrate communities have also been observed more globally (Dijk, Staalduinen & Sluijs, 2013), and pesticides appear to have long‐term sustained impacts on riverine invertebrate communities rather than impacts that arise from spills or pulse events with subsequent population recovery.

### (2) Metals

#### (a) River concentrations and laboratory effect concentrations

Around 2,276 km of British rivers are impacted by abandoned coal mines, and another 2,858 km are impacted by other abandoned‐mine types (Environment Agency, 2008a, 2019c; Beane *et al*., 2016). Surface water concentrations of dissolved (bioavailable) metals across British rivers have been reported to range from 0 to 4,380,000 μg/l, yet many metals (17 of 34 for which dissolved concentrations are available) are found at concentrations <100 μg/l, see Table S2. Only calcium, iron, magnesium, potassium, sodium and zinc have reported concentrations exceeding 10,000 μg/l and these are often related to mine waters, and thus occur across very limited subsections of British rivers (Neal *et al*., 1996; Lawlor & Tipping, 2003; Kalender, 2010; Jarvie *et al*., 2012).

More than half (57%) of the metals for which there was information in British rivers, including those listed in Table 2, exceeded the lowest ECs for freshwater invertebrates, and are potentially harmful to invertebrate populations. Silver is known to be toxic to many aquatic invertebrates and whilst field concentrations can exceed laboratory ECs (Table S2), these high levels were measured from WwTW effluent rather than general British river surface water (Gardner, Comber & Ellor, 2022). Some modelling studies have predicted silver concentrations as high as ~1.20 μg/l, but the majority are likely to be much lower at less than 0.0001 μg/l (Boxall *et al*., 2007; Dumont *et al*., 2015), well below laboratory ECs (Table S2). All the metals that have been shown to exceed the lowest ECs in British rivers (with the exception of beryllium) have done so multiple times over the past two decades. Additionally, barium, cadmium, chromium, copper, lead and zinc have been recorded at field concentrations several fold higher than lethal ECs (LC_50_, see Table 2), suggesting that they may have caused harm to British riverine invertebrate populations over prolonged periods of time.

**Table 2:** Examples taken from Table S2 of metals (dissolved) where the lowest effect concentrations (ECs) for British freshwater invertebrates derived from laboratory‐based studies are exceeded in British rivers.

Chemical	Field concentrations (μg/l)	Number of samples	References for field concentrations	Growth (μg/l)	EC for development (μg/l)	EC for reproduction (μg/l)	EC for mortality (μg/l)
Barium	1.2–16.6	150	Lawlor & Tipping (2003)				**13.5** (***Daphnia magna* – arthropod; 21 days** ^1^)
	12.7–281	484	Neal *et al*. (1996)				
	53.1	>176	Jarvie *et al*. (2000)				
	11–98	>37	Jarvie *et al*. (2012)				
	8.9–322.5	2693	Neal & Robson (2000)				
	8.9–19.55	312	Neal *et al*. (2000a)				
Beryllium	0–0.14	312	Neal *et al*. (2000a)			**5.5** (***Daphnia magna* – arthropod; 21 days** ^2^)	
	0–28.89	2693	Neal & Robson (2000)				
Cadmium	0.1–7	276	Gower *et al*. (1994)	12 (*Lymnaea stagnalis* – mollusc; 14 days^3^)		**0.15** (***Daphnia magna* – arthropod; 21 days** ^2^)	**0.0054** (***Daphnia magna* – arthropod; 48 h** ^1^)
	0.01–0.60	150	Lawlor & Tipping (2003)				
	<0.1	9	Bubb & Lester (1994)				
	0.12–0.32	15	Casper *et al*. (2004)				
	0.17	>176	Jarvie *et al*. (2000)				
	0.181–9.8		Environment Agency (2008c)				
	1–35	14	Kalender (2010)				
	0–3.47	2693	Neal & Robson (2000)				
	0.1–3.41	128	Shepherd *et al*. (2006)				
	0.05–0.18	10	Buss & Lester (1995)				
	0.01–0.1	>37	Jarvie *et al*. (2012)				
	0–3.54	312	Neal *et al*. (2000a)				
Chromium	0.0824–1.3	60	Bearcock *et al*. (2017)			**66** (***Daphnia magna* – arthropod; 21 days** ^2^)	**390** (***Austropotamobius pallipes* – arthropod; 96 h** ^1^)
	0–11.8	484	Neal *et al*. (1996)				
	0.5–1	9	Bubb & Lester (1994)				
	123–2230	32	Palumbo‐Roe *et al*. (2017)				
	0.5–204	128	Shepherd *et al*. (2006)				
	0.008–75	14	Kalender (2010)				
	0–46.8	2693	Neal & Robson (2000)				
	2.34	>176	Jarvie *et al*. (2000)				
	0–1.73	>37	Jarvie *et al*. (2012)				
	0.16–2.28	312	Neal *et al*. (2000a)				
Copper	<1–1271	276	Gower *et al*. (1994)	**3.71** (***Lymnaea stagnalis* – mollusc; 14 days** ^3^)		**9.5** (***Daphnia magna* – arthropod; 21 days** ^2^)	**0.03** (***Daphnia magna* – arthropod; 48 h** ^1^)
	0.1–3	150	Lawlor & Tipping (2003)				
	0.8–15.6	484	Neal *et al*. (1996)				
	7.95	>176	Jarvie *et al*. (2000)				
	1–5.5	9	Bubb & Lester (1994)				
	7.4–15.65	15	Casper *et al*. (2004)				
	0–97	2693	Neal & Robson (2000)				
	2.5–350	14	Kalender (2010)				
	2.07–10.35	312	Neal *et al*. (2000a)				
	1–12.3	10	Buss & Lester (1995)				
	0.79–4.34	32	Lathouri & Korre (2015)				
	1.29–70.9	128	Shepherd *et al*. (2006)				
Lead	<5–332	276	Gower *et al*. (1994)	**1.5** (***Lymnaea stagnalis* – mollusc; 14 days** ^4^)		**5.1** (***Ceriodaphnia dubia* – arthropod; 7 days** ^4^)	**28.8** (***Ceriodaphnia dubia* – arthropod; 48 h** ^1^)
	<0.05–19.4	60	Bearcock *et al*. (2017)				
	0.05–1.04	150	Lawlor & Tipping (2003)				
	1.49	>176	Jarvie *et al*. (2000)				
	0–32.5	484	Neal *et al*. (1996)				
	0.2–439.9	15	Valencia‐Avellan *et al*. (2017)				
	4.9–175		Environment Agency (2008c)				
	2–500		Mayes *et al*. (2010)				
	0–43.35	2693	Neal & Robson (2000)				
	5–285	14	Kalender (2010)				
	0.96–6.15	36	Palumbo‐Roe *et al*. (2012)				
	0.4–158	128	Shepherd *et al*. (2006)				
	0.65–1.65	10	Buss & Lester (1995)				
	0.06–4.46	312	Neal *et al*. (2000a)				
Nickel	0.1–1.2	150	Lawlor & Tipping (2003)	**115** (***Lymnaea stagnalis* – mollusc; 14 days** ^3^)		**1.3** (***Ceriodaphnia dubia* – arthropod; 10 days** ^3^)	**3.8** (***Ceriodaphnia dubia* – arthropod; 7 days** ^2^)
	0.57–21.3	484	Neal *et al*. (1996)				
	1.5–7	9	Bubb & Lester (1994)				
	3.4–400	14	Kalender (2010)				
	30.9	>176	Jarvie *et al*. (2000)				
	0.3–5.4	>37	Jarvie *et al*. (2012)				
	5–304	128	Shepherd *et al*. (2006)				
	0.36–45.37	2693	Neal & Robson (2000)				
	1.67–4.74	312	Neal *et al*. (2000a)				
	2–2660	276	Gower *et al*. (1994)	**115** (***Daphnia magna* – arthropod; 21 days** ^2^)		**14** (***Ceriodaphnia dubia* – arthropod; 7 days** ^3^)	**53** (***Ceriodaphnia dubia* – arthropod; 7 days** ^1^)
Zinc	0.2–11.8	150	Lawlor & Tipping (2003)				
	0.25–12.33	2693	Neal & Robson (2000)				
	33.6	>176	Jarvie *et al*. (2000)				
	67.8–7428.4	15	Valencia‐Avellan *et al*. (2017)				
	3.77–95.2	484	Neal *et al*. (1996)				
	4–24	9	Bubb & Lester (1994)				
	14–690	14	Kalender (2010)				
	9.14–3040		Environment Agency (2008c)				
	2.31–70.25	312	Neal *et al*. (2000a)				
	8.87–429	128	Shepherd *et al*. (2006)				

^1^ LC_50_, lethal concentration for 50%.

^2^ LOEC, lowest observed effect concentration.

^3^ EC_10_, effect concentration for 10%.

^4^ EC_20_, effect concentration for 20%. For full list of metals see Table S2. Bold text indicates where measured field concentrations exceed ECs. All EC information was obtained from the *ECOTOX* database.

#### (b) Observed effects on invertebrates in British rivers

There is little doubt that metal pollution has had a significant impact on freshwater invertebrate populations and metal pollution arguably remains one of the greatest chemical threats to riverine invertebrate populations in some regions in Britain (Johnson *et al*., 2017). What is less clear, however, is the extent to which invertebrate populations are being affected currently by metals on a national scale. At some freshwater sites historically subjected to high metal exposure from natural processes or mining selected invertebrate populations (e.g. *Gammarus pulex in the river Hayle*) appear to have adapted to these exposures through physiological mechanisms including reduced uptake and lower bioaccumulation of metals (Khan *et al*., 2011).

Field studies support associations between invertebrate diversity declines (loss of pollution‐sensitive taxa) and increasing metal pollution (Amisah & Cowx, 2011; Walker & Hassall, 2021). As one example, dissolved and sediment‐bound bioavailable metals in mining effluents in the river Afon Twymyn explained 90% of the variation in invertebrate taxa composition (Byrne, Reid & Wood, 2013). Exposure to mine waters has also been shown to result in a reduced abundance of pollution‐sensitive macroinvertebrate taxa in wetlands (Batty, Atkin & Manning, 2005). Specific metals in mining effluents responsible for these impacts include copper and iron (Hirst, Jüttner & Ormerod, 2002; Jarvis & Younger, 2006). In a study of 51 British streams, copper concentrations were shown to explain the variation (richness and abundance) of Chironomidae, Coleoptera, Leuctridae and Nemouridae (Hirst *et al*., 2002) and iron concentrations together with acidity accounted for impoverishment of invertebrates in the Stony Heap river and Helmington Row stream (Jarvis & Younger, 2006). There have been very few studies on other toxic metals such as nickel and zinc (Peters, Simpson & Moccia, 2014b; Peters *et al*., 2014a).

### (3) Petrochemicals

#### (a) River concentrations and laboratory effect concentrations

Petrochemicals are highly complex mixtures of chemicals that include polycyclic aromatic hydrocarbons (PAHs), benzene, toluene, ethylbenzene and xylene (BTEX), furans, alkenes, alkanes, cycloalkanes and aromatic hydrocarbons. Petrochemicals have well‐known toxic properties, and enter freshwater systems through spills or run‐off from road transport, industry, shipping or extraction and distribution of fossil fuels. Concentrations of petrochemicals vary widely in British rivers ranging from 0.15 to 2750 μg/l (Table S3). The majority of petrochemicals in Table S3 are found at concentrations <50 μg/l with only five petrochemicals having recorded concentrations exceeding 100 μg/l. Xylene (m & p) has been recorded in British rivers at extremely high concentrations (2750 μg/l). Four of the ten petrochemicals in Table S3 were present in British rivers at concentrations far exceeding ECs for freshwater invertebrates (Table 3) and have a high likelihood of negative impacts on British riverine invertebrates.

**Table 3:** Examples taken from Table S3 of petrochemicals where the lowest effect concentrations (ECs) for British freshwater invertebrates derived from laboratory‐based studies are exceeded in British rivers.

Chemical	Field concentrations (μg/l)	Number of samples	References for field concentrations	EC for growth (μg/l)	EC for development (μg/l)	EC for reproduction (μg/l)	EC for mortality (μg/l)
Fluoranthene	7	9006	Spurgeon *et al*. (2021)	194 (*Daphnia magna **–*** arthropod; 7 days^2^)	88 (*Chironomus riparius –* arthropod; 21.2 days^3^)	**1.5** (***Daphnia magna –* arthropod; 21 days** ^3^)	**1.2** (***Lumbriculus variegatus –* annelid; 96 h** ^1^)
bis(2‐ethylhexyl)phthalate (DEHP)	170	2355	Spurgeon *et al*. (2021)		**1** (***Chironomus riparius –* arthropod; 10 days** ^3^)	390.56 (*Daphnia magna* – arthropod; 30 days^3^)	**5** (***Daphnia magna –* arthropod; 21 days** ^1^)
Benzo[a]pyrene	1.1	548	Spurgeon *et al*. (2021)				**0.98 (*Daphnia magna –* arthropod; 48 h** ^1^)
Benz[a]anthracene	1	2049	Spurgeon *et al*. (2021)				**0.96** (***Daphnia magna –* arthropod; 48 h** ^1^)

^1^ LC_50_, lethal concentration for 50%.

^2^ EC_50_, effect concentration for 50%.

^3^ LOEC, lowest observed effect concentration. For full list of petrochemicals see Table S3. Bold text indicates where measured field concentrations exceed ECs. All EC information was obtained from the *ECOTOX* database.

For most petrochemicals for which data are available, concentrations exceeded lowest ECs in British surface waters rarely or marginally between 2007 and 2020 (Spurgeon *et al*., 2021). For example, concentrations of benz[a]anthracene and benzo[a]pyrene (Table 3) only slightly exceeded ECs for *Daphnia magna*, and therefore are unlikely to have posed a significant or sustained risk to British riverine invertebrate populations over the study period.

#### (b) Observed effects on invertebrates in British rivers

Evidence for the impacts of spills and diffuse discharges of petrochemical pollution on freshwater invertebrates in British rivers is limited. In one case a diesel spill into the river Ray, Wiltshire resulted in the loss of 90% of invertebrates to a point 50 m downstream from the spill, however, the majority of populations recovered by 13.5 months later, albeit impacts were still evident at the sites closest to the spill (Smith *et al*., 2010). Thus, high concentrations of petrochemicals can negatively affect riverine invertebrate populations. There remains a surprising lack of field observations assessing the impacts of petrochemicals on riverine invertebrates in Britain.

### (4) Human pharmaceuticals and personal care products

#### (a) River concentrations and laboratory effect concentrations

Pharmaceuticals and personal care products (PPCPs) are of increasing environmental concern due to their high levels of use and discharge into surface freshwaters *via* wastewaters (treated or untreated), leachate from landfill, agricultural run‐off and storm overflow run‐off (Ebele, Abou‐Elwafa Abdallah & Harrad, 2017). Multiple pharmaceuticals have been shown to exceed their EQS values in British surface waters and in 3–84% of tested surface water samples collected across Britain in a major chemical investigations programme (https://ukwir.org/the-chemicals-investigation-programme-phase-2,-2015-2020), 10 out of 14 monitored pharmaceuticals/metabolites exceeded ecologically safe limits (Buglife, 2021). A recent investigation across 10 English National Parks found that 13% of active pharmaceutical ingredients tested (7 out of 54 chemicals) exceeded predicted no‐effect ECs and/or lowest observed ECs for at least one location (Boxall *et al*., 2024). However, for the majority of PPCPs measured, concentrations entering British rivers are relatively low, with the majority <1 μg/l (Table S4); for 22 pharmaceuticals (active ingredients) some measured concentrations exceeded 1 μg/l (atenolol, bezafibrate, carbamazepine, cetirizine, clarithromycin, codeine, crotamiton, desvenlafaxine, diclofenac, erythromycin, fexotenadine, furosemide, gabapentin, ibuprofen, iohexol, lidocaine, lamotrigine, metformin, paracetamol, tramadol triclosan, trimethoprim). In one study, paracetamol was measured at exceptionally high concentrations of >1000 μg/l (Table S4; Ramage, Camacho‐Muñoz & Petrie, 2019). However, for many pharmaceuticals concentrations in British rivers did not exceed lowest ECs for UK freshwater invertebrates (Table S4). Most human (and some veterinary) pharmaceuticals enter rivers through WwTW effluent due to the fact they are designed not to be broken down in the body (high persistence). However, many are removed, or at least their concentrations reduced, as they pass through treatment processes, thus resulting in relatively low concentrations in rivers. The efficacy of pharmaceuticals removal through wastewater treatments does vary. For example, paracetamol, a widely used painkiller, has a reported removal rate of over 90% through activated sludge process in wastewater treatment, but some pharmaceuticals such as erythromycin and carbamazepine show consistently poor removal rates from wastewater (≤20%) (Burns *et al*., 2018). It should also be noted that the discharges from combined sewerage overflows which frequently occur into British rivers are largely uncharacterised regarding their PPCP and other chemical content.

For most pharmaceuticals their lowest ECs are several orders of magnitude higher than environmental concentrations recorded in British rivers (Table S4; 33 out of 43 chemicals have ECs of >1000 μg/l). Three pharmaceuticals appear to be exceptions, with some EC measures lower than the highest environmental concentrations measured (Table 4): paracetamol (painkiller), fluoxetine (anti‐depressant), and diclofenac (nonsteroidal anti‐inflammatory). Diclofenac measurements indicate that environmental levels have exceeded the lowest ECs for more than two decades.

**Table 4:** Examples taken from Table S4 of pharmaceuticals and personal care products where the lowest effect concentrations (ECs) for British freshwater invertebrates derived from laboratory‐based studies are exceeded in British rivers.

Chemical	Field concentrations (μg/l)	Number of samples	References for field concentrations	EC for growth (μg/l)	EC for development (μg/l)	EC for reproduction (μg/l)	EC for mortality (μg/l)
Chlorophene**	<0.003–0.016	>160	Kasprzyk‐Hordern *et al*. (2008)				**0.000126 (*Brachionus calyciflorus –* rotiferan; 24 h** ^1^)
Diclofenac	<0.0005–0.261	>160	Kasprzyk‐Hordern *et al*. (2008)			940 (*Daphnia magna* – arthropod; 21 days^2^)	**0.041 (*Dreissena polymorpha –* mollusc; 162 days** ^3^)
	0.025–2.991	125	Kay *et al*. (2017)				
	<0.002–0.568	30	Ashton *et al*. (2004)				
	0.0002–0.324	124	Niemi *et al*. (2022)				
	0.543		Zhou & Broodbank (2014)				
	0.76	2360	Spurgeon *et al*. (2021)				
	<0.0001–0.372	961	Buglife (2021)				
	0.008–0.527	351	Egli *et al*. (2023)				
	0.0882		Proctor *et al*. (2019)				
	0.0032–0.0147		Zhang & Zhou (2007)				
	<0.02–0.091	2	Hilton & Thomas (2003)				
Fluoxetine	0.0081–0.0979	60	Niemi *et al*. (2022)		13 (*Potamopyrgus antipodarum –* mollusc; 42 days^3^)	**0.02 (*Dreissena polymorpha –* mollusc; 6 days** ^3^)	330 (*Daphnia magna* – arthropod, 6 days^1^)
	<0.0001–0.0869	961	Buglife (2021)				
	0.005–0.045	124	Egli *et al*. (2023)				
	0.0012		Proctor *et al*. (2019)				
Paracetamol	0.0143–9.822	132	Burns *et al*. (2018)			**80 (*Daphnia longispina* – arthropod; 21 days** ^**2**^ **)**	6440 (*Daphnia magna* – arthropod; 48 h^3^)
	<0.0015–2.382	>160	Kasprzyk‐Hordern *et al*. (2008)				
	0.034–0.658	64	Niemi *et al*. (2022)				
	1–1100	14	Ramage *et al*. (2019)				
	0.26–1.0	24	Burns *et al*. (2017)				
	<0.05	2	Hilton & Thomas (2003)				
	0.193		Proctor *et al*. (2019)				
	0.09280–0.10849	3	Sims *et al*. (2023)				
Triclosan**	<0.0005–0.095	>160	Kasprzyk‐Hordern *et al*. (2008)	80 (*Chironomus tentans* – arthropod; 10 days^4^)		72.39 (*Daphnia magna* – arthropod; 13 days^3^)	**20 (*Chironomus tentans* – arthropod; 10 days** ^5^)
	58	874	Spurgeon *et al*. (2021)				

^1^ LC_50_, lethal concentration for 50%.

^2^ EC_50_, effect concentration for 50%.

^3^ LOEC, lowest observed effect concentration.

^4^ EC_10_, effect concentration for 10%.

^5^ LC_10_, lethal concentration for 10%. Concentrations in orange font indicate those identified through a wider literature search using *Google Scholar* and *Web of Science*; see Table S4 for specific references. All other concentration data were obtained from the *ECOTOX* database. ** = personal care products. See Table S4 for full list of pharmaceuticals and personal care products. Bold text indicates where measured field concentrations exceed ECs.

Notably, there are few data available for measured concentrations of personal care products in British rivers. However, for triclosan (an antimicrobial) and chlorophene (a biocide) they have been shown to exceed the lowest ECs for riverine invertebrates. There is little available EC information within the *ECOTOX* database for UK freshwater invertebrates for most PPCPs.

Our additional literature search was carried out to identify studies focusing on the toxic effects of PCPPs on British species of *Daphnia* and *Gammarus*. Most studies identified did not use standard toxicity EC endpoints. Of those that did (included in Table S4), these studies did not change the overall picture, with no further lowest ECs found to occur at environmentally relevant concentrations.

#### (b) Observed effects on invertebrates in British rivers

There is little direct evidence for effects of PPCPs on invertebrate populations in British rivers, however this is an understudied area. The low concentrations recorded for most PCPPs in British rivers, which in most cases do not exceed their lowest ECs, suggest that human PPCPs do not appear to be a major concern for riverine invertebrates in Britain.

### (5) Veterinary pharmaceuticals

#### (a) River concentrations and laboratory effect concentrations

Veterinary pharmaceuticals such as antibiotics, ectoparasiticides and endectocides that target parasites and other disease‐causing invertebrates are likely to pose a threat to freshwater invertebrates. Veterinary medicines can enter rivers through WwTWs (from home pet treatments, washing pet bedding, etc.), surface run‐off (largely from agriculture) and direct transmission from organisms (livestock and pets) bathing in or drinking from rivers. For veterinary medicines, few measurements of concentrations in British rivers are available, with most information based on usage rather than environmental (surface water) monitoring. From the limited data available, reported concentrations of veterinary medicines in British rivers are between 0.001 and 11,738 μg/l (Table S5). For most of these chemicals, maximum concentrations are <5 μg/l, although lincomycin (antibiotic) and propetamphos (ectoparasiticide) are much higher. Most environmental concentrations measured in British river are at least an order of magnitude lower than laboratory‐based lowest ECs, although for the pesticides fipronil, diazinon and imidacloprid (included in Table S1) river concentrations exceed the lowest laboratory‐derived ECs (see Section II.1). The limited data for veterinary medicines currently imply a low level of concern for British riverine invertebrates (Table S5). However, as for human PPCPs, there is little detailed information available on ECs for most veterinary products (in both the *ECOTOX* database and our wider literature search) and the impact of these products on riverine invertebrate populations in Britain may be underestimated.

#### (b) Observed effects on invertebrates in British rivers

There is no evidence in the literature for impacts of veterinary pharmaceuticals on British riverine invertebrate populations, with the exception of fipronil and imidacloprid (see Section II.1 and Table 1).

### (6) Persistent organic pollutants

#### (a) River concentrations and laboratory effect concentrations

POPs include a range of chemicals spanning polychlorobiphenyls (PCBs), polybrominated diphenyl ethers (PBDEs), per‐ and polyfluoroalkyl substances (PFOAs), dioxins (PCDDs), furans (PCDFs), hexabromocyclododecane (HBCDD), perfluorohexane‐1‐sulfonic acid (PFHxS) and its salts and related compounds, tetrabromodiphenyl ether and pentabromodiphenyl ether, short‐chain chlorinated paraffins (SCCPs), polychlorinated naphthalenes, decabromodiphenyl ether (commercial mixture, c‐decaBDE), hexabromodiphenyl ether and heptabromodiphenyl ether, UV‐328 (ultra‐violet absorber 328) and pesticides such as aldrin, chlordane, endrin, dieldrin, dichlorodiphenyltrichloroethane (DDT), heptachlor, mirex, hexachlorobenezene, alpha hexachlorocyclohexane, beta hexachlorocyclohexane, hexachlorobenzene (HCB), pentachlorophenol and its salts and esters, chlordecone, pentachlorobenzene and toxaphene (Environment Agency, 2015). These different classes of POP have a very wide range of uses spanning electrical, heat transfer and hydraulic equipment (PCBs), flame retardants, foams, and textiles [PBDEs and per‐ and polyfluoroalkyl substances (PFAS)]. They can be released as byproducts of manufacturing processes, and can remain in the environment for periods as long as decades and can bioaccumulate and biomagnify through the food web.

POPs occur widely in British rivers (Environment Agency, 2004, 2019b, 2021), especially shorter chain molecules such as perfluorobutane sulfonate (PFBS), PFHxS, perfluoroalkanecarboxylic acid (PFHxA), and perfluoropentanoic acid (PFPeA) (Environment Agency, 2021). Recent concerns regarding these persistent and highly mobile chemicals has led to the implementation of restrictions on PFAS use (Environment Agency, 2021) but two major classes [PFOA and perfluorooctane sulfonate (PFOS)] are still widely detected in UK rivers (Environment Agency, 2004, 2021). By contrast, concentrations of PCBs are now below biota EQS for Britain at sites across England (e.g. river Thames; Jürgens *et al*., 2015) and Scotland (Macgregor *et al*., 2010). However, note that the PCB concentrations in some biota, including for example, in eels (*Anguilla anguilla*) from the river Thames, exceed the EQS for other countries such as Canada (Jürgens *et al*., 2015). Surprisingly, given their highly toxic nature, information on British riverine surface water concentrations for POPs is scarce. Where this information is available, concentrations of POPs in riverine surface water/effluent are several orders of magnitude below the lowest ECs for laboratory‐based studies on British riverine invertebrates (Table S6) indicating a low environmental risk. However, these studies are almost exclusively limited to a maximum exposure period of 21 days, which may be insufficient given that POPs are generally highly accumulative in the natural environment. Indeed, POPs can accumulate to toxic levels, especially within sediment‐associated biota (Kean *et al*., 2021; Evenset *et al*., 2016; Windsor *et al*., 2019). Nevertheless, from a comparison of evidence from the *ECOTOX* database and wider literature and measured concentrations in British riverine waters, there is no strong evidence for POPs causing harm to riverine invertebrates.

#### (b) Observed effects on invertebrates in British rivers

We found no evidence for impacts of POPs in British rivers on invertebrate populations. As noted above, reported concentrations in British rivers have not been found to exceed lowest ECs for UK riverine invertebrates in laboratory‐based studies.

### (7) Mixtures of chemicals

As thousands of chemicals have been found in rivers, riverine invertebrates will be exposed to complex chemical mixtures. Several laboratory studies have assessed the effects of exposure to synthesised chemical mixtures on riverine invertebrates and have reported additive, synergistic or antagonistic effects (Benson & Long, 1991; Anderson & Lydy, 2002; Loureiro *et al*., 2010; LeBlanc *et al*., 2012; Mwila *et al*., 2013; Pham *et al*., 2018; Raby *et al*., 2019). In one study chlorpyrifos in combination with imidacloprid had antagonistic effects for immobilisation of *Daphnia magna* but chlorpyrifos in a mixture with nickel had synergistic effects (Loureiro *et al*., 2010). The relative ratios (concentrations) of the different chemicals in a mixture can affect the outcome. Pham *et al*. (2018) found that effects of tertiary mixtures of pesticides on *Cherax destructor* (a freshwater crustacean) could be additive or antagonistic depending on the ratio (concentrations) of pesticides in the mixture. Thus, it is likely that the effects of chemicals on riverine invertebrate communities may differ among sites depending on the chemicals involved and ratios of the different chemicals in that mixture.

## III. ANALYSIS OF THE RELATIVE TOXIC RISKS OF DIFFERENT CHEMICALS AND GROUPS OF CHEMICALS TO BRITISH RIVERINE INVERTEBRATES

We assessed the relative risks posed by the different chemicals and groups of chemicals to British riverine invertebrates by calculating Risk Quotients (see Appendix S2) for each chemical for which suitable monitoring data were available for English rivers from 2000 to 2023 (see Table S7 for list of chemicals included in the RQ analysis). To determine which chemical group/s (metal, petrochemical, pesticide, veterinary chemical, PPCP) were most likely to be toxic to riverine invertebrates, we calculated the mean of the RQs of all chemicals in that group for any given year. We then calculated the mean of these mean RQs per chemical across all years. Higher RQ values indicate a greater toxic threat posed by that chemical to British riverine invertebrates. A RQ of 1 or above represents acute lethal effects and thus the potential for a population‐level impact.

**Fig. 1:**
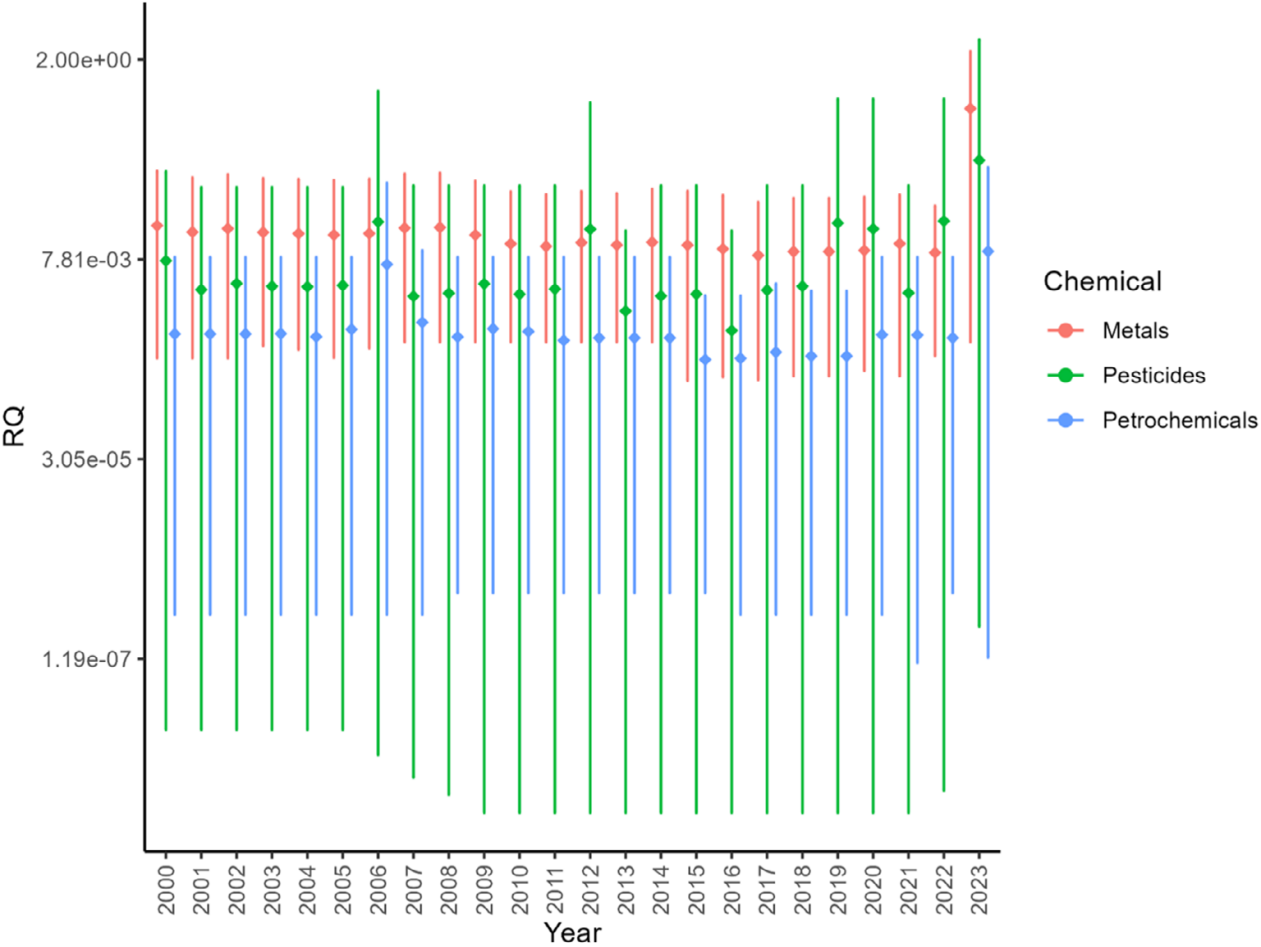
Mean Risk Quotients (RQs) and ranges for metals, pesticides, and petrochemicals in English rivers over a 24‐year period (2000–2023). Note the logarithmic scale of the *y*‐axis. The filled symbols indicate the mean RQ across all chemicals in that chemical group, and the vertical lines show the range (minimum and maximum RQ). A higher RQ represents a greater potential risk to riverine invertebrates, where the lethal concentration at which 50% of the population is affected (LC_50_) at 96 h is the same as the environmental concentration (RQ = 1). Thus, a RQ ≥1 indicates that the environmental concentration exceeds the LC_50_ at 96 h and thus acute lethal effects are likely to be present.

We found a wide range of values for the mean RQs for pesticides and petrochemicals compared with those for metals (Fig. 1). This variation among chemical groups may, in part, be explained by the number of chemicals and diversity of chemical types within each group; we had sufficient data for only five metals, compared with 50 pesticides and 14 petrochemicals (Table S7). Surprisingly, no 96‐h LC_50_ toxicity data for aquatic invertebrates inhabiting British rivers were available in the *ECOTOX* database for veterinary pharmaceuticals or human PPCPs to enable us to calculate RQs for these chemical groups.

Our analysis indicated that metals are most likely to pose the greatest risk to British freshwater invertebrates (Fig. 1) as they had the highest mean RQs across the majority of years; metals consistently exceeded a mean RQ of 0.009 (significantly higher than the mean RQ of pesticides or petrochemicals; Kruskal‐Wallis test, chi‐squared = 47, *P* = 7.9 × 10^−11^). A RQ of 0.009 suggests that British riverine invertebrate populations are not experiencing acute lethal effects for metals. However, in some years there has clearly been a greater risk to invertebrates: in 2023 the mean metal RQ was 0.51. Pesticides had the highest mean RQ in the years 2006, 2012, 2019, 2020, and 2022, and a maximum RQ of 3.48 for methoxychlor in 2023. These data suggest that pesticides also pose a threat to British riverine invertebrates, especially where acute lethal concentrations have been recorded (RQ >1).

**Fig. 2:**
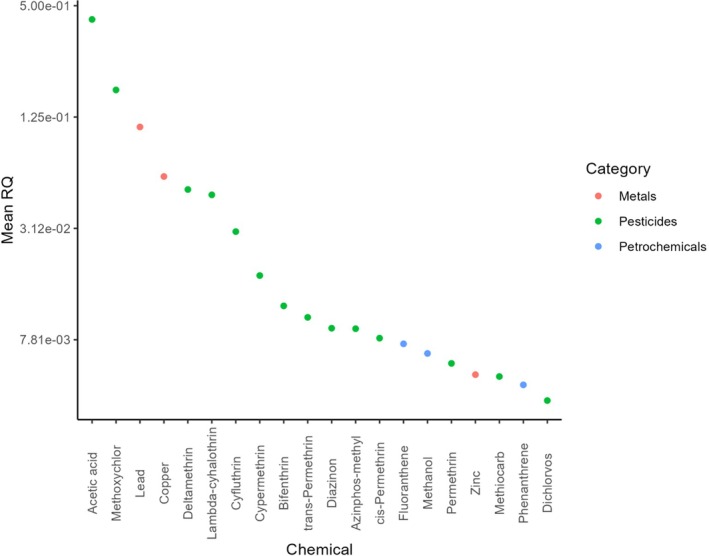
The top 20 chemicals with the highest average (mean) Risk Quotients (RQs) across the years 2000–2023. The numbers represent the average of annual RQs per chemical. Note the logarithmic scale of the *y*‐axis. See Table S8 for the number of field observations used to calculate the RQ per chemical per year.

Ranking the top 20 individual chemicals for risk to aquatic invertebrates in English rivers based on the highest mean RQs for the period between 2000 and 2023, the list includes 14 pesticides, three metals and three petrochemicals (Fig. 2). Acetic acid (herbicide) had the highest mean RQ among the chemicals measured in English rivers and across years (Fig. S1). Acetic acid is known to be more toxic to British freshwater invertebrates than some widely used herbicides such as glyphosate, but it is used in smaller quantities as a herbicide, making its use as a herbicide unlikely to represent a threat to riverine invertebrates (FERA, 2020b). However, acetic acid is also used in other industries as a food preservative, food additive and in the manufacture of ink, likely contributing significantly to the relatively high riverine concentrations recorded (GOV.UK, 2019). Notably, all other pesticides in our top 20 chemicals with the highest RQs were insecticides. Five of these (cypermethrin, deltamethrin, lambda‐cyhalothrin, bifenthrin and methiocarb) were among the most used pesticides in the UK during 2000–2020 (FERA, 2000, 2002, 2004, 2006, 2008, 2010, 2012, 2014, 2015, 2016a,b, 2018, 2020a). Whilst methiocarb, cypermethrin and bifenthrin have now been banned from use in the UK as plant protection products on all crops, deltamethrin, and lambda‐cyhalothrin are still widely authorised by the UK's Health and Safety Executive for use on crops until at least 2029 (https://secure.pesticides.gov.uk/pestreg/). Thus, the risk posed by these pesticides to riverine invertebrates is likely to remain for some time into the future. The metals lead, copper and zinc were also in our top 20 highest risk chemicals. Interestingly, copper, followed by zinc, had the highest mean RQ in most years, thus representing the greatest threat to riverine invertebrates (Fig. S2). Notably the high mean RQ for lead in Fig. 2 is distorted by an abnormally high RQ of >1 in 2023; in all years preceding this, the RQ for lead was below 0.005 (see Fig. S2). The petrochemicals fluoranthene, methanol and phenanthrene also featured in our top 20 highest risk chemicals. Fluoranthene and phenanthrene are PAHs used in numerous commercial products and are emitted as by‐products of traffic and domestic wood and coal combustion (Environment Agency, 2007b). The relatively high RQ for fluoranthene compared to other petrochemicals was consistent over the period 2000 to 2023, whereas the relatively high mean RQ for phenanthrene was the result of an abnormally high RQ in 2023 (Fig. S3). For methanol, an industrial solvent and chemical used in many products, the high mean RQ was due to an abnormally high RQ score in 2006.

Our findings for the relative risks of different chemical classes and individual chemicals to invertebrates show some differences to a previous study using RQs (Johnson *et al*., 2017). Their earlier study assessed effects on aquatic organisms more broadly (including fish), used data from non‐UK taxa, and included non‐lethal endpoints in the RQ calculation. Their top 20 chemicals of most risk to aquatic taxa included the metals copper (1st), aluminium (2nd), zinc (3rd), manganese (6th), iron (7th), lead (11th), nickel (12th), silver (15th), cadmium (16th) and arsenic (18th) and the pesticides methomyl (8th), triclosan (9th), chlorpyrifos (10th), permethrin (17th), linuron (19th) and tributyltin (20th). The only petrochemical featuring in their top 20 was benzo[a]pyrene (13th). Some of these, including aluminium and 17α‐ethinylestradiol (EE2) (4th), are known to be especially toxic to fish compared to macroinvertebrates (Gensemer & Playle, 1999; Kidd *et al*., 2007). However, both their study and ours clearly demonstrate that the chemicals posing the most risk to British riverine taxa are metals and pesticides.

## IV. CONCLUSIONS

(1) Metals and pesticides, followed by petrochemicals appear to present a risk to British riverine invertebrates.
(2) Veterinary chemicals, pharmaceuticals and personal care products, and persistent organic pollutants generally pose a low relative risk to riverine invertebrate populations. However, specific veterinary drugs like fipronil and imidacloprid, commonly used for flea treatments, are exceptions.
(3) Little information is available for many animal care treatments specifically designed to kill insect pests and this warrants further investigation as they will almost inevitably be toxic to at least some classes of freshwater invertebrates.

## AUTHOR CONTRIBUTIONS

I. P. P.‐W.: conceptualisation (co‐lead), methodology (lead), formal analysis (lead), investigation (lead), data curation (lead), resources (supporting), writing—original draft preparation (lead), writing—review and editing (equal), visualisation (lead) and project administration (lead). C. R. T.: conceptualisation (co‐lead), methodology (supporting), writing—review and editing (equal), supervision (lead), resources (lead). X. A. H.: writing—review and editing, supervision (support).

## Supporting information


**Appendix S1.** Search terms used in literature searches to identify peer‐reviewed articles for impacts of chemicals on British riverine invertebrates.
**Appendix S2**. Risk Quotient analysis – methodology.
**Table S1**. Concentrations of pesticides recorded across British rivers and lowest effect concentrations (ECs) for British freshwater invertebrates based on laboratory tests.
**Table S2**. Concentrations of metals recorded across British rivers and lowest effect concentrations (ECs) for British freshwater invertebrates based on laboratory tests.
**Table S3**. Concentrations of petrochemicals recorded across British rivers (wastewater treatment effluents) and lowest effect concentrations (ECs) for British freshwater invertebrates based on laboratory tests.
**Table S4**. Concentrations of pharmaceuticals and personal care products recorded across British rivers (wastewater treatment effluents) and lowest effect concentrations (ECs) for British freshwater invertebrates based on laboratory tests.
**Table S5**. Concentrations of veterinary pharmaceuticals recorded across British rivers (wastewater treatment effluents) and lowest effect concentrations (ECs) for British freshwater invertebrates based on laboratory tests.
**Table S6**. Concentrations of persistent organic pollutants (POPs) recorded across British rivers (wastewater treatment effluents) and lowest effect concentrations (ECs) for British freshwater invertebrates based on laboratory tests.
**Table S7**. Chemicals for which both measured environmental concentrations and lethal dose at which 50% of the population is affected (LC_50_) at 96 h toxicity information was available and thus were included in the Risk Quotient (RQ) analysis.
**Table S8**. Number of observations of riverine concentrations for each chemical collected by the Environment Agency between 2000 and 2023.
**Fig S1**. Mean Risk Quotients (RQs) of pesticides over time from 2000 to 2023.
**Fig S2**. Mean Risk Quotients (RQs) of metals over time from 2000 to 2023.
**Fig S3**. Mean Risk Quotients (RQs) of petrochemicals over time from 2000 to 2023.
