# Supplementary material for: Evaluation of the pollution pressures posed by groups of chemicals on British riverine invertebrate populations
Source: Biol Rev Camb Philos Soc. 2025 Sep 21;101(1):106–27. doi: 10.1111/brv.70075 (PMC12783454; doi:10.1111/brv.70075)
Supplement: Supplementary file 1 — Appendix S1. Search terms used in literature searches to identify peer‐reviewed articles for impacts of chemicals on British riverine invertebrates. Appendix S2. Risk Quotient analysis – methodology. Table S1. Concentrations of pesticides recorded across British rivers and lowest effect concentrations (ECs) for British freshwater invertebrates based on laboratory tests. Table S2. Concentrations of metals recorded across British rivers and lowest effect concentrations (ECs) for British freshwater invertebrates based on laboratory tests. Table S3. Concentrations of petrochemicals recorded across British rivers (wastewater treatment effluents) and lowest effect concentrations (ECs) for British freshwater invertebrates based on laboratory tests. Table S4. Concentrations of pharmaceuticals and personal care products recorded across British rivers (wastewater treatment effluents) and lowest effect concentrations (ECs) for British freshwater invertebrates based on laboratory tests. Table S5. Concentrations of veterinary pharmaceuticals recorded across British rivers (wastewater treatment effluents) and lowest effect concentrations (ECs) for British freshwater invertebrates based on laboratory tests. Table S6. Concentrations of persistent organic pollutants (POPs) recorded across British rivers (wastewater treatment effluents) and lowest effect concentrations (ECs) for British freshwater invertebrates based on laboratory tests. Table S7. Chemicals for which both measured environmental concentrations and lethal dose at which 50% of the population is affected (LC50) at 96 h toxicity information was available and thus were included in the Risk Quotient (RQ) analysis. Table S8. Number of observations of riverine concentrations for each chemical collected by the Environment Agency between 2000 and 2023. Fig S1. Mean Risk Quotients (RQs) of pesticides over time from 2000 to 2023. Fig S2. Mean Risk Quotients (RQs) of metals over time from 2000 to 2023. Fig S3. Mean Risk Quotients [file BRV-101-106-s001.docx]

Supporting Information

**Evaluation of the pollution pressures posed by groups of chemical toxicants on British riverine invertebrate populations**

Imogen Poyntz-Wright^,1*^, Xavier A. Harrison^2^, and Charles R. Tyler^1*^

1 – Biosciences, Geoffrey Pope Building, University of Exeter, Stocker Road, Exeter, EX4 4QD, UK

2 – Centre for Ecology and Conservation, University of Exeter, Penryn, TR10 9FE, UK

*Correspondence to: [ipp203@exeter.ac.uk](mailto:ipp203@exeter.ac.uk) ; [c.r.tyler@ex.ac.uk](mailto:c.r.tyler@ex.ac.uk)

**Appendix S1.** **Search terms used in literature searches to identify peer-reviewed articles for impacts of chemicals on British riverine invertebrates**

**Google Scholar queries**

("metal concentrations" OR "metal levels") AND (British OR UK OR England OR English OR Wales) AND rivers AND surface water

("pesticide concentrations" OR " pesticide levels") AND (British OR UK OR England OR English OR Wales) AND rivers AND surface water

("persistent organic pollutant concentrations" OR "persistent organic pollutant levels" OR "POP concentrations" OR "POP levels") AND (British OR UK OR England OR English OR Wales) AND rivers AND surface water

("pharmaceutical concentrations" OR " pharmaceutical levels" OR "personal care product concentrations" OR "personal care product levels") AND (British OR UK OR England OR English OR Wales) AND rivers AND surface water

("veterinary pharmaceutical concentrations" OR " veterinary pharmaceutical levels" OR " veterinary medicine concentrations" OR " veterinary medicine levels") AND (British OR UK OR England OR English OR Wales) AND rivers AND surface water

(“XXX effects on Gammarus and Daphnia: reproduction, growth, development, and mortality”) where ‘XXX’ is the chemical name

**Web of Science queries**

TS=(*pharmaceutical concentration* OR *pharmaceutical level*) AND TS=(Riverine OR "surface water") AND TS=(British OR Britain OR England OR English OR UK) (returned 23 papers)

TS=(*pesticide concentration* OR *pesticide level*) AND TS=(Riverine OR "surface water") AND TS=(British OR Britain OR England OR English OR UK) (returned 36 papers)

TS=(*metal concentration* OR *metal level*) AND TS=(Riverine OR "surface water") AND TS=(British OR Britain OR England OR English OR UK) (returned 59 papers)

TS=(*POPs concentration* OR *POP level* OR *Persistent organic pollutant concentration* OR *Persistent organic pollutant level* ) AND TS=(Riverine OR "surface water") AND TS=(British OR Britain OR England OR English OR UK) (returned 11 papers)

TS=(*Veterinary medicine concentration* OR *Veterinary medicine level* OR *Veterinary pharmaceutical concentration* OR *Veterinary pharmaceutical level*) AND TS=(Riverine OR "surface water") AND TS=(British OR Britain OR England OR English OR UK) (returned 4 papers)

TS=("XXX" AND ("toxicity" OR "effects") AND ("Gammarus" OR "Daphnia") AND ("reproduction" OR "growth" OR "development" OR "mortality")) where ‘XXX’ is the chemical name

**Appendix S2. Risk Quotient analysis – methodology**

**(1)** **Data collection**

All chemicals in the Environment Agency’s (EA) chemical monitoring database (WIMS) were classified into one of the following chemical groups: pesticides; petrochemicals; metals; pharmaceuticals and personal care products; veterinary chemicals; persistent organic pollutants. Their measured concentrations in British rivers (<https://environment.data.gov.uk/water-quality/view/landing>) were also recorded. For the chemicals monitored by the EA, laboratory toxicity information for their effects on riverine invertebrates was gathered from the *ECOTOX* database (<https://cfpub.epa.gov/ecotox/search.cfm>).

**(2) Chemical groups of potentially greatest concern for riverine British invertebrates**

We calculated risk quotients (RQs) for three groups of chemicals (pesticides, petrochemicals and metals) for the period 2000–2023. EA WIMS data on surface water chemical concentration was used to calculate the median concentration of each chemical per year (*measured environmental concentration*). The median concentration was chosen over maximum/mean to prevent the few sites at which high concentrations were reported from biasing the mean chemical concentration across all sites.

We used the *ECOTOX* database to determine the sensitivity of UK riverine invertebrate species to chemicals monitored by the UK EA. Lethal concentration required to kill 50% of the tested population (LC_50_) at 96 h was used as the endpoint to include the maximum number of toxicity studies in the analysis. The toxicity studies included considered only British invertebrate species, validated using the *NBN atlas* database. Studies with temperature and pH ranges outside British field conditions were excluded; British river temperatures have been recorded between 6 and 27 °C and pH between 6 and 8.5 (Neal & Robson, 2000; RTT, 2004; Environment Agency, 2007*a*; Deepartnership, 2022). Metal studies were only included when the dissolved (bioavailable) form was used (Johnson *et al*., 2017). All included studies reported test duration and were conducted in fresh water. As the aim was to understand the overall impact of each chemical on species of UK riverine invertebrates, all life stages were included. Using all studies that met our criteria, we calculated the median LC_50_ at 96 h for each chemical across all taxa to provide an average chemical toxicity to British riverine invertebrate species. Using data for the chemicals monitored by the EA and available laboratory toxicity studies, we calculated the risk for 14 petrochemicals, 5 metals and 50 pesticides (see Table S7) for British riverine invertebrates across a 24-year period. No exposure data were available for veterinary chemicals, and pharmaceuticals and personal care products. We calculated a risk quotient (RQ) for each chemical per year, using the following equation:

RQ = Median measured environmental concentration

Median toxicity (LC_50_ at 96 h)

To assess whether the mean RQ for each chemical group across the years 2000–2023 showed significant differences, we performed a non-parametric Kruskal–Wallis test. The residuals were non-normally distributed as determined using a Shapiro–Wilk test.

**Table S1.** Concentrations of pesticides recorded across British rivers and lowest effect concentrations (ECs) for British freshwater invertebrates based on laboratory tests. Pesticides with entries highlighted in green are included in Table 1.

| **Chemical** | **Field concentrations (µg/l)** | **Number of samples** | **References** | **EC for growth (µg/l)** | **EC for development (µg/l)** | **EC for reproduction (µg/l)** | **EC for mortality** **(µg/l)** |
| --- | --- | --- | --- | --- | --- | --- | --- |
| 2-4-D | <0.05–0.1 | 312 | Neal *et al*. (2000*b*) | 151000  (*Daphnia magna*; 21 days^1^) |  | 35100  (*Ceriodaphnia dubia*; 96 h^1^) | 3880  (*Chironomus* sp.; 48 h^2^) |
|  |  |  |  |  |  |  |  |
|  | <0.2–2.1 | 700 | Croll (1991) |  |  |  |  |
| Acetamiprid | 0–0.0005 | 23 | Buglife (2017) | 9000  (*Daphnia magna*; 21 days^1^) |  | 6200  (*Ceriodaphnia dubia*; 7 days^4^) | 20.9  (*Chironomus riparius*; 48 h^2^) |
|  | 0.006–0.192 | 346 | Egli *et al*. (2023) |  |  |  |  |
| Atrazine | <0.04–0.2 | 312 | Neal *et al*. (2000*b*) | 10000  (*Daphnia magna*; 48 h^1^) | 420  (*Daphnia magna*; 96 h^3^) | 320  (*Brachionus calyciflorus*; 7 days^1^) | 320  (*Brachionus calyciflorus*; 60.88 days^1^) |
|  | 0.01–1.60 | 24 | Long *et al*. (1998) |  |  |  |  |
|  | <0.02–7.1 | 700 | Croll (1991) |  |  |  |  |
|  | 1.7 | 813 | Spurgeon *et al*. (2021) |  |  |  |  |
|  | 0.001–0.0031 | 4 | Casado *et al*. (2018) |  |  |  |  |
|  | 0.004–0.016 | 108 | Egli *et al*. (2023) |  |  |  |  |
| Azoxystrobin | 0.76 | 2405 | Spurgeon *et al*. (2021) | 169  (*Daphnia magna*; 21 days^1^) |  | 84  (*Daphnia magna*; 21 days^1^) | 98  (*Gammarus fossarum*; 7 days^5^) |
|  | 0.0007–0.0022 | 4 | Casado *et al*. (2018) |  |  |  |  |
|  | 0.005–0.016 | 26 | Egli *et al*. (2023) |  |  |  |  |
| Bentazone | 0.06 | 1 | Environment Agency (2005) |  |  |  | 34400  (*Chironomus riparius*; 48 h^6^) |
|  | 51 | 2377 | Spurgeon *et al*. (2021) |  |  |  |  |
|  | 0.0029 | 4 | Casado *et al*. (2018) |  |  |  |  |
|  | 0.02–3.69 | 3616 | Comber *et al*. (2012) |  |  |  |  |
| Boscalid | 0.63–100 | 2816 | Spurgeon *et al*. (2021) |  |  | 2360  (*Daphnia magna*; 21 days^1^) |  |
|  | 0.0031 | 4 | Casado *et al*. (2018) |  |  |  |  |
| Bromoxynil | <0.02–0.1 | 700 | Croll (1991) | 20  (*Daphnia magna*; 28 days^1^) |  | 10  (*Daphnia magna*; 28 days^1^) | 34  (*Daphnia magna*; 28 days^2^) |
| Carbendazim | 0.0006–0.0016 | 4 | Casado *et al*. (2018) |  |  | 6.6 (*Daphnia magna*; 21 days^1^) | 3.1 (*Gammarus pulex*; 27 days^1^) |
| Carbaryl | 0.01–0.12 | 24 | Long *et al*. (1998) | 0.4  (*Daphnia magna*; 21 days^1^) | 0.4  (*Daphnia magna*; 21 days^1^) | 0.4  (*Daphnia magna*; 21 days^1^) | 1.4  (*Ceriodaphnia quadrangular*; 0.0417 days^7^) |
| Chlorpyrifos | 0.2–2500 | 27 | Raven & George (1989) | 3  (*Ischnura elegans*; 6 days^1^) | 0.0075  (*Daphnia magna*; 72 h^1^) | 0.021  (*Ceriodaphnia dubia*; 7 days^8^) | 0.02  (*Paratya australiensis*; 48 h^9^) |
| Chlortoluron | <0.1–2.6 | 700 | Croll (1991) |  |  |  |  |
|  | <0.05 | 312 | Neal *et al*. (2000*b*) |  |  |  |  |
| cis-Permethrin | 0.02–0.37 | 24 | Long *et al*. (1998) |  |  |  | 0.51  (*Ceriodaphnia dubia*; 96 h^2^) |
| Clomazone | 0.002 | 4 | Casado *et al*. (2018) | 4380 (*Daphnia magna*; 21 days^1^) |  |  |  |
| Clothianidin | 0–0.39 | 23 | Buglife (2017) | 9900  (*Daphnia magna*; 21 days^1^) |  | 370  (*Daphnia magna*; 21 days^1^) | 10.3  (*Aedes sp*; 48 h^6^) |
|  | 0.0055 | 4 | Casado *et al*. (2018) |  |  |  |  |
|  | 0.01–0.029 | 16 | Egli *et al*. (2023) |  |  |  |  |
| Cyanazine | 0.01–0.51 | 24 | Long *et al*. (1998) |  |  |  |  |
| Cypermethrin* | 0.000005–0.001296 | 280 | Environment Agency (2019*a*) | 950  (*Alona guttata*; 7 days^1^) |  | 0.000002  (*Daphnia magna*; 21 days^1^) | 0.002  (*Palaemonetes argentinus*; 21 days^1^) |
| Cyromazine | 0.022–0.038 | 5 | Egli *et al*. (2023) |  |  |  |  |
| DEET (N,N-diethyl-m-toluamide) | 19 | 11595 | Spurgeon *et al*. (2021) | 7500 (*Daphnia magna*; 21 days^1^) | 13920 (*Chironomus riparius*; 28 days^1^) | 24000 (*Daphnia magna*; 21 days^3^) | 26000 (*Daphnia magna*; 21 days^2^) |
| Deltamethrin | 0.05 | 24 | Long *et al*. (1998) | 0.005  (*Ceriodaphnia dubia*; 8 days^1^) | 0.02  (*Daphnia magna*; 21 days^1^) | 0.0054  (*Ceriodaphnia dubia*; 8 days^8^) | 0.000006  (*Chironomus riparius*; 28 days^1^) |
| Desmetryn | 0.03–0.73 | 24 | Long *et al*. (1998) |  |  |  |  |
| Diazinon | 0.01–0.86 | 24 | Long *et al*. (1998) | 0.53  (*Daphnia magna*; 21 days^3^) |  | 0.0002  (*Daphnia magna*; 21 days^3^) | 0.00035  (*Daphnia magna*; 21 days^2^) |
|  | <0.01–0.23 | 700 | Croll (1991) |  |  |  |  |
|  | 0.0061 |  | Proctor *et al*. (2019) |  |  |  |  |
| Dicamba | <0.1–0.3 | 700 | Croll (1991) |  |  |  |  |
| Dichloroprop | <0.1–0.5 | 700 | Croll (1991) |  |  |  |  |
| Dichlorvos | 0.001–0.002 | 9828 | Comber *et al*. (2012) |  |  | 10000 (*Brachionus calyciflorus*; 72 h^1^) | 0.085 (*Daphnia magna*; 48 h^2^) |
| Dimethenamid | 230 | 1091 | Spurgeon *et al*. (2021) | 2510  (*Daphnia magna*; 21 days^1^) |  |  | 2510  (*Daphnia magna*; 21 days^1^) |
|  | 0.0115–0.0144 | 4 | Casado *et al*. (2018) |  |  |  |  |
| Dimethoate | 0.01–0.54 | 24 | Long *et al*. (1998) | 100  (*Daphnia magna*; 21 days^1^) |  | 100  (*Daphnia magna*; 21 days^1^) | 3  (*Baetis rhodani*; 96 h^5^) |
|  | <0.02–0.94 | 700 | Croll (1991) |  |  |  |  |
| Diuron | 0.48 | 2522 | Spurgeon *et al*. (2021) | 3500  (*Lumbriculus variegatus*; 10 days^1^) |  | 7700  (*Daphnia pulex*; 7 days ^1^) | 3300  (*Chironomus tentans*; 21 days^1^) |
|  | <0.04–0.56 | 312 | Neal *et al*. (2000*b*) |  |  |  |  |
| Epoxiconazole | 0.34 | 1 | Environment Agency (2005) |  |  |  |  |
|  | 0.003 | 4 | Casado *et al*. (2018) |  |  |  |  |
|  | 0.2–770 | 4873 | Spurgeon *et al*. (2021) |  |  |  |  |
| Ethofumesate | 0.52 | 1 | Environment Agency (2005) | 2500  (*Daphnia magna*; 21 days^1^) |  |  | 600  (*Daphnia magna*; 21 days^1^) |
| Fenuron | 0.0025–0.0039 | 4 | Casado *et al*. (2018) | 0.036 (*Chironomus riparius*; 10 days^1^) | 2.85 (*Aedes aegypti*; 10 days^1^) |  | 0.05 (*Chironomus riparius*; 10 days^1^) |
| Fenitrothion | 0.01–0.22 | 24 | Long *et al*. (1998) | 0.011  (*Daphnia magna*; 21 days^1^) | 3.1  (*Aedes aegypti*; 24 h^3^) | 0.011  (*Daphnia magna*; 21 days^1^) | 0.23  (*Daphnia magna*; 21 days^1^) |
|  | 0.001–0.14 | 9187 | Comber *et al*. (2012) |  |  |  |  |
| Fenvalerate | 0.08 – 0.14 | 24 | Long *et al*. (1998) | 0.01  (*Limnephilus lunatus*; 243.52 days^1^) | 0.096  (*Limnephilus lunatus*; 244 days^1^) | 0.01  (*Cloeon dipterum*; 29 days^1^) | 0.0039 (*Ceriodaphnia quadrangular*; 0.0417 days^7^) |
| Fipronil | 0.98 | 2603 | Spurgeon *et al*. (2021) | 19.5  (*Daphnia magna*; 21 days^1^) |  |  | 0.257  (*Hexagenia* sp.; 96 h^2^) |
| Flusilazole | 0.07 | 1 | Environment Agency (2005) |  |  |  |  |
| Flufenacet | 1.5–98 | 2293 | Spurgeon *et al*. (2021) |  |  |  |  |
|  | 0.024 |  | Proctor *et al*. (2019) |  |  |  |  |
| Flutriafol | 0.04 | 1 | Environment Agency (2005) |  |  |  |  |
|  | 0.009–0.05 | 24 | Long *et al*. (1998) |  |  |  |  |
| Fluxapyroxad | 190 | 196 | Spurgeon *et al*. (2021) |  |  |  |  |
| Griseofulvin | 0.0012–0.0022 | 4 | Casado *et al*. (2018) |  |  |  |  |
| Glyphosate | 0.24–3.34 | 3 | Environment Agency (2005) | 1150  (*Coenagrion pulchellum*; 7 days^1^) |  | 2000  (*Brachionus calyciflorus*; 72 h^1^) | 28000  (*Brachionus calyciflorus*; 24 h^2^) |
| Imidacloprid | 0–0.13 | 23 | Buglife (2017) | 1.64  (*Chironomus riparius*; 10 days^4^) | 0.521  (*Chironomus riparius*; 28 days^1^) | 0.07  (*Gammarus fossarum*; 48 h^1^) | 0.000109  (*Cloeon* sp.; 96 h^7^) |
|  | <0.001–0.36 | 1325 | Perkins *et al*. (2021) |  |  |  |  |
|  | 0.0011–0.255 | 162 | Egli *et al*. (2023) |  |  |  |  |
|  | 0.0058–0.0175 | 4 | Casado *et al*. (2018) |  |  |  |  |
|  | 0.053 |  | Proctor *et al*. (2019) |  |  |  |  |
| Ioxynil | <0.04–0.1 | 700 | Croll (1991) |  |  |  |  |
| Isoproturon | 0.12–3.24 | 15 | Environment Agency (2005) |  |  |  |  |
|  | <0.05–11.5 | 700 | Croll (1991) |  |  |  |  |
|  | <0.04–1.63 | 312 | Neal *et al*. (2000*b*) |  |  |  |  |
| Lindane | 0.01–0.17 | 24 | Long *et al*. (1998) | 6.11  (*Gammarus pulex*; 14 days^1^) |  | 10.5  (*Ceriodaphnia dubia*; 7 days^1^) | 0.8  (*Chironomus riparius*; 10 days^2^) |
|  | <0.10–0.55 | 700 | Croll (1991) |  |  |  |  |
|  | <0.02–3700 | 180 | Dowson *et al*. (1996) |  |  |  |  |
| Linuron | 0.09 | 24 | Long *et al*. (1998) | 130  (Daphnia magna; 21 days^1^) |  | 240  (Daphnia magna; 21 days^1^) | 240  (*Daphnia magna*; 21 days^1^) |
|  | <0.2 | 700 | Croll (1991) |  |  |  |  |
| Malathion | 0.01–0.11 | 24 | Long *et al*. (1998) | 0.1  (*Daphnia magna*; 21 days^1^) |  | 0.1  (*Daphnia magna*; 21 days^1^) | 0.25  (*Daphnia magna*; 21 days^1^) |
|  | 0.001–0.109 | 9493 | Comber *et al*. (2012) |  |  |  |  |
| 4-(4-chloro-2-methylphenoxy)butyric acid (MCPB) | <0.05 | 312 | Neal *et al*. (2000*b*) |  |  |  |  |
| 2-(4-chloro-2-methylphenoxy)acetic acid (MCPA) | <0.10–16.0 | 700 | Croll (1991) |  |  |  |  |
|  | 0.12–0.1357 | 4 | Casado *et al*. (2018) |  |  |  |  |
|  | <0.05–0.1 | 312 | Neal *et al*. (2000*b*) |  |  |  |  |
| Mecoprop | <0.10–5.1 | 700 | Croll (1991) |  |  |  |  |
|  | 0.0062–0.0069 |  | Zhang & Zhou (2007) |  |  |  |  |
|  | <0.04–0.43 | 312 | Neal *et al*. (2000b) |  |  |  |  |
| Metamitron | 0.0033 | 4 | Casado *et al*. (2018) |  |  |  |  |
| Metaldehyde | 0.002–1.080 | 219 | Kay & Grayson (2014) |  |  |  |  |
|  | 0.009–4.2 | 431 | Castle *et al*. (2018) |  |  |  |  |
|  | 0.004–6.780 | 1994 | Balashova *et al*. (2021) |  |  |  |  |
|  | 0.051–0.137 | 111 | Castle *et al*. (2019) |  |  |  |  |
| Metazachlor | 0.02–0.03 | 3 | Environment Agency (2005) |  |  |  |  |
|  | 340 | 2031 | Spurgeon *et al*. (2021) |  |  |  |  |
|  | 0.0131–0.0175 | 4 | Casado *et al*. (2018) |  |  |  |  |
|  | 0.0045 |  | Proctor *et al*. (2019) |  |  |  |  |
| Metolachlor | 0.0012–0.0044 | 4 | Casado *et al*. (2018) | 707 (*Daphnia magna*; 21 days^1^) |  | 1414 (*Daphnia magna*; 21 days^1^) | 1414 (*Daphnia magna*; 21 days^1^) |
| Nicosulfuron | 0.0072 | 4 | Casado *et al*. (2018) |  |  |  |  |
| Oxadiazon | 0.0169 |  | Proctor *et al*. (2019) | 50 (*Daphnia magna*; 21 days^1^) |  |  | 110 (*Daphnia magna*; 21 days^1^) |
| Parathion | 0.01–0.05 | 24 | Long *et al*. (1998) | 0.85  (*Daphnia magna*; 21 days^1^) |  | 0.24  (*Daphnia magna*; 21 days^3^) | 0.00031  (*Daphnia magna*; 24 h^2^) |
| Pencycuron | 0.0027 | 4 | Casado *et al*. (2018) |  |  |  |  |
| Piperophos | 0.007–0.011 | 3 | Egli *et al*. (2023) |  |  |  |  |
| Pirimicarb | 0.0028 | 4 | Casado *et al*. (2018) | 100 (*Daphnia magna*; 21.25 days^1^) |  | 100 (*Daphnia magna*; 21.25 days^1^) | 19.1 (*Daphnia pulex*; 48 h^1^) |
| Pendimethalin | 0.02–0.76 | 13 | Environment Agency (2005) |  |  |  | 2300  (*Daphnia magna*; 48 h^2^) |
| Piperonyl butoxide | 0.0028 | 24 | Burns *et al*. (2017) | 210 (*Daphnia magna*; 21 days^1^) |  | 250 (*Daphnia magna*; 21 days^1^) | 120 (*Daphnia magna*; 21 days^1^) |
| Phenmedipham | 3060 | 18 | Spurgeon *et al*. (2021) |  |  |  |  |
| Prometryn | 0.03–2.44 | 24 | Long *et al*. (1998) | 2000  (*Daphnia magna*; 21 days^1^) |  |  | 12100  (*Pacifastacus leniusculus*; 96 h^2^) |
|  | 0.0028 | 4 | Casado *et al*. (2018) |  |  |  |  |
| Propanil | 0.01 | 24 | Long *et al*. (1998) | 19  (*Daphnia magna*; 21 days^1^) |  | 19  (*Daphnia magna*; 21 days^1^) | 430  (*Daphnia magna*; 21 days^1^) |
| Propamocarb | 0.005–0.018 | 21 | Egli *et al*. (2023) |  |  |  |  |
| Propazine | 0.13–3.12 | 24 | Long *et al*. (1998) | 90  (*Daphnia magna*; 21 days^1^) |  |  | 370  (*Daphnia magna*; 21 days^1^) |
|  | <0.05 | 312 | Neal *et al*. (2000*b*) |  |  |  |  |
| Propiconazole | 0.082 | 2404 | Spurgeon *et al*. (2021) |  |  | 25.6  (*Daphnia magna*; 21 days^4^) | 2.7  (*Daphnia magna*; 4 days^6^) |
| Propyzamide | 0.07–13 | 3 | Environment Agency (2005) | 1200  (*Daphnia magna*; 21 days^1^) |  |  | 1200  (*Daphnia magna*; 21 days^1^) |
|  | 2.2–72 | 5233 | Spurgeon *et al*. (2021) |  |  |  |  |
|  | 0.0029 | 4 | Casado *et al*. (2018) |  |  |  |  |
|  | <0.1–2.23 | 700 | Croll (1991) |  |  |  |  |
| Simazine | <0.04–0.17 | 312 | Neal *et al*. (2000*b*) |  |  |  | 3700  (*Cypridopsis vidua*; 48 h^2^) |
|  | 0.02–0.13 | 4 | Environment Agency (2005) |  |  |  |  |
|  | 0.01–5.42 | 24 | Long *et al*. (1998) |  |  |  |  |
|  | <0.02–7.1 | 700 | Croll (1991) |  |  |  |  |
|  | 0.006 | 3 | Egli *et al*. (2023) |  |  |  |  |
| Spiroxamine | 0.01 | 4 | Casado *et al*. (2018) |  |  |  |  |
| Tebuconazole | 0.07 | 1 | Environment Agency (2005) | 20  (*Attheyella crassa*; 21 days^1^) | 192  (*Daphnia longispina*; 21 days^1^) | 25  (*Daphnia magna*; 21 days^1^) | 13.5  (*Daphnia galeata*; 28 days^1^) |
|  | 0.0114–0.0141 | 4 | Casado *et al*. (2018) |  |  |  |  |
|  | 210 | 300 | Spurgeon *et al*. (2021) |  |  |  |  |
| Terbuthylazine (TERBA) | 118 | 96 | Spurgeon *et al*. (2021) |  |  |  |  |
|  | 0.0062–0.0141 | 4 | Casado *et al*. (2018) |  |  |  |  |
| Terbutryn | 0.35 | 1 | Environment Agency (2005) |  |  |  | 19500  (*Pacifastacus leniusculus*; 96 h^2^) |
|  | 0.01–1.76 | 24 | Long *et al*. (1998) |  |  |  |  |
|  | 9.3 | 1014 | Spurgeon *et al*. (2021) |  |  |  |  |
|  | 0.0056 | 4 | Casado *et al*. (2018) |  |  |  |  |
|  | 0.004–0.074 | 296 | Egli *et al*. (2023) |  |  |  |  |
| Thiacloprid | 0–0.0005 | 23 | Buglife (2017) | 5800  (*Daphnia magna*; 21 days^1^) |  | 1050  (*Daphnia magna*; 21 days^1^) | 0.24  (*Cloeon dipterum*; 21 days^6^) |
| Thiabendazole | 0.0005 | 4 | Casado *et al*. (2018) |  |  |  |  |
| Thiamethoxam | 0–0.06 | 23 | Buglife (2017) | 18  (*Chironomus riparius*; 10 days^1^) | 1.06  (*Cloeon dipterum*; 35 days^1^) |  | 0.81  (*Cloeon dipterum*; 28 days^6^) |
| trans-Permethrin | 0.01–0.06 | 24 | Long *et al*. (1998) |  |  |  | 0.5  (*Culex quinquefasciatus*; 24 h^2^) |
| Triallate | 0.98 | 1158 | Spurgeon *et al*. (2021) | 28  (*Daphnia magna*; 21 days^1^) |  | 56  (*Daphnia magna*; 21 days, ^1^) | 56  (*Daphnia magna*; 21 days^1^) |
| Tributyltin | <0.04–5200 | 180 | Dowson *et al*. (1996) |  |  |  | 72  (*Brachionus calyciflorus*; 24 h^2^) |
| Trifluralin | 0.012–0.111 | 6 | Environment Agency (2005) |  |  |  | 193  (*Daphnia magna*; 48 h^2^) |
|  | 0.02–6.01 | 24 | Long *et al*. (1998) |  |  |  |  |
| Triphenyltin | 0.001–0.074 | 6,089 | Comber *et al*. (2012) |  |  |  |  |

^1^LOEC, lowest observed effect concentration; ^2^LC_50_, lethal concentration for 50%; ^3^EC_50_, effect concentration for 50%) ^4^EC_10_, effect concentration for 10%; ^5^LC_20_, lethal concentration for 20% ^6^LC_10_, lethal concentration for 10%; ^7^EC_20_, effect concentration for 20%; ^8^EC_25_, effect concentration for 25%; ^9^LC_01_, lethal concentration for 1%. *Cypermethrin concentrations were taken from fresh waters (including ponds). All EC data were obtained from the *ECOTOX* database, unless otherwise specified.

**Table S2.** Concentrations of metals recorded across British rivers and lowest effect concentrations (ECs) for British freshwater invertebrates based on laboratory tests. Metals with entries highlighted in green are included in Table 2.

| **Chemical** | **Field concentrations (µg/l)** | **Number of samples** | **References** | **EC for growth (µg/l)** | **EC for development (µg/l)** | **EC for reproduction (µg/l)** | **EC for mortality (µg/l)** |
| --- | --- | --- | --- | --- | --- | --- | --- |
| Aluminium (d) | 4–1671 | 276 | Gower *et al*. (1994) |  |  |  | 3650 (*Daphnia pulex*; 48 h^1^ (AlCl_3_); Griffitt *et al*., 2008) |
|  | 6–808 | 150 | Lawlor & Tipping (2003) |  |  |  |  |
|  | 1.02–1330 | 484 | Neal *et al*. (1996) |  |  |  |  |
|  | 37.4 | >176 | Jarvie *et al*. (2000) |  |  |  |  |
|  | 0–470 | >37 | Jarvie *et al*. (2012) |  |  |  |  |
|  | 700–3500 | 14 | Kalender (2010) |  |  |  |  |
|  | 0–1334 | 2693 | Neal & Robson (2000) |  |  |  |  |
|  | 0.95–149.20 | 312 | Neal *et al*. (2000*b*) |  |  |  |  |
| Aluminium | 0–3214 | 298 | Neal *et al*. (2006) |  |  |  |  |
| Aluminium (t) | 0.18–44.9 | 41 | Dixon & Gardner (2014) |  |  |  |  |
| Arsenic (t) | 1.02–3.95 | 312 | Neal *et al*. (2000*b*) |  |  | 3700 (*Daphnia magna*; 21 days^3^, arsenic oxide (As_2_O_3_); Tišler & Zagorc-Končan, 2002) | 376.5 (*Gammarus pulex*; 10 days^3^, arsenic acid (H_3_AsO_4_), disodium salt; Vellinger *et al*., 2013) |
|  | 0.4–2.7 | 19 | Bubb & Lester (1994) |  |  |  |  |
| Arsenic (d) | 8–3500 | 14 | Kalender (2010) |  |  |  |  |
|  | 0.71–40.30 | 312 | Neal *et al*. (2000*b*) |  |  |  | 2400 (*Ceriodaphnia dubia*; 48 h^1^, arsenic; Hu *et al*., 2012) |
|  | 1–33 | 128 | Shepherd *et al*. (2006) |  |  |  |  |
|  | 0.5–0.7 | 15 | Casper *et al*. (2004) |  |  |  |  |
|  | 0–5.606 | 2693 | Neal & Robson (2000) |  |  |  |  |
| Barium (d) | 1.2–16.6 | 150 | Lawlor & Tipping (2003) |  |  |  | 13.5 (*Daphnia magna*; 21 days^1^, barium chloride; Biesinger & Christensen, 2011) |
|  | 12.7–281 | 484 | Neal *et al*. (1996) |  |  |  |  |
|  | 53.1 | >176 | Jarvie *et al*. (2000) |  |  |  |  |
|  | 1–98 | >37 | Jarvie *et al*. (2012) |  |  |  |  |
|  | 8.9–322.5 | 2693 | Neal & Robson (2000) |  |  |  |  |
|  | 8.9–19.55 | 312 | Neal *et al*. (2000*b*) |  |  |  |  |
| Barium | 18–61 | 298 | Neal *et al*. (2006) |  |  |  |  |
| Beryllium (d) | 0–0.14 | 312 | Neal *et al*. (2000*b*) |  |  | 5.5  (*Daphnia magna*; 21 days^3^, beryllium sulfate; Chapman *et al*., 1980) |  |
|  | 0–28.89 | 2693 | Neal & Robson (2000) |  |  |  |  |
| Cadmium (d) | 0.1–7 | 276 | Gower *et al*. (1994) | 12  (*Lymnaea stagnalis*; 14 days^4^, cadmium chloride (CdCl_2_); Crémazy *et al*., 2018) |  | 0.15  (*Daphnia magna*; 21 days^3^, cadmium chloride (CdCl_2_); Chapman *et al*., 1980) | 0.0054 (*Daphnia magna*; 48 h^1^, cadmium oxide; Gale *et al*., 1992) |
|  | 0.01–0.60 | 150 | Lawlor & Tipping (2003) |  |  |  |  |
|  | <0.1 | 9 | Bubb & Lester (1994) |  |  |  |  |
|  | 0.12–0.32 | 15 | Casper *et al*. (2004) |  |  |  |  |
|  | 0.17 | >176 | Jarvie *et al*. (2000) |  |  |  |  |
|  | 0.181– 9.8 |  | Environment Agency (2008*c*) |  |  |  |  |
|  | 1–35 | 14 | Kalender (2010) |  |  |  |  |
|  | 0–3.47 | 2693 | Neal & Robson (2000) |  |  |  |  |
|  | 0.1–3.41 | 128 | Shepherd *et al*. (2006) |  |  |  |  |
|  | 0.05–0.18 | 10 | Buss and Lester (1995) |  |  |  |  |
|  | 0.01–0.1 | >37 | Jarvie *et al*. (2012) |  |  |  |  |
|  | 0–3.54 | 312 | Neal *et al*. (2000*b*) |  |  |  |  |
| Cadmium (t) | 0.01–0.44 | 5 | Lundy *et al*. (2017) | 7  (*Daphnia magna*; 21 days^3^ (CdCl_2_); Enserink *et al*., 1993) | 1  (*Gammarus fossarum*; 21 days^3^ (CdCl_2_); Geffard *et al*., 2010) | 0.2  (*Ceriodaphnia reticulata*; 7 days^3^ (CdCl_2_); Elnabarawy *et al*., 1986) | 0.1  (*Daphnia magna*; 72 h^1^, sulfuric acid, cadmium salt (1:1); Braginskij & Shcherban 1979) |
| Cadmium | 0–1 | 298 | Neal *et al*. (2006) |  |  |  |  |
| Calcium (t) | 52300 | 52 | Neal *et al*. (2000*a*) |  |  | 305000  (*Daphnia magna*; 7 days^3^ (CaCl_2_); Taylor *et al*., 1988) | 690  (*Corbicula fluminea*; 10 days^1^, hypochlorous acid, calcium salt (2:1); Cherry *et al*., 1980) |
| Calcium (d) | 101–138 | 312 | Neal *et al*. (2000*b*) |  |  |  |  |
|  | 150300–4380000 | 14 | Kalender (2010) |  |  |  |  |
|  | 6000–165000 | 2693 | Neal & Robson (2000) |  |  |  |  |
|  | 1900–75000 |  | Jarvie *et al*. (2012) |  |  |  |  |
| Calcium | 80000–149000 | 298 | Neal *et al*. (2006) |  |  |  |  |
| Cerium (d) | 0.01–0.84 | 312 | Neal *et al*. (2000*b*) |  |  |  |  |
|  | 0–1.44 | 2693 | Neal & Robson (2000) |  |  |  |  |
|  | 0.002–0.6 | >37 | Jarvie *et al*. (2012) |  |  |  |  |
| Chromium (d) | 0.0824–1.3 | 60 | Bearcock *et al*. (2017) |  |  | 66  (*Daphnia magna*; 21 days^3^, nitric acid, chromium(3+) salt); Chapman *et al*., 1980) | 390  (*Austropotamobius pallipes*; 96 h^1^ (CrCl_3_); Vareille-Morel (1982) |
|  | 0– 11.8 | 484 | Neal *et al*. (1996) |  |  |  |  |
|  | 0.5 – 1 | 9 | Bubb & Lester (1994) |  |  |  |  |
|  | 123–2230 | 32 | Palumbo-Roe *et al*. (2017) |  |  |  |  |
|  | 0.5–204 | 128 | Shepherd *et al*. (2006) |  |  |  |  |
|  | 0.008–75 | 14 | Kalender (2010) |  |  |  |  |
|  | 0–46.8 | 2693 | Neal & Robson (2000) |  |  |  |  |
|  | 2.34 | >176 | Jarvie *et al*. (2000) |  |  |  |  |
|  | 0–1.73 | >37 | Jarvie *et al*. (2012) |  |  |  |  |
|  | 0.16–2.28 | 312 | Neal *et al*. (2000*b*) |  |  |  |  |
| Cobalt (d) | 0.79 | >176 | Jarvie *et al*. (2000) | 79 (*Lymnaea stagnalis*; 28 days^3^, cobalt chloride; De Schamphelaere *et al*., 2008) |  |  | 1670 (*Ceriodaphnia dubia*; 48 h^2^, cobalt chloride; Griffitt *et al*., 2008) |
|  | 0.04–5.75 | 2693 | Neal & Robson (2000) |  |  |  |  |
|  | 1–45 | 14 | Kalender (2010) |  |  |  |  |
| Copper (d) | <1–1271 | 276 | Gower *et al*. (1994) | 3.71  (*Lymnaea stagnalis*; 14 days^4^ (CuCl_2_); Crémazy *et al*., 2018) |  | 9.5  (*Daphnia magna*; 21 days^3^ (CuCl_2_); Chapman *et al*., 1980) | 0.03  (*Daphnia magna*; 48 h^1^, sulfuric acid copper(2+) salt (1:1); Ryan *et al*., 2009) |
|  | 0.1–3 | 150 | Lawlor & Tipping (2003) |  |  |  |  |
|  | 0.8–15.6 | 484 | Neal *et al*. (1996) |  |  |  |  |
|  | 7.95 | >176 | Jarvie *et al*. (2000) |  |  |  |  |
|  | 1–5.5 | 9 | Bubb & Lester (1994) |  |  |  |  |
|  | 7.4–15.65 | 15 | Casper *et al*. (2004) |  |  |  |  |
|  | 0–97 | 2693 | Neal & Robson (2000) |  |  |  |  |
|  | 2.5–350 | 14 | Kalender (2010) |  |  |  |  |
|  | 2.07–10.35 | 312 | Neal *et al*. (2000*b*) |  |  |  |  |
|  | 1–12.3 | 10 | Buss & Lester (1995) |  |  |  |  |
|  | 0.79–4.34 | 32 | Lathouri & Korre (2015) |  |  |  |  |
|  | 1.29–70.9 | 128 | Shepherd *et al*. (2006) |  |  |  |  |
| Copper (t) | 0.5–145 | >3600 | Rothwell *et al*. (2010) | 5.5  (*Daphnia longispina*; 21 days^3^ (CuCl_2_); Agra *et al*., 2011) | 1.25  (*Dreissena polymorpha*; 48 h^2^ (CuCl_2_);  Faria *et al*., 2010) | 1.8  (*Ceriodaphnia dubia*; 7 days^2^, sulfuric acid, copper(1+) salt (1:2);  Cooper *et al*., 2009) | 0.021  (*Lumbriculus variegatus*; 48 h^1^ (CuCl_2_);  Meyer *et al*., 2002) |
|  | 1.04–46.7 | 5 | Lundy *et al*. (2017) |  |  |  |  |
| Copper | 0–14 | 298 | Neal *et al*. (2006) |  |  |  |  |
| Iron (d) | 5.99–1060 | 484 | Neal *et al*. (1996) |  |  |  |  |
|  | 77 | >176 | Jarvie *et al*. (2000) |  |  |  |  |
|  | 11–1360 | >37 | Jarvie *et al*. (2012) |  |  |  |  |
|  | 168–10000 | 14 | Kalender 2010) |  |  |  |  |
|  | 0–32.52 | 2693 | Neal & Robson (2000) |  |  |  |  |
|  | 13–42 | 10 | Buss and Lester (1995) |  |  |  |  |
|  | 3.64–199.52 | 312 | Neal *et al*. (2000b) |  |  |  |  |
| Iron (t) | 30–430000 | >4560 | Rothwell *et al*. (2010) | 1310  (*Daphnia pulex*; 21 days^3^ (FeCl_3_);  Birge *et al*., 1985) |  | 1310  (*Daphnia pulex*; 21 days^3^ (FeCl_3_); Birge *et al*., 1985) | 17  (*Daphnia magna*; 48 h^1^ (FeCl_3_); Oda *et al*. 2006) |
|  | 35–203 | 32 | Palumbo-Roe *et al*. (2017) |  |  |  |  |
| Iron | 14–3621 | 298 | Neal *et al*. (2006) |  |  |  |  |
| Gadolinium (d) | 0–0.04 | 312 | Neal *et al*. (2000*b*) |  |  |  |  |
|  | 0–0.25 | 2693 | Neal & Robson (2000) |  |  |  |  |
| Lanthanum (d) | 0–3.63 | 312 | Neal *et al*. (2000*b*) |  |  |  |  |
|  | 0–1.03 | 2693 | Neal & Robson (2000) |  |  |  |  |
| Lithium (d) | 5.96–21.13 | 312 | Neal *et al*. (2000*b*) |  |  |  |  |
|  | 33.4 | >176 | Jarvie *et al*. (2000) |  |  |  |  |
|  | 0.36–224.1 | 2693 | Neal & Robson (2000) |  |  |  |  |
|  | 0.78–101 | 484 | Neal *et al*. (1996) |  |  |  |  |
| Lithium | 5–20 | 298 | Neal *et al*. (2006) |  |  |  |  |
| Lead (d) | <5–332 | 276 | Gower *et al*. (1994) | 1.5  (*Lymnaea stagnalis*; 14 days^5^, nitric acid, lead(2+) salt (2:1); Esbaugh *et al*., 2012) |  | 5.1  (*Ceriodaphnia dubia*; 7 days^5^, nitric acid, lead(2+) salt (2:1); Mager *et al*., 2011) | 28.8  (*Ceriodaphnia dubia*; 48 h^1^, nitric acid, lead(2+) salt (2:1); Esbaugh *et al*., 2011) |
|  | <0.05–19.4 | 60 | Bearcock *et al*. (2017) |  |  |  |  |
|  | 0.05–1.04 | 150 | Lawlor & Tipping (2003) |  |  |  |  |
|  | 1.49 | >176 | Jarvie *et al*. (2000) |  |  |  |  |
|  | 0–32.5 | 484 | Neal *et al*. (1996) |  |  |  |  |
|  | 0.2–439.9 | 15 | Valencia-Avellan *et al*. (2017) |  |  |  |  |
|  | 4.9–175 |  | Environment Agency (2008*c*) |  |  |  |  |
|  | 2–500 |  | Mayes *et al*. (2010) |  |  |  |  |
|  | 0–43.35 | 2693 | Neal & Robson (2000) |  |  |  |  |
|  | 5–285 | 14 | Kalender (2010) |  |  |  |  |
|  | 0.96–6.15 | 36 | Palumbo-Roe *et al*. (2012) |  |  |  |  |
|  | 0.4–158 | 128 | Shepherd *et al*. (2006) |  |  |  |  |
|  | 0.65–1.65 | 10 | Buss & Lester (1995) |  |  |  |  |
|  | 0.06–4.46 | 312 | Neal *et al*. (2000*b*) |  |  |  |  |
| Lead (t) | 0.4–345 | >3720 | Rothwell *et al*. (2010) |  |  | 4.5  (*Ceriodaphnia dubia*; 7 days^3^, nitric acid, lead(2+) salt (2:1); Cooper *et al*., 2009) | 12  (*Daphnia magna*; 48 h^1^, nitric acid, lead(2+) salt (2:1); Sankaramanachi & Qasim, 1999) |
|  | 0.05–2701.2 | 15 | Valencia-Avellan *et al*. (2017) |  |  |  |  |
|  | 0.21–13.3 | 5 | Lundy *et al*. (2017) |  |  |  |  |
| Magnesium (d) | 200–1300 | 150 | Lawlor & Tipping (2003) |  |  |  |  |
|  | 41100–4343000 | 14 | Kalender (2010) |  |  |  |  |
|  | 1000–90000 | 2693 | Neal & Robson (2000) |  |  |  |  |
|  | 700–20000 | >37 | Jarvie *et al*. (2012) |  |  |  |  |
| Magnesium (t) | 17770 | 52 | Neal *et al*. (2000*a*) |  |  |  | 32000  (*Daphnia hyaline*; 48 h^1^,  magnesium chloride; Baudouin & Scoppa, 1974) |
| Magnesium | 4600–6900 |  | Neal *et al*. (2000*b*) |  |  |  |  |
|  | 3700–8900 | 298 | Neal *et al*. (2006) |  |  |  |  |
| Manganese (d) | 0.2–64.8 | 150 | Lawlor & Tipping (2003) |  |  |  |  |
|  | 1.2–532 | 484 | Neal *et al*. (1996) |  |  |  |  |
|  | 0–531.8 | 2693 | Neal & Robson (2000) |  |  |  |  |
|  | 24.5 | >176 | Jarvie *et al*. (2000) |  |  |  |  |
|  | 33.7–100 | 36 | Palumbo-Roe *et al*. (2017) |  |  |  |  |
|  | 11.2–17.5 | 10 | Buss & Lester (1995) |  |  |  |  |
|  | 140–3000 | 14 | Kalender (2010) |  |  |  |  |
|  | 2–172 | >37 | Jarvie *et al*. (2012) |  |  |  |  |
|  | 1.48–18.88 | 312 | Neal *et al*. (2000*b*) |  |  |  |  |
| Manganese | 3–34 | 298 | Neal *et al*. (2006) |  |  |  |  |
| Mercury (t) | 0.15–0.38 | 9 | Bubb & Lester (1994) | 0.407  (*Daphnia magna*; 28 days^3^ (HgCl_2_); Tsui & Wang, 2005) | 0.73  (*Dreissena polymorpha*; 48 h^2^ (HgCl_2_); Faria *et al*. 2010) | 12.6  (*Ceriodaphnia dubia*; 7 days^2^, mercuric nitrate; Spehar & Fiandt, 1986) | 1  (*Moina macrocopa*; 48 h^1^, mercuric nitrate; Pokethitiyook *et al*., 1987) |
|  | 0.01–0.41 | 5 | Lundy *et al*. (2017) |  |  |  |  |
| Mercury (d) | 0.01–0.191 | 128 | Shepherd *et al*. (2006) | 32 (*Daphnia magna*; 21 days^3^, mercuric nitrate; Enserink *et al*., 1991) |  |  | 7 (*Daphnia magna*; 21 days^3^, mercuric nitrate; Enserink *et al*., 1991) |
| Molybdenum (d) | 0–54 | 484 | Neal *et al*. (1996) |  |  |  |  |
|  | 6.51 | >176 | Jarvie *et al*. (2000) |  |  |  |  |
|  | 0.05–3.9 | >37 | Jarvie *et al*. (2012) |  |  |  |  |
|  | 0–70.28 | 2693 | Neal & Robson (2000) |  |  |  |  |
|  | 0.91–1588.14 | 312 | Neal *et al*. (2000*b*) |  |  |  |  |
| Molybdenum | 0–649 | 298 | Neal *et al*. (2006) |  |  |  |  |
| Neodymium (d) | 0–0.71 | 312 | Neal *et al*. (2000*b*) |  |  |  |  |
|  | 0–1.02 | 2693 | Neal & Robson (2000) |  |  |  |  |
| Nickel (d) | 0.1–1.2 | 150 | Lawlor & Tipping (2003) | 115  (*Lymnaea stagnalis*; 14 days^4^ (NiCl_2_); Crémazy *et al*., 2018) |  | 1.3  (*Ceriodaphnia dubia*; 10 days^4^, nickel; De Schamphelaere *et al*., 2006) | 3.8  (*Ceriodaphnia dubia*; 7 days^3^ (NiCl_2_); Keithly *et al*., 2004) |
|  | 0.5 –21.3 | 484 | Neal *et al*. (1996) |  |  |  |  |
|  | 1.5–7 | 9 | Bubb & Lester (1994) |  |  |  |  |
|  | 3.4–400 | 14 | Kalender (2010) |  |  |  |  |
|  | 30.9 | >176 | Jarvie *et al*. (2000) |  |  |  |  |
|  | 0.3–5.4 | >37 | Jarvie *et al*. (2012) |  |  |  |  |
|  | 5–304 | 128 | Shepherd *et al*. (2006) |  |  |  |  |
|  | 0.36–45.37 | 2693 | Neal & Robson (2000) |  |  |  |  |
|  | 1.67–4.74 | 312 | Neal *et al*. (2000*b*) |  |  |  |  |
| Nickel (t) | 0.66–55.1 | 5 | Lundy *et al*. (2017) | 100  (*Daphnia magna*; 21 days^3^, nickel; Munzinger & Monicelli, 1991) |  | 100  (*Daphnia magna*; 21 days^3^, nickel; Munzinger & Monicelli, 1991) | 0.00036  (*Paramecium bursaria*; 24 h^1^, nickel chloride; Madoni, 2000) |
| Nickel | 1–15 | 298 | Neal *et al*. (2006) |  |  |  |  |
| Potassium (d) | 0–500 | 150 | Lawlor & Tipping (2003) |  |  |  |  |
|  | 10000–450000 | 14 | Kalender (2010) |  |  |  |  |
|  | 0–32000 | 2693 | Neal & Robson (2000) |  |  |  |  |
|  | 400–23000 | >37 | Jarvie *et al*. (2012) |  |  |  |  |
| Potassium (t) | 6430 | 52 | Neal *et al*. (2000*a*) |  |  |  |  |
| Potassium | 3800–11800 | 312 | Neal *et al*. (2000*b*) |  |  |  |  |
|  | 5300–14400 | 298 | Neal *et al*. (2006) |  |  |  |  |
| Rubidium (d) | 0.75–17.3 | 484 | Neal *et al*. (1996) |  |  |  |  |
|  | 9.99 | >176 | Jarvie *et al*. (2000) |  |  |  |  |
|  | 0.8–7.6 | >37 | Jarvie *et al*. (2012) |  |  |  |  |
|  | 0–37.77 | 2693 | Neal & Robson (2000) |  |  |  |  |
|  | 1.4–7.2 | 312 | Neal *et al*. (2000*b*) |  |  |  |  |
| Samarium (d) | 0–0.04 | 312 | Neal *et al*. (2000*b*) |  |  |  |  |
|  | 0–0.55 | 2693 | Neal & Robson (2000) |  |  |  |  |
| Samarium (t) | 0–0.21 | 312 | Neal *et al*. (2000*b*) |  |  |  |  |
| Scandium (d) | 0–15.08 | 2693 | Neal & Robson (2000) |  |  |  |  |
|  | 0.32–2.65 | 312 | Neal *et al*., 2000*b*) |  |  |  |  |
| Scandium (t) | 0.34–2.54 | 312 | Neal *et al*. (2000*b*) |  |  |  |  |
| Silver (d) | 0.045–0.25 (WWTP effluent) | 750 | Chemical Investigations Programme (CIP) (Gardner *et al*., 2022) | 1.48  (*Lymnaea stagnalis*; 14 days^4^, nitric acid silver (1+) salt (1:1); Crémazy *et al*., 2018) |  | 0.63  (*Daphnia magna*; 21 days^3^, nitric acid silver(1+) salt (1:1); Kolkmeier & Brooks, 2013) | 0.24  (*Daphnia magna*; 48 h^1^, silver; Chapman *et al*., 1980) |
| Silver (t) | 0.045–0.29 (WWTP effluent) | 750 | Chemical Investigations Programme (CIP) Gardner *et al*. (2022) | 6.42  (*Daphnia magna*; 21 days^4^, nitric acid silver(1+) salt (1:1); Bianchini & Wood, 2008) | 200  (*Chironomus riparius*; 21 days^3^, silver; Nair *et al*., 2011) | 0.01  (*Ceriodaphnia dubia*; 8 days^3^, nitric acid silver(1+) salt (1:1); Bielmyer *et al*., 2002) | 0.06  (*Ceriodaphnia dubia*; 48 h^7^, nitric acid silver(1+) salt (1:1); Tsui *et al*., 2005) |
| Sodium (d) | 1600–6600 | 150 | Lawlor & Tipping (2003) |  |  |  |  |
|  | 45500–2000000 | 14 | Kalender (2010) |  |  |  |  |
|  | 1800–300000 | 2693 | Neal & Robson (2000) |  |  |  |  |
|  | 3500–154000 | >37 | Jarvie *et al*. (2012) |  |  |  |  |
| Sodium (t) | 43200 | 52 | Neal *et al*. (2000*a*) |  |  |  |  |
| Sodium | 15600–63900 | 312 | Neal *et al*. (2000*b*) |  |  |  |  |
|  | 12600 – 58600 | 298 | Neal *et al*. (2006) |  |  |  |  |
| Strontium (d) | 63.1–750 | 484 | Neal *et al*. (1996) |  |  |  |  |
|  | 585 | >176 | Jarvie *et al*. (2000) |  |  |  |  |
|  | 8–279 | >37 | Jarvie *et al*. (2012) |  |  |  |  |
|  | 22.2–805.2 | 2693 | Neal & Robson (2000) |  |  |  |  |
|  | 297.6–429.70 | 312 | Neal *et al*. (2000*b*) |  |  |  |  |
| Strontium | 335–975 | 298 | Neal *et al*. (2006) |  |  |  |  |
| Tin (t) | 0.06–13.9 | 5 | Lundy *et al*. (2017) |  |  |  | 3600  (*Chironomus plumosus*; 24 h^1^, tin chloride; Fargašová, 1997) |
| Tin (d) | 0–0.55 | 312 | Neal *et al*. (2000b) |  |  |  |  |
|  | 0–12.99 | 2693 | Neal & Robson (2000) |  |  |  |  |
|  | 0.23 | >176 | Jarvie *et al*. (2000) |  |  |  |  |
| Titanium (d) | 0.2–17 | >37 | Jarvie *et al*. 2012) |  |  |  |  |
| Uranium (d) | 0.54–1.23 | 312 | Neal *et al*. (2000*b*) | 1930  (*Chironomus tentans*; 10 days^6^, nitric acid, uranium salt;  Liber *et al*., 2007) | 230  (*Corynoneura* sp.; 28 days^6^, nitric acid, uranium salt;  Liber *et al*., 2007) | 830  (*Daphnia magna*; 21 days^6^, nitric acid, uranium salt;  Liber *et al*., 2007) | 830 (*Corynoneura* sp.; 28 days^1^, nitric acid, uranium salt; Liber *et al*., 2007) |
|  | 0–3.37 | 2693 | Neal & Robson (2000) |  |  |  |  |
|  | 1.21 | >176 | Jarvie *et al*. (2000) |  |  |  |  |
| Vanadium | 0–7 | 298 | Neal *et al*. (2006) |  |  |  |  |
| Vanadium (d) | 0.1–2.6 | >37 | Jarvie *et al*. (2012) |  |  |  |  |
| Yttrium (d) | 0.01–0.14 | 312 | Neal *et al*. (2000*b*) |  |  |  |  |
|  | 0–1.76 | 2693 | Neal & Robson (2000) |  |  |  |  |
|  | 0.05 | >176 | Jarvie *et al*. (2000) |  |  |  |  |
| Zinc (t) | 5–525 | >25200 | Neal *et al*. (2000*a*) | 34  (*Corbicula sp*.; 30 days^3^, sulfuric acid, zinc salt (1:1); Farris *et al*., 1989) |  | 21.8  (*Ceriodaphnia dubia*; 7 days^2^, sulfuric acid, zinc salt (1:1); Cooper *et al*., 2009) | 5  (*Asellus aquaticus*; 72 h^1^, sulfuric acid, zinc salt (1:1); Braginskiy & Shcherban (1978) |
|  | 66.5–12619.3 | 15 | Valencia-Avellan *et al*. (2017) |  |  |  |  |
|  | 1.25–62.7 | 5 | Lundy *et al*. (2017) |  |  |  |  |
|  | 2–2660 | 276 | Gower *et al*. (1994) | 115  (*Daphnia magna*; 21 days^3^, zinc chloride; Muyssen & Janssen (2007) |  | 14  (*Ceriodaphnia dubia*; 7 days^4^, zinc chloride; Nys *et al*., 2017) | 53  (*Ceriodaphnia dubia*; 7 days^1^, zinc chloride; Besser *et al*., 2021) |
| Zinc (d) | 0.2–11.8 | 150 | Lawlor & Tipping (2003) |  |  |  |  |
|  | 0.25–12.33 | 2693 | Neal & Robson (2000) |  |  |  |  |
|  | 33.6 | >176 | Jarvie *et al*. (2000) |  |  |  |  |
|  | 67.8–7428.4 | 15 | Valencia-Avellan *et al*. (2017) |  |  |  |  |
|  | 3.77–95.2 | 484 | Neal *et al*. (1996) |  |  |  |  |
|  | 4–24 | 9 | Bubb & Lester (1994) |  |  |  |  |
|  | 14–690 | 14 | Kalender (2010) |  |  |  |  |
|  | 9.14–3040 |  | Environment Agency (2008*c*) |  |  |  |  |
|  | 2.31–70.25 | 312 | Neal *et al*. (2000*b*) |  |  |  |  |
|  | 8.87–429 | 128 | Shepherd *et al*. (2006) |  |  |  |  |
| Zinc | 0–335 | 298 | Neal *et al*. (2006) |  |  |  |  |

^1^LC_50_, lethal concentration for 50%; ^2^EC_50_, effect concentration for 50%; ^3^LOEC, lowest observed effect concentration; ^4^EC_10_, effect concentration for 10%; ^5^EC_20_, effect concentration for 20%; ^6^EC_25_, effect concentration for 25%; ^7^LC_100_, lethal concentration for 100%. (t) = total; (d) dissolved. WwTP, wastewater treatment plant.

**Table S3.** Concentrations of petrochemicals recorded across British rivers (wastewater treatment effluents) and lowest effect concentrations (ECs) for British freshwater invertebrates based on laboratory tests. Petrochemicals highlighted in green are included in Table 3.

| **Chemical** | **Field concentrations (µg/l)** | **Number of samples** | **References** | **EC for growth (µg/l)** | **EC for development (µg/l)** | **EC for reproduction (µg/l)** | **EC for mortality (µg/l)** |
| --- | --- | --- | --- | --- | --- | --- | --- |
| Benzene | <0.7–35.4 | 97 | Levy *et al*. (1999) |  |  |  | 10000  (*Ischnura elegans*; 48 h^1^) |
|  | <10–25.3 | 348 |  |  |  |  |  |
| 1,2-Dimethylbenzene (o-xylene) | 0.15–377 | 4087 | Comber *et al*. (2012) |  |  |  |  |
| Dimethylbenzenes (m/p/o-xylene isomers) | 0.31–150 | 3001 | Comber *et al*. (2012) |  |  |  | 2972.6872 (*Ceriodaphnia dubia*; 7 days^1^) |
| Xylene (m & p) (1,3 + 1,4-dimethylbenzene) | 1.41–2750 | 3830 | Comber *et al*. (2012) |  |  |  | 2972.6872 (*Ceriodaphnia dubia*; 7 days^1^) |
| Biphenyl | 1.3–3.94 | 2389 | Comber *et al*. (2012) | 560  (*Daphnia magna*; 21 days^3^) |  | 330 (*Daphnia magna*; 21 days^3^) | 260.42 (*Daphnia magna*; 48 h^4^) |
| Fluoranthene | 7 | 9006 | Spurgeon *et al*. (2021) | 194 (*Daphnia magna*; 7 days^2^) | 88  (*Chironomus riparius*; 21.2 days^3^) | 1.5  (*Daphnia magna*; 21 days^3^) | 1.2  (*Lumbriculus variegatus*; 96 h^1^) |
| Pyrene | 1.2 | 8970 | Spurgeon *et al*. (2021) | 72.7  (*Daphnia magna*; 7 days^2^) |  |  | 4.328  (*Daphnia magna*; 48 h^1^) |
| Benzophenone | 234.7 | 3244 | Spurgeon *et al*. (2021) |  |  |  | 7600 (*Ceriodaphnia dubia*; 24h^1^) |
| Bis(2-ethylhexyl)phthalate (DEHP) | 170 | 2355 | Spurgeon *et al*. (2021) |  | 1 (*Chironomus riparius*; 10 days^3^) | 390.5633 (*Daphnia magna*; 30 days^3^) | 5 (*Daphnia magna*; 21 days^1^) |
| Benzo[a]pyrene | 1.1 | 548 | Spurgeon *et al*. (2021) |  |  |  | 0.981507 (*Daphnia magna*; 48 h^2^) |
| Dibenz[a,h]anthracene | 21 | 10 | Spurgeon *et al*. (2021) |  |  |  |  |
| Benz[a]anthracene | 1 | 2049 | Spurgeon *et al*. (2021) |  |  |  | 0.958832 (*Daphnia magna*; 48 h^1^) |

^1^LC_50_, lethal concentration for 50%; ^2^EC_50_, effect concentration for 50%; ^3^LOEC, lowest observed effect concentration; ^4^EC_01_, effect concentration for 1%. All EC data were obtained from the *ECOTOX* database, unless specified otherwise.

**Table S4.** Concentrations of pharmaceuticals and personal care products recorded across British rivers (wastewater treatment effluents) and lowest effect concentrations (ECs) for British freshwater invertebrates. Rows highlighted in green are included in Table 4.

| **Chemical** | **Field concentrations (µg/l)** | **Number of samples** | **References** | **EC for growth (µg/l)** | **EC for development (µg/l)** | **EC for reproduction (µg/l)** | **EC for mortality (µg/l)** |
| --- | --- | --- | --- | --- | --- | --- | --- |
| 1,7-Dimethylxanthine | 0.9235 |  | Proctor *et al*. (2019) |  |  |  |  |
|  | 0.22693–0.024389 | 3 | Sims *et al*. (2023) |  |  |  |  |
| 3,4,5,6-Tetrabromo-o-cresol | <0.001–0.170 | >160 | Kasprzyk-Hordern *et al*. (2008) |  |  |  |  |
| 3,4-Methylenedioxymethamphetamine (MDMA) (illicit drug) | 0.004–0.044 | 171 |  |  |  |  |  |
|  | 0.00121–0.00124 | 3 | Sims *et al*. (2023) |  |  |  |  |
|  | 0.0043 |  | Proctor *et al*. (2019) |  |  |  |  |
| 4-Chloroxylenol | <0.03–0.358 | >160 | Kasprzyk-Hordern *et al*. (2008) |  |  |  |  |
| 5-Aminosalicylic acid | <0.015–0.190 | >160 | Kasprzyk-Hordern *et al*. (2008) |  |  |  |  |
| 10,11-epoxy-carbamazepine | <0.0001–0.0693 | 957 | Buglife (2021) |  |  |  |  |
|  | 0.005–0.111 | 355 | Egli *et al*. (2023) |  |  |  |  |
|  | 0.0309 |  | Proctor *et al*. (2019) |  |  |  |  |
|  | 0.00447–0.00448 | 3 | Sims *et al*. (2023) |  |  |  |  |
|  | 0.0056–0.018 |  | Ramage *et al*. (2019) |  |  |  |  |
| 10,11-Dihydro-10-hydroxycarbamazepine | 0.0057 |  | Proctor *et al*. (2019) |  |  |  |  |
|  | 0.00509–0.00538 | 3 | Sims *et al*. (2023) |  |  |  |  |
| 17-Alpha-ethinyloestradiol (EE2) | <0.000015–0.0049 | 1037 | Buglife (2021) | 800 (*Chironomus tentans*; 10 days^4^) | 3100 (*Chironomus tentans*; 42 days^3^) | 0.025 (*Potamopyrgus antipodarum*; 63 days^3^) | 300 (*Ceriodaphnia reticulata*; 34 days^3^) |
| 17-Beta-oestradiol (E2) | <0.00015–0.0119 | 995 | Buglife (2021) |  |  |  | 1600 (*Nitocra spinipes*; 96h^1^; Breitholtz & Bengtsson, 2001) |
| Acyclovir | 0.0079 | 24 | Burns *et al*. (2017) |  |  |  |  |
| Acetyl-sulfamethoxazole | <0.05–0.239 | 30 | Ashton *et al*. (2004) |  |  |  |  |
|  | <0.05–0.24 | 2 | Hilton & Thomas (2003) |  |  |  |  |
| Anhydroecgonine methyl ester (illicit drug) | 0.00311–0.00312 | 3 | Sims *et al*. (2023) |  |  |  |  |
| Amitriptyline | <0.0005–0.021 | >160 | Kasprzyk-Hordern *et al*. (2008) |  |  |  | 804.48697 (*Brachionus calyciflorus*; 24h^1^) |
|  | 0.0012–0.027 | 132 | Burns *et al*. (2018) |  |  |  |  |
|  | <0.00866 | 8 | Wilkinson *et al*. (2019) |  |  |  |  |
|  | 0.005–0.108 | 196 | Egli *et al*. (2023) |  |  |  |  |
|  | 0.0103 |  | Proctor *et al*. (2019) |  |  |  |  |
| Amlodipine | 0.007–0.029 | 13 | Egli *et al*. (2023) |  |  |  |  |
| Amoxycillin | <0.01–0.622 | >100 | Kasprzyk-Hordern *et al*. (2008) |  |  | 27200 (*Daphnia magna*; 21 days^7^; Lee *et al*., 2021) |  |
| Amphetamine (illicit drug) | <0.001–0.021 | >160 | Kasprzyk-Hordern *et al*. (2008) |  |  |  | 3102.65592 (*Brachionus calyciflorus*; 24 h^1^) |
|  | 0.03885–0.0863 | 3 | Sims *et al*. (2023) |  |  |  |  |
| S(+)-amphetamine | 0.2 | 1 | Ramage *et al*. (2019) |  |  |  |  |
| R(−)-amphetamine | 0.27 | 1 | Ramage *et al*. (2019) |  |  |  |  |
| Aspirin | <0.0005–0.036 | >160 | Kasprzyk-Hordern *et al*. (2008) | 1800 (*Daphnia magna*; 21 days^3^) |  | 1800 (*Daphnia magna*; 21 days^3^) | 141425.6785 (*Brachionus calyciflorus*; 24 h^1^) |
| Atazanavir | 0.23 | 12 | Spurgeon *et al*. (2021) |  |  |  |  |
| Atenolol | <0.001–0.56 | >160 | Kasprzyk-Hordern *et al*. (2008) |  |  | 8870 (*Daphnia magna*; 21 days^3^) | 755000  (*Daphnia magna*; 48 h^1^) |
|  | 0.0101–0.1 | 132 | Burns *et al*. (2018) |  |  |  |  |
|  | 1 | 2383 | Spurgeon *et al*. (2021) |  |  |  |  |
|  | <0.0115–0.197 | 8 |  |  |  |  |  |
|  | 0.0539 |  | Proctor *et al*. (2019) |  |  |  |  |
|  | 0.01743–0.01754 | 3 | Sims *et al*. (2023) |  |  |  |  |
|  | 0.025 | 24 | Burns *et al*. (2017) |  |  |  |  |
| S(−)-atenolol | 0.0039–0.02 | 6 | Ramage *et al*. (2019) |  |  |  |  |
| R(+)-atenolol | 0.0034–0.019 | 6 | Ramage *et al*. (2019) |  |  |  |  |
| Atorvastatin | 0.005–0.217 | 161 | Egli *et al*. (2023) | 5600 (*Chironomus tentans*; 10 days^4^) |  |  | 8200 (*Chironomus tentans*; 10 days^4^) |
|  | 0.0444 |  | Proctor *et al*. (2019) |  |  |  |  |
| Azathioprine | 0.02142–0.02146 | 3 | Sims *et al*. (2023) |  |  |  |  |
| Azithromycin | 0.87 | 450 | Spurgeon *et al*. (2021) |  |  |  |  |
|  | <0.0001–0.221 | 961 | Buglife (2021) |  |  |  |  |
|  | 0.017–0.298 | 25 | Egli *et al*. (2023) |  |  |  |  |
|  | 0.0039 |  | Proctor *et al*. (2019) |  |  |  |  |
| Bisoprolol | 0.004–0.075 | 368 | Egli *et al*. (2023) |  |  |  |  |
| Bendroflumethiazide | <0.0005–0.015 | >160 | Kasprzyk-Hordern *et al*. (2008) |  |  |  |  |
| Benzophenone-1 | <0.0003–0.017 | >160 | Kasprzyk-Hordern *et al*. (2008) |  |  |  |  |
| Benzophenone-2 | <0.0005–0.284 | >160 | Kasprzyk-Hordern *et al*. (2008) |  |  |  |  |
| Benzophenone-3 | <0.015–0.044 | >160 | Kasprzyk-Hordern *et al*. (2008) |  |  |  |  |
|  | 0.02079–0.02197 | 3 | Sims *et al*. (2023) |  |  |  |  |
|  | 0.019 |  | Proctor *et al*. (2019) |  |  |  |  |
| Benzophenone-4 | <0.003–0.371 | >160 | Kasprzyk-Hordern *et al*. (2008) |  |  |  |  |
|  | 0.29875–0.30533 | 3 | Sims *et al*. (2023) |  |  |  |  |
|  | 0.607 |  | Proctor *et al*. (2019) |  |  |  |  |
| Benzoylecgonine (illicit drug) | <0.001–0.092 | >160 | Kasprzyk-Hordern *et al*. (2008) |  |  |  |  |
|  | 0.004–0.418 | 369 | Egli *et al*. (2023) |  |  |  |  |
|  | 0.04193–0.0428 | 3 | Sims *et al*. (2023) |  |  |  |  |
|  | 0.0454 |  | Proctor *et al*. (2019) |  |  |  |  |
| Bezafibrate | <0.001–0.076 | >160 | Kasprzyk-Hordern *et al*. (2008) |  |  |  | 60910 (*Brachionus calyciflorus*; 24h^1^) |
|  | 39 | 215 | Spurgeon *et al*. (2021) |  |  |  |  |
|  | 0.03876–0.03884 | 3 | Sims *et al*. (2023) |  |  |  |  |
|  | 0.005–0.066 | 231 | Egli *et al*. (2023) |  |  |  |  |
|  | 0.1036 |  | Proctor *et al*. (2019) |  |  |  |  |
| Bicalutamide | 0.0594 |  | Proctor *et al*. (2019) |  |  |  |  |
| Butylparaben | <0.0003–0.052 | >160 | Kasprzyk-Hordern *et al*. (2008) |  |  |  |  |
|  | 0.00161–0.00192 | 3 | Sims *et al*. (2023) |  |  |  |  |
| Buprenorphine | 0.0063 |  | Proctor *et al*. (2019) |  |  |  |  |
| Benzatropine | 0.005–0.023 | 17 | Proctor *et al*. (2019) |  |  |  |  |
| Carbamazepine | <0.0005–0.647 | >160 | Kasprzyk-Hordern *et al*. (2008) | 2600 (*Chironomus tentans*; 10 days^4^) | 332 (*Chironomus riparius*; 28 days^3^) | 100 (*Ceriodaphnia dubia*; 7 days^3^) | 754 (*Brachionus calyciflorus*; 48 h^3^) |
|  | 0.0001–0.195 | 132 | Burns *et al*. (2018) |  |  |  |  |
|  | 0.001–0.222 | 124 | Niemi *et al*. (2022) |  |  |  |  |
|  | 0.053–0.267 |  | Zhou & Broodbank (2014) |  |  |  |  |
|  | 1.5 | 2578 | Spurgeon *et al*. (2021) |  |  |  |  |
|  | <0.0005–1.34 | 961 | Buglife (2021) |  |  |  |  |
|  | <0.00602–0.219 | 8 | Wilkinson *et al*. (2019) |  |  |  |  |
|  | 0.005–0.657 | 379 | Egli *et al*. (2023) |  |  |  |  |
|  | 0.1604 |  | Proctor *et al*. (2019) |  |  |  |  |
|  | 0.0091–0.015 | 6 | Ramage *et al*. (2019) |  |  |  |  |
|  | 0.02–0.14 |  | Zhang & Zhou (2007) |  |  |  |  |
|  | 0.05066–0.05074 | 3 | Sims *et al*. (2023) |  |  |  |  |
|  | 0.022–0.027 | 24 | Burns *et al*. (2017) |  |  |  |  |
| Cetirizine | 53 | 1820 | Spurgeon *et al*. (2021) |  |  |  |  |
|  | 0.2396 |  | Proctor *et al*. (2019) |  |  |  |  |
| Chloramphenicol | <0.002–0.04 | >160 | Kasprzyk-Hordern *et al*. (2008) |  | 85180 (*Daphnia magna*; 72 h^2^; Zhang *et al*., 2021) |  | 2074511.934 (*Brachionus calyciflorus*; 24 h^1^) |
|  | 0.006–0.028 | 21 | Egli *et al*. (2023) |  |  |  |  |
| Chlorpheniramine | 0.0024 | 24 | Burns *et al*. (2017) |  |  |  |  |
| Chlorophene | <0.003–0.016 | >160 | Kasprzyk-Hordern *et al*. (2008) |  |  |  | 0.00012 (*Brachionus calyciflorus*; 24 h^1^) |
| Cimetidine | <0.0005–0.202 | >160 | Kasprzyk-Hordern *et al*. (2008) |  |  |  | 175800 (*Monia macrocopa*, 48h^1^; Lee *et al*., 2015) |
|  | 0.0021–0.0496 | 132 | Burns *et al*. (2018) |  |  |  |  |
|  | 0.0514–0.0702 | 6 | Wilkinson *et al*. (2019) |  |  |  |  |
| Citalopram | 0.0031–0.0714 | 132 | Burns *et al*. (2018) |  |  |  | 3900 (*Ceriodaphnia dubia*; 48 h^1^) |
|  | 0.0036–0.139 | 6 | Wilkinson *et al*. (2019) |  |  |  |  |
|  | 0.00618–0.00639 | 3 | Sims *et al*. (2023) |  |  |  |  |
|  | 0.006–0.547 | 358 | Egli *et al*. (2023) |  |  |  |  |
|  | 0.014–0.037 | 24 | Burns *et al*. (2017) |  |  |  |  |
| Ciprofloxacin | <0.001–0.005 | 945 | Buglife (2021) |  | 2500 (*Ceriodaphnia dubia*; 21 days^3^) | 12500 (*Ceriodaphnia dubia*; 21 days^3^) | 4000 (*Ceriodaphnia dubia*; 21 days^1^) |
| Celecoxib | 0.005–0.017 | 9 | Egli *et al*. (2023) |  |  | 480 (*Ceriodaphnia dubia*; 7 days^3^) |  |
| Clozapine | 0.005–0.046 | 141 | Egli *et al*. (2023) |  |  |  |  |
| Clofibric acid | <0.0003–0.164 | >160 | Kasprzyk-Hordern *et al*. (2008) |  |  | 2560 (*Ceriodaphnia dubia*; 7 days^3^) | 740 (*Brachionus calyciflorus*; 48 h^3^) |
|  | <0.05 | 2 | Hilton & Thomas (2003) |  |  |  |  |
| N-desmethyl clarithromycin | 0.03198–0.09531 | 3 | Sims *et al*. (2023) |  |  |  |  |
| Clarithromycin | 0.0002–0.158 | 124 | Niemi *et al*. (2022) |  |  |  | 35460 (*Brachionus calyciflorus*; 24 h^1^) |
|  | 1.1 | 2025 | Spurgeon *et al*. (2021) |  |  |  |  |
|  | <0.0005–1.250 | 961 | Buglife (2021) |  |  |  |  |
|  | 0.00407–0.05101 | 3 | Sims *et al*. (2023) |  |  |  |  |
|  | 0.008–0.713 | 87 | Egli *et al*. (2023) |  |  |  |  |
|  | 0.102 |  | Proctor *et al*. (2019) |  |  |  |  |
| N-desmethyl clindamycin | 0.00007–0.00129 | 3 | Sims *et al*. (2023) |  |  |  |  |
| Clindamycin | 0.00031–0.01585 | 3 | Sims *et al*. (2023) |  |  |  |  |
| Clopidogrel | 0.003–0.068 | 251 | Egli *et al*. (2023) |  |  |  |  |
| Clotrimazole | 0.006–0.034 | 54 | Roberts & Thomas (2006) |  |  |  |  |
| Cocaine (illicit drug) | <0.0003–0.007 | >160 | Kasprzyk-Hordern *et al*. (2008) |  |  |  |  |
|  | 0.004–0.113 | 288 | Egli *et al*. (2023) |  |  |  |  |
|  | 0.00085–0.00111 | 3 | Sims *et al*. (2023) |  |  |  |  |
|  | 0.0049 |  | Proctor *et al*. (2019) |  |  |  |  |
| Codeine | 0.0012–0.101 | 132 | Burns *et al*. (2018) |  |  |  |  |
|  | <0.0015–0.815 | >160 | Kasprzyk-Hordern *et al*. (2008) |  |  |  |  |
|  | 0.02951–0.02991 | 3 |  |  |  |  |  |
|  | 0.0856 |  | Proctor *et al*. (2019) |  |  |  |  |
|  | 0.02105–0.02263 | 3 | Sims *et al*. (2023) |  |  |  |  |
|  | 0.0007–31 | 14 | Ramage *et al*. (2019) |  |  |  |  |
| Crotamiton | 8.8 | 5195 | Spurgeon *et al*. (2021) |  |  |  |  |
| Desmethylcitalopram | 0.012 |  | Proctor *et al*. (2019) |  |  |  |  |
|  | 0.00431–0.00432 | 3 | Sims *et al*. (2023) |  |  |  |  |
| Desmethylvenlafaxine | 0.2292 |  | Proctor *et al*. (2019) |  |  |  |  |
|  | 0.016–0.085 | 24 | Burns *et al*. (2017) |  |  |  |  |
| Desmethyl-diltiazem | 0.044–0.048 | 24 | Burns *et al*. (2017) |  |  |  |  |
| Desvenlafaxine | 0.0046–0.268 | 132 | Burns *et al*. (2018) |  |  |  |  |
|  | 0.319–1.620 | 6 | Wilkinson *et al*. (2019) |  |  |  |  |
| Dextromethorphan | 0.006–0.0067 | 24 | Burns *et al*. (2017) |  |  |  |  |
| Dextropropoxyphene | <0.02–0.682 | 30 | Ashton *et al*. (2004) |  |  |  | 6015.0192 (*Brachionus calyciflorus*; 24 h^1^) |
|  | <0.008–0.098 | 54 | Roberts & Thomas (2006) |  |  |  |  |
|  | <0.02 | 2 | Hilton & Thomas (2003) |  |  |  |  |
| Dihydrocodeine | 0.0221 |  | Proctor *et al*. (2019) |  |  |  |  |
|  | 0.01098–0.01126 | 3 | Sims *et al*. (2023) |  |  |  |  |
| Diazepam | 0.0016–0.0018 | 132 | Burns *et al*. (2018) |  |  |  |  |
|  | 0.0062 |  | Proctor *et al*. (2019) |  |  |  |  |
|  | 0.001–0.0013 | 24 | Burns *et al*. (2017) |  |  |  |  |
| Diclofenac | <0.0005–0.261 | >160 | Kasprzyk-Hordern *et al*. (2008) |  |  | 2000 (*Ceriodaphnia dubia*; 7 days^3^)  940 (*Daphnia magna*; 21 days^2^; Du *et al*., 2016) | 0.041 (*Dreissena polymorpha***;** 162 days^3^) |
|  | 0.025–2.991 | 125 | Kay *et al*. (2017) |  |  |  |  |
|  | <0.002–0.568 | 30 | Ashton *et al*. (2004) |  |  |  |  |
|  | 0.0002–0.324 | 124 | Niemi *et al*. (2022) |  |  |  |  |
|  | 0.543 |  | Zhou & Broodbank (2014) |  |  |  |  |
|  | 0.76 | 2360 | Spurgeon *et al*. (2021) |  |  |  |  |
|  | <0.0001–0.372 | 961 | Buglife (2021) |  |  |  |  |
|  | 0.008–0.527 | 351 | Egli *et al*. (2023) |  |  |  |  |
|  | 0.0882 |  | Proctor *et al*. (2019) |  |  |  |  |
|  | 0.0032–0.0147 |  | Zhang & Zhou (2007) |  |  |  |  |
|  | <0.02–0.091 | 2 | Hilton & Thomas (2003) |  |  |  |  |
| Digoxigenin | <0.03 | >160 | Kasprzyk-Hordern *et al*. (2008) |  |  |  |  |
| Digoxin | <0.03 | >160 | Kasprzyk-Hordern *et al*. (2008) |  |  |  |  |
| Diltiazem | <0.001–0.065 | >160 | Kasprzyk-Hordern *et al*. (2008) |  |  |  |  |
|  | 0.0041–0.0487 | 132 | Burns *et al*. (2018) |  |  |  |  |
|  | 0.0056–0.0367 | 6 | Wilkinson *et al*. (2019) |  |  |  |  |
|  | 0.00165–0.00167 | 3 |  |  |  |  |  |
|  | 0.0044 |  | Proctor *et al*. (2019) |  |  |  |  |
| Diphenhydramine | 0.0016–0.0127 | 132 | Burns *et al*. (2018) |  |  | 3.44 (*Daphnia magna*; 10 days^3^) | 0.37 (*Daphnia magna*; 48 h^1^) |
|  | 0.005–0.151 | 240 | Egli *et al*. (2023) |  |  |  |  |
|  | 0.0056–0.006 | 24 | Burns *et al*. (2017) |  |  |  |  |
| EDDP | 0.01 |  | Proctor *et al*. (2019) |  |  |  |  |
|  | 0.00244–0.00257 | 3 | Sims *et al*. (2023) |  |  |  |  |
| Ephedrine/ pseudoephedrine | 0.0256 |  | Proctor *et al*. (2019) |  |  |  |  |
| N-desmethyl erythromycin | 0.00021–0.00088 | 3 | Sims *et al*. (2023) |  |  |  |  |
| Erythromycin | <0.0005–0.351 | >160 | Kasprzyk-Hordern *et al*. (2008) |  |  |  | 27530 (*Brachionus calyciflorus*; 24 h^1^) |
|  | <0.01–1.022 | 30 | Ashton *et al*. (2004) |  |  |  |  |
|  | 0.015–0.263 | 132 | Burns *et al*. (2018) |  |  |  |  |
|  | 0.034–1.378 | 125 | Kay *et al*. (2017) |  |  |  |  |
|  | <0.005–0.78 | 961 | Buglife (2021) |  |  |  |  |
|  | <0.004–0.07 | 54 | Roberts & Thomas (2006) |  |  |  |  |
|  | 0.00208–0.02385 | 3 | Sims *et al*. (2023) |  |  |  |  |
|  | 0.057–1.0 | 2 | Hilton & Thomas (2003) |  |  |  |  |
|  | 2.1482 |  | Proctor *et al*. (2019) |  |  |  |  |
|  | 0.17–0.18 | 24 | Burns *et al*. (2017) |  |  |  |  |
| Ethylparaben | <0.0005–0.015 | >160 | Kasprzyk-Hordern *et al*. (2008) |  |  |  | 217800 (*Caenorhabditis elegans*; 72h^1^; Nagar *et al*., 2020) |
| Furosemide | <0.006–0.630 | >160 | Kasprzyk-Hordern *et al*. (2008) |  |  |  |  |
|  | 1.8 | 654 | Spurgeon *et al*. (2021) |  |  |  |  |
| Fexofenadine | 0.0172–1.144 | 132 | Burns *et al*. (2018) |  |  |  |  |
|  | 0.575–1.640 | 6 | Wilkinson *et al*. (2019) |  |  |  |  |
|  | 0.2091 |  | Proctor *et al*. (2019) |  |  |  |  |
|  | 0.04989–0.05332 | 3 | Sims *et al*. (2023) |  |  |  |  |
|  | 0.018–0.13 | 24 | Burns *et al*. (2017) |  |  |  |  |
| Flucloxacillin | 0.00453–0.03097 | 3 | Sims *et al*. (2023) |  |  |  |  |
| Fluconazole | <0.0109–0.102 | 8 | Wilkinson *et al*. (2019) |  |  |  |  |
| Fluoxetine | 0.0081–0.0979 | 60 | Niemi *et al*. (2022) |  | 13 (*Potamopyrgus antipodarum*; 42 days^3^) | 0.02 (*Dreissena polymorpha*; 6 days^3^) | 510 (*Ceriodaphnia dubia*; 48 h^1^)  330 (*Daphnia magna*, 6 days^1^; Hansen *et al*., 2008) |
|  | <0.0001–0.0869 | 961 | Buglife (2021) |  |  |  |  |
|  | 0.005–0.045 | 124 | Egli *et al*. (2023) |  |  |  |  |
|  | 0.0012 |  | Proctor *et al*. (2019) |  |  |  |  |
| Gabapentin | 0.00174–1.445 | 132 | Burns *et al*. (2018) |  |  |  |  |
|  | <0.0006–1.887 | >160 | Kasprzyk-Hordern *et al*. (2008) |  |  |  |  |
|  | 0.0126–0.946 | 8 | Wilkinson *et al*. (2019) |  |  |  |  |
| Gliclazide | 0.0473 |  | Proctor *et al*. (2019) |  |  |  |  |
|  | 0.0015–0.00185 | 3 | Sims *et al*. (2023) |  |  |  |  |
| Glyburide | 0.0031 | 24 | Burns *et al*. (2017) |  |  |  |  |
| Hydrocodone | 0.0052–0.0918 | 132 | Burns *et al*. (2018) |  |  |  |  |
|  | 0.0041–0.0126 | 6 | Wilkinson *et al*. (2019) |  |  |  |  |
|  | 0.034–0.039 | 24 | Burns *et al*. (2017) |  |  |  |  |
| Hydrochlorothiazide | 0.013–0.04 | 5 | Egli *et al*. (2023) |  |  |  |  |
| Ibuprofen | <0.0003–0.091 | >160 | Kasprzyk-Hordern *et al*. (2008) | 2430 (*Planorbis carinatus*; 21 days^3^) | 24000 (*Daphnia magna*; 20 days^3^) | 1230 (*Daphnia magna*; 21 days^3^) | 5360 (*Planorbis carinatus*; 7 days^3^) |
|  | <0.02–27.256 | 30 | Ashton *et al*. (2004) |  |  |  |  |
|  | 0.1–4.838 | 125 | Kay *et al*. (2017) |  |  |  |  |
|  | 0.0001–0.697 | 124 | Niemi *et al*. (2022) |  |  |  |  |
|  | 1.1 | 1585 | Spurgeon *et al*. (2021) |  |  |  |  |
|  | <0.0025–6.6 | 968 | Buglife (2021) |  |  |  |  |
|  | 0.144–2.37 | 54 | Roberts & Thomas (2006) |  |  |  |  |
|  | 0.0605 |  | Proctor *et al*. (2019) |  |  |  |  |
|  | 0.05956–0.06708 | 3 | Sims *et al*. (2023) |  |  |  |  |
|  | <0.02 | 2 | Hilton & Thomas (2003) |  |  |  |  |
| Indomethacine | 0.0006–0.0018 |  | Zhang & Zhou (2007) |  |  |  |  |
| Imatinib | 0.0383 |  | Proctor *et al*. (2019) |  |  |  |  |
| Indomethacin | 0.006–0.028 |  | Zhou & Broodbank (2014) |  |  |  |  |
| Iohexol | 1.4 | 422 | Spurgeon *et al*. (2021) |  |  |  |  |
| Irbesartan | 0.1114 |  | Proctor *et al*. (2019) |  |  |  |  |
|  | 0.01821–0.01848 | 3 | Sims *et al*. (2023) |  |  |  |  |
| Levocabastine | 0.006–0.047 | 4 | Egli *et al*. (2023) |  |  |  |  |
| Lisinopril | 0.01393–0.01398 | 3 | Sims *et al*. (2023) |  |  |  |  |
| Lidocaine | 0.0017–0.0404 | 132 | Burns *et al*. (2018) |  |  |  |  |
|  | <0.00266–0.258 | 8 | Wilkinson *et al*. (2019) |  |  |  |  |
|  | 0.005–1.432 | 356 | Egli *et al*. (2023) |  |  |  |  |
|  | 0.0089–0.0096 | 24 | Burns *et al*. (2017) |  |  |  |  |
| Lofepramine | <0.01 | 2 | Hilton & Thomas (2003) |  |  |  |  |
| Loratadine | 0.00646–0.0083 | 132 | Burns *et al*. (2018) |  |  |  |  |
|  | <0.0206 | 8 | Wilkinson *et al*. (2019) |  |  |  |  |
|  | 0.0015–0.0085 | 24 | Burns *et al*. (2017) |  |  |  |  |
| Lamotrigine | 1.6 | 2612 | Spurgeon *et al*. (2021) |  |  |  |  |
| Ketoconazole | 0.0465 |  | Proctor *et al*. (2019) |  |  |  | 1500 (*Dapnia magna*; 48 h^2^ ; Haeba *et al*., 2008) |
| Ketoprofen | <0.0005–0.012 | >160 | Kasprzyk-Hordern *et al*. (2008) |  |  |  |  |
|  | 0.05752–0.05801 | 3 | Sims *et al*. (2023) |  |  |  |  |
| Ketamine (illicit drug) | 0.005–0.140 | 355 | Egli *et al*. (2023) |  |  |  | 30930 (*Daphnia magna*; 48h^1^; Li *et al*., 2017) |
|  | 0.01441–0.01458 | 3 | Sims *et al*. (2023) |  |  |  |  |
|  | 0.0118 |  | Proctor *et al*. (2019) |  |  |  |  |
| Mebeverine | 0.003–0.011 |  | Zhou & Broodbank (2014) |  |  |  |  |
| MDA | 0.010 |  | Proctor *et al*. (2019) |  |  |  |  |
| Mefenamic acid | 0.028–0.176 |  | Zhou & Broodbank (2014) |  |  |  |  |
|  | <0.0003–0.033 | >160 | Kasprzyk-Hordern *et al*. (2008) |  |  |  |  |
|  | <0.005–0.366 | 30 | Ashton *et al*. (2004) |  |  |  |  |
|  | 0.006–0.097 | 125 | Kay *et al*. (2017) |  |  |  |  |
|  | 0.006–0.166 | 102 | Egli *et al*. (2023) |  |  |  |  |
|  | <0.05–0.065 | 2 | Hilton & Thomas (2003) |  |  |  |  |
| Memantine | 0.004–0.036 | 260 | Egli *et al*. (2023) |  |  |  |  |
| Methamphetamine | 0.005–0.089 | 116 | Egli *et al*. (2023) |  |  |  |  |
| Methylparaben | <0.0003–0.4 | >160 | Kasprzyk-Hordern *et al*. (2008) |  |  |  |  |
|  | 0.0158–0.26 | 3 | Sims *et al*. (2023) |  |  |  |  |
|  | 0.007 |  | Proctor *et al*. (2019) |  |  |  |  |
| Methylphenidate | 0.004–0.083 | 13 | Egli *et al*. (2023) |  |  |  |  |
| Metformin | 0.0452–2.595 | 132 | Burns *et al*. (2018) |  |  |  |  |
|  | <0.0184–0.923 | 8 | Wilkinson *et al*. (2019) |  |  |  |  |
|  | 3.6071 |  | Proctor *et al*. (2019) |  |  |  |  |
|  | 1.34263–1.35884 | 3 | Sims *et al*. (2023) |  |  |  |  |
|  | 0.63–1.3 | 24 | Burns *et al*. (2017) |  |  |  |  |
| Methedrone (illicit drug) | 0.007–0.018 | 2 | Egli *et al*. (2023) |  |  |  |  |
| Methadone | 0.003 |  | Proctor *et al*. (2019) |  |  |  |  |
|  | 0.00011 | 3 | Sims *et al*. (2023) |  |  |  |  |
| Methocarbamol | 0.0087–0.01 | 24 | Burns *et al*. (2017) |  |  |  |  |
| Metoprolol | <0.0005–0.012 | >160 | Kasprzyk-Hordern *et al*. (2008) | 3200 (*Daphnia magna*; 9 days^3^) |  | 6150 (*Daphnia magna*; 9 days^3^) |  |
|  | 0.004–0.016 | 112 | Egli *et al*. (2023) |  |  |  |  |
| Metronidazole | <0.0015–0.024 | >160 | Kasprzyk-Hordern *et al*. (2008) |  |  |  |  |
|  | 0.0101 |  | Proctor *et al*. (2019) |  |  |  |  |
| Mirtazapine | 0.0042 |  | Proctor *et al*. (2019) |  |  |  |  |
| Morphine | 0.018–0.132 | 14 | Egli *et al*. (2023) |  |  |  |  |
|  | 0.019–0.2 | 24 | Burns *et al*. (2017) |  |  |  |  |
| Naproxen | <0.0003–0.139 | >160 | Kasprzyk-Hordern *et al*. (2008) |  |  |  |  |
|  | 0.08009–0.08174 | 3 | Sims *et al*. (2023) |  |  |  |  |
|  | 0.237 |  | Proctor *et al*. (2019) |  |  |  |  |
| N-Desmethyltramadol | 0.2553 |  | Proctor *et al*. (2019) |  |  |  |  |
|  | 0.0021–0.00385 | 3 | Sims *et al*. (2023) |  |  |  |  |
| Nicotine | 0.009–0.327 | 313 | Egli *et al*. (2023) | 70 (*Daphnia pulex*; 16 days^3^) |  | 180 (*Daphnia pulex*; 16 days^3^) | 219016.71 (*Brachionus calyciflorus*; 24 h^3^)  789.23 (*Daphnia magna*; 48h^1^; Oropesa *et al*., 2017) |
|  | 0.0406–0.041 | 3 | Sims *et al*. (2023) |  |  |  |  |
|  | 0.0359 |  | Proctor *et al*. (2019) |  |  |  |  |
| Norethisterone | 0.0074–0.0077 | 132 | Burns *et al*. (2018) |  |  |  |  |
| Nortriptyline | 0.004–0.015 | 34 | Egli *et al*. (2023) |  |  |  |  |
|  | 0.0078 |  | Proctor *et al*. (2019) |  |  |  |  |
| Norketamine | 0.00136–0.00145 | 3 | Sims *et al*. (2023) |  |  |  | 25350 (*Daphnia magna*; 48 h^1^; Li *et al*., 2017) |
| Norcodeine | 0.01011–0.01225 | 3 | Sims *et al*. (2023) |  |  |  |  |
| O-Desmethyltramadol | 0.297 |  | Proctor *et al*. (2019) |  |  |  |  |
|  | 0.02393–0.02404 | 3 | Sims *et al*. (2023) |  |  |  |  |
| Oestrone (E1) | <0.0003–0.0656 | 1036 | Buglife (2021) |  |  |  |  |
| Ofloxacin | 0.0715 |  | Proctor *et al*. (2019) |  |  |  | 29880 (*Brachionus calyciflorus*; 24 h^1^) |
| Orphenadrine | 0.006–0.079 | 46 | Egli *et al*. (2023) |  |  |  |  |
| Oseltamivir | 0.0088 | 132 | Burns *et al*. (2018) |  |  |  |  |
|  | 0.0036 | 24 | Burns *et al*. (2017) |  |  |  |  |
| Oxazepam | 0.006–0.028 | 43 | Egli *et al*. (2023) |  |  |  |  |
|  | 0.002 |  | Proctor *et al*. (2019) |  |  |  |  |
| Oxycodone | 0.005–0.011 | 20 | Egli *et al*. (2023) |  |  |  |  |
| Paracetamol | 0.0143–9.822 | 132 | Burns *et al*. (2018) |  |  | 950 (*Moina macrocopa*; 7 days^3^)  80 (*Daphnia longispina*; 21 days^2^; Nunes *et al*., 2014) | 6440 (*Daphnia magna*; 48 h^3^) |
|  | <0.0015–2.382 | >160 | Kasprzyk-Hordern *et al*. (2008) |  |  |  |  |
|  | 0.034–0.658 | 64 | Niemi *et al*. (2022) |  |  |  |  |
|  | 1–1100 | 14 | Ramage *et al*. (2019) |  |  |  |  |
|  | 0.26–1.0 | 24 | Burns *et al*. (2017) |  |  |  |  |
|  | <0.05 | 2 | Hilton & Thomas (2003) |  |  |  |  |
|  | 0.193 |  | Proctor *et al*. (2019) |  |  |  |  |
|  | 0.09280–0.10849 | 3 | Sims *et al*. (2023) |  |  |  |  |
| p-Benzylphenol | <0.015–0.058 | >160 | Kasprzyk-Hordern *et al*. (2008) |  |  |  |  |
| Pirenzepine | 0.005–0.013 | 32 | Egli *et al*. (2023) |  |  |  |  |
| Pravastatin | <0.06 | >160 | Kasprzyk-Hordern *et al*. (2008) |  |  |  |  |
| Propranolol | <0.0005–0.091 | >160 | Kasprzyk-Hordern *et al*. (2008) | 440 (*Daphnia magna*; 9 days^3^) |  | 110 (*Daphnia magna*; 9 days^3^) | 2593.482 (*Brachionus calyciflorus*; 24 h^1^)  1600 (*Daphnia magna*; 48h^1^; Huggett *et al*., 2002) |
|  | <0.01–0.215 | 30 | Ashton *et al*. (2004) |  |  |  |  |
|  | 0.0076–0.0649 | 132 | Burns *et al*. (2018) |  |  |  |  |
|  | 0.003–0.165 | 125 | Kay *et al*. (2017) |  |  |  |  |
|  | 0.008–0.035 |  | Zhou & Broodbank (2014) |  |  |  |  |
|  | <0.0001–0.34 | 961 | Buglife (2021) |  |  |  |  |
|  | 0.035–0.107 | 54 | Roberts & Thomas (2006) |  |  |  |  |
|  | <0.01486–0.0857 | 8 | Wilkinson *et al*. (2019) |  |  |  |  |
|  | 0.005–0.161 | 337 | Egli *et al*. (2023) |  |  |  |  |
|  | 0.0039–0.0285 |  | Zhang & Zhou (2007) |  |  |  |  |
|  | 0.0206 |  | Proctor *et al*. (2019) |  |  |  |  |
|  | 0.018–0.027 | 24 | Burns *et al*. (2017) |  |  |  |  |
|  | <0.01–0.037 | 2 | Hilton & Thomas (2003) |  |  |  |  |
| Propylparaben | <0.0002–0.022 | >160 | Kasprzyk-Hordern *et al*. (2008) |  |  | 20 (*Aedes aegypti*; 4.4167 days^3^) | 20 (*Aedes aegypti*; 12 h^3^) |
|  | 0.0037 |  | Proctor *et al*. (2019) |  |  |  |  |
| Pseudoephedrine | 0.008–0.0085 | 24 | Burns *et al*. (2017) |  |  |  |  |
| Quetiapine | 0.00003 | 3 | Sims *et al*. (2023) |  |  |  |  |
| Quinine | 0.023–0.041 | 24 | Burns *et al*. (2017) |  |  |  |  |
| Raloxifene | 0.0072 | 132 | Burns *et al*. (2018) |  |  |  |  |
| Ranitidine | <0.003–0.073 | >160 | Kasprzyk-Hordern *et al*. (2008) |  |  |  |  |
|  | 0.0066–0.074 | 132 | Burns *et al*. (2018) |  |  |  |  |
|  | 0.205–0.315 | 6 | Wilkinson *et al*. (2019) |  |  |  |  |
|  | 0.1482 |  | Proctor *et al*. (2019) |  |  |  |  |
|  | 0.072–0.18 | 24 | Burns *et al*. (2017) |  |  |  |  |
| Rifampicin | 0.0151–0.09545 | 3 | Sims *et al*. (2023) |  |  |  |  |
| Risperidone | 0.004–0.045 | 29 | Egli *et al*. (2023) |  |  |  |  |
|  | 0.0012 |  | Proctor *et al*. (2019) |  |  |  |  |
| Rizatriptan | 0.02 | 1 | Egli *et al*. (2023) |  |  |  |  |
| Salbutamol | <0.0005–0.008 | >160 | Kasprzyk-Hordern *et al*. (2008) |  |  |  |  |
|  | <0.0103 | 8 | Wilkinson *et al*. (2019) |  |  |  |  |
|  | 0.004–0.021 | 190 | Egli *et al*. (2023) |  |  |  |  |
| Salicylic acid | <0.0003–0.302 | >160 | Kasprzyk-Hordern *et al*. (2008) | 1800 (*Daphnia magna*; 21 days^3^) |  | 1800 (*Daphnia magna*; 21 days^3^) | 141425.6785 (*Brachionus calyciflorus*; 24 h^1^) |
|  | 0.008–3.326 | 337 | Egli *et al*. (2023) |  |  |  |  |
| Sertraline | 0.0212 | 132 | Burns *et al*. (2018) |  |  | 66 (*Daphnia magna*; 21 days^3^; Minagh *et al*., 2009) | 120 (*Ceriodaphnia dubia*; 48 h^3^)  100 (*Daphnia magna*; 21 days^3^; Minagh *et al*., 2009) |
|  | 0.004–0.066 | 56 | Egli *et al*. (2023) |  |  |  |  |
| Sildenafil | 0.0022 |  | Proctor *et al*. (2019) |  |  |  |  |
| Simvastatin | <0.05 | >160 | Kasprzyk-Hordern *et al*. (2008) |  |  |  |  |
| Sitagliptin | 0.0107–0.121 | 132 | Burns *et al*. (2018) |  |  |  |  |
|  | 0.0285–0.113 | 6 | Wilkinson *et al*. (2019) |  |  |  |  |
|  | 0.1425 |  | Proctor *et al*. (2019) |  |  |  |  |
|  | 0.02–0.036 | 24 | Burns *et al*. (2017) |  |  |  |  |
| Sulfamethoxazole | 0.0011 |  | Zhou & Broodbank (2014) | 370 (*Daphnia magna*; 21 days^3^) |  | 120 (*Daphnia magna*; 21 days^3^) | 26270 (*Brachionus calyciflorus*; 24 h^1^) |
|  | <0.0005–0.004 | >160 | Kasprzyk-Hordern *et al*. (2008) |  |  |  |  |
|  | <0.05–0.239 | 30 | Ashton *et al*. (2004) |  |  |  |  |
|  | 0.0102–0.0275 | 132 | Burns *et al*. (2018) |  |  |  |  |
|  | 0.188–0.41 | 6 | Wilkinson *et al*. (2019) |  |  |  |  |
|  | 0.00394–0.03267 | 3 | Sims *et al*. (2023) |  |  |  |  |
|  | 0.006–0.09 | 197 | Egli *et al*. (2023) |  |  |  |  |
|  | <0.05 | 2 | Hilton & Thomas (2003) |  |  |  |  |
|  | 0.0046 |  | Zhang & Zhou (2007) |  |  |  |  |
|  | 0.0332 |  | Proctor *et al*. (2019) |  |  |  |  |
| Sulfapyridine | <0.002–0.142 | >160 | Kasprzyk-Hordern *et al*. (2008) |  |  |  |  |
|  | 0.1287 |  | Proctor *et al*. (2019) |  |  |  |  |
|  | 0.00172–0.07474 | 3 | Sims *et al*. (2023) |  |  |  |  |
|  | 0.006–0.366 | 352 | Egli *et al*. (2023) |  |  |  |  |
| N-acetyl sulfapyridine | 0.00193–0.00981 | 3 | Sims *et al*. (2023) |  |  |  |  |
| Sulfasalazine | <0.0015–0.168 | >160 | Kasprzyk-Hordern *et al*. (2008) |  |  |  |  |
|  | 0.0536 |  | Proctor *et al*. (2019) |  |  |  |  |
| Tamoxifen | <0.01 | 30 | Ashton *et al*. (2004) |  |  |  |  |
|  | 0.002–0.008 |  | Zhou & Broodbank (2014) |  |  |  |  |
|  | 0.027–0.212 | 54 | Roberts & Thomas (2006) |  |  |  |  |
|  | <0.01 | 2 | Hilton & Thomas (2003) |  |  |  |  |
| Tamsulosin | 0.004–0.008 | 14 | Egli *et al*. (2023) |  |  |  |  |
| Tacrine | 0.016–0.024 | 2 | Egli *et al*. (2023) |  |  |  |  |
| Telmisartan | 0.082 | 826 | Spurgeon *et al*. (2021) |  |  |  |  |
| Temazepam | 0.0044–0.0382 | 132 | Burns *et al*. (2018) |  |  |  |  |
|  | <0.0353 | 8 | Wilkinson *et al*. (2019) |  |  |  |  |
|  | 0.005–0.058 | 269 | Egli *et al*. (2023) |  |  |  |  |
| Thioridazine | 0.006–0.022 |  | Zhou & Broodbank (2014) |  |  |  | 325.62544 (*Brachionus calyciflorus*; 24 h^1^) |
| Tramadol | <0.030–7.731 | >160 | Kasprzyk-Hordern *et al*. (2008) |  |  |  |  |
|  | 0.0039–0.650 | 132 | Burns *et al*. (2018) |  |  |  |  |
|  | <0.0134–0.419 | 8 | Wilkinson *et al*. (2019) |  |  |  |  |
|  | 0.004–0.422 | 381 | Egli *et al*. (2023) |  |  |  |  |
|  | 0.3217 |  | Proctor *et al*. (2019) |  |  |  |  |
|  | 0.049–0.077 | 24 | Burns *et al*. (2017) |  |  |  |  |
| Triclosan | <0.0005–0.095 | >160 | Kasprzyk-Hordern *et al*. (2008) | 80 (*Chironomus tentans*; 10 days^4^) |  | 72.38635 (*Daphnia magna*; 13 days^3^) | 20 (*Chironomus tentans*; 10 days^5^) |
|  | 58 | 874 | Spurgeon *et al*. (2021) |  |  |  |  |
| Triamterene | <0.00478–0.0711 | 8 | Wilkinson *et al*. (2019) |  |  |  |  |
|  | 0.0042 | 24 | Burns *et al*. (2017) |  |  |  |  |
| Trimethoprim | 0.0001–0.505 | 124 | Niemi *et al*. (2022) | 25000 (*Daphnia magna*; 21 days^3^) |  | 2250 (*Daphnia magna*; 21 days^6^) |  |
|  | 0.002–0.076 | 132 | Burns *et al*. (2018) |  |  |  |  |
|  | <0.0015–0.183 | >160 | Kasprzyk-Hordern *et al*. (2008) |  |  |  |  |
|  | <0.01–1.288 | 30 | Ashton *et al*. (2004) |  |  |  |  |
|  | 0.02 |  | Environment Agency (2006) |  |  |  |  |
|  | 0.004–0.019 | 54 | Roberts & Thomas (2006) |  |  |  |  |
|  | 0.0127–0.0397 | 6 | Wilkinson *et al*. (2019) |  |  |  |  |
|  | 0.005–0.328 | 356 | Egli *et al*. (2023) |  |  |  |  |
|  | 0.0259 |  | Proctor *et al*. (2019) |  |  |  |  |
|  | <0.01–0.039 | 2 | Hilton & Thomas (2003) |  |  |  |  |
|  | 0.00169–0.01004 | 3 | Sims *et al*. (2023) |  |  |  |  |
|  | 0.022–0.036 | 24 | Burns *et al*. (2017) |  |  |  |  |
| Valsartan | <0.0005–0.144 | >160 | Kasprzyk-Hordern *et al*. (2008) |  |  |  |  |
|  | 0.007–0.046 | 21 | Egli *et al*. (2023) |  |  |  |  |
| Valstarten | 0.05804–0.05896 | 3 | Sims *et al*. (2023) |  |  |  |  |
| Venlafaxine | 0.0023–0.102 | 132 | Burns *et al*. (2018) |  |  |  | 141280 (*Daphnia magna*, 48 h^2^; Minguez *et al*., 2014) |
|  | <0.00237–0.681 | 8 | Wilkinson *et al*. (2019) |  |  |  |  |
|  | 0.005–0.386 | 370 | Egli *et al*. (2023) |  |  |  |  |
|  | 0.02206–0.02255 | 3 | Sims *et al*. (2023) |  |  |  |  |
|  | 0.0855 |  | Proctor *et al*. (2019) |  |  |  |  |
|  | 0.012–0.015 | 24 | Burns *et al*. (2017) |  |  |  |  |
| Verapamil | 0.004-0.036 | 56 | Egli *et al*. (2023) |  |  |  | 10901.76954 (*Brachionus calyciflorus*; 24 h^1^) |

^1^LC_50_, lethal concentration for 50%; ^2^EC_50_, effect concentration for 50%; ^3^LOEC, lowest observed effect concentration; ^4^EC_10_, effect concentration for 10%; ^5^LC_10,_ lethal concentration for 10%; ^6^EC_20_, effect concentration of 20%; ^7^NOEC, no observed effect concentration. Blue-highlighted chemicals are Personal Care Product chemicals. Orange-highlighted concentrations indicate those identified through a wider literature search using *Google Scholar* and *Web of Science*. All other concentration data were obtained from the *ECOTOX* database.

**Table S5.** Concentrations of veterinary pharmaceuticals recorded across British rivers (wastewater treatment effluents) and lowest effect concentrations (ECs) for British freshwater invertebrates based on laboratory tests.

| **Chemical** | **Field concentrations (µg/l)** | **Number of samples** | **References** | **EC for growth (µg/l)** | **EC for development (µg/l)** | **EC for reproduction (µg/l)** | **EC for mortality (µg/l)** |
| --- | --- | --- | --- | --- | --- | --- | --- |
| Clopidol | 0.077 | 2551 | Spurgeon *et al*. (2021) |  |  |  |  |
| Chlorfenvinphos (surface fresh water) | 0.001–0.242 | 3634 | Boxall *et al*. (2002) |  |  |  | 0.4 (*Ceriodaphnia dubia*; 48 h^1^) |
| Flumethrin (surface fresh water) | 0.001–2.19 | 2043 | Boxall *et al*. (2002) |  |  |  |  |
| Propetamphos (surface fresh water) | 0.001–11738 | 3763 | Boxall *et al*. (2002) |  |  |  |  |
| Levamisole | 0.006–0.081 | 145 | Egli *et al*. (2023) |  |  |  | 64000 (*Daphnia magana*; 28 h^1^; Yoshimura & Endoh, 2005) |
| Lincomycin | 21.1 |  | Environment Agency (2006) |  |  |  | 24940 (Brachionus calyciflorus; 24 h^1^) |
|  | 0.004–0.009 | 3 | Egli *et al*. (2023) |  |  |  |  |
| Oxytetracycline | 4.49 |  | Environment Agency (2006) |  |  |  | 34210 (*Brachionus calyciflorus*; 24 h^1^)  4600 (*Daphnia magna*; 48h^1^; Wollenberger *et al*., 2000) |
| Sulfadiazine | 4.13 |  | Environment Agency (2006) |  |  | 13700 (*Daphnia magan*; 21 day^2^; Wollenberger *et al*., 2000) |  |
| Ceftiofur | 0.0312 |  | Proctor *et al.* (2019) |  |  |  |  |

^1^LC_50_, lethal concentration for 50%; ^2^EC_50_, effect concentration for 50%). Concentrations in an orange font indicate those identified through a wider literature search using *Google Scholar* and *Web of Science*. All other concentration data were obtained from the *ECOTOX* database.

**Table S6.** Concentrations of persistent organic pollutants (POPs) recorded across British rivers (wastewater treatment effluents) and lowest effect concentrations (ECs) for British freshwater invertebrates based on laboratory tests.

| **Chemical** | **Field concentrations (µg/l)** | **Number of samples** | **References** | **EC for growth (µg/l)** | **EC for development (µg/l)** | **EC for reproduction (µg/l)** | **EC for mortality (µg/l)** |
| --- | --- | --- | --- | --- | --- | --- | --- |
| PBDE 28 | 0.00002–0.00026 | 750 | Chemical Investigations Programme Gardner *et al*. (2022) |  |  |  |  |
| PBDE 47 | 0.00025–0.0025 | 750 | Chemical Investigations Programme Gardner *et al*. (2022) |  |  | 45  (*Daphnia magna*; 21 days^1^) | 100  (*Daphnia magna*; 48 h^1^) |
| PBDE 99 | 0.00025–0.0025 | 750 | Chemical Investigations Programme Gardner *et al*. (2022) |  |  |  | 25  (*Daphnia magna*; 48 h^1^) |
| PBDE 100 | 0.00006–0.00073 | 750 | Chemical Investigations Programme Gardner *et al*. (2022) |  |  |  |  |
| PBDE 153 | 0.00004–0.00036 | 750 | Chemical Investigations Programme Gardner *et al*. (2022) |  |  |  | 14.6  (*Daphnia magna*; 21 days^1^) |
| PBDE 154 | 0.00003–0.0008 | 750 | Chemical Investigations Programme Gardner *et al*. (2022) |  |  |  |  |
| PFOS | 0.0013–0.015 | 750 | Chemical Investigations Programme Gardner *et al*. (2022) | 8  (*Daphnia magna*; 21 days^1^) |  | 40  (*Daphnia magna*; 21 days^1^) | 5001.27  (*Daphnia magna*; 96 h^1^) |
| PFOS (surface water) | <0.00005–0.61 |  | Environment Agency (2021) | 8  (*Daphnia magna*; 21 days^1^) |  | 40  (*Daphnia magna*; 21 days^1^) | 5001.27  (*Daphnia magna*; 96 h^1^) |
|  | 0.088 | 2252 | Spurgeon *et al*. (2021) |  |  |  |  |
| PFOA | 0.0023–0.011 | 750 | Chemical Investigations Programme Gardner *et al*. (2022) | 160  (*Daphnia magna*; 21 days^1^) |  | 160  (*Daphnia magna*; 21 days^1^) | 75.3  (*Moina micrura*; 48 h^2^) |
| PFOA (surface water) | < 0.0002–0.073 |  | Environment Agency (2021) | 160  (*Daphnia magna* ; 21 days^1^) |  | 160  (*Daphnia magna*; 21 days^1^) | 75.3  (*Moina micrura*; 48 h^2^) |
|  | 0.16 | 2444 | Spurgeon *et al*. (2021) |  |  |  |  |
| PCB 52 | < 0.001 | 181 | Lu *et al*. (2015) |  |  |  |  |
| PCB 118 | < 0.001 | 179 | Lu *et al*. (2015) |  |  |  |  |
| PCB 152 | < 0.001 | 179 | Lu *et al*. (2015) |  |  |  |  |
| HCBDD | 0.00094–0.027 | 750 | Chemical Investigations Programme Gardner *et al*. (2022) |  |  |  |  |

^1^LOEC, lowest observed effect concentration; ^2^LC_10_, lethal concentration for 10%.

HCBDD, hexabromocyclododecane; PBDE, polybrominated diphenyl ethers; PCB, polychlorinated biphenyl; PFOA, perfluorooctanoic acid; PFOS, perfluorooctane sulfonate.

**Table S7.** Chemicals for which both measured environmental concentrations and lethal concentration at which 50% of the population is affected (LC_50_) 96 h toxicity information was available and thus were included in the Risk Quotient (RQ) analysis.

| **Pesticides** | **Metals** | **Petrochemicals** |
| --- | --- | --- |
| 2,4,5-Trichlorophenol | Cadmium | 1,1,2-Trichloroethane |
| 2,4,6-Trichlorophenol | Chromium | 1,2,3-Trichlorobenzene |
| 2,4-D | Copper | 1,2,4-Trichlorobenzene |
| Acetic acid | Lead | 4-Chloro-3-methylphenol |
| Alachlor | Zinc | Benzene |
| Aldicarb |  | Benzo(a)Anthracene |
| Atrazine |  | Benzo(e)Pyrene |
| Azinphos-methyl | | Fluoranthene |
| Azoxystrobin | | Methanol |
| Bifenthrin |  | Naphthalene |
| Carbaryl |  | Nitrobenzene |
| Carbendazim | | Phenanthrene |
| Chlorfenvinphos | | Tetrachloroethylene |
| Chlorpyrifos-ethyl | | Toluene |
| cis-Permethrin | |  |
| Cyfluthrin |  |  |
| Cypermethrin | |  |
| DDT-pp |  |  |
| Deltamethrin | |  |
| Diazinon |  |  |
| Dichlorvos |  |  |
| Dimethoate |  |  |
| Diuron |  |  |
| DNOC | | |
| Endosulfan A | |  |
| Fenitrothion |  |  |
| Fluazifop-butyl | |  |
| Fluazinam |  |  |
| Glyphosate |  |  |
| Lindane | | |
| Heptachlor |  |  |
| Iprodione |  |  |
| Lambda-cyhalothrin | |  |
| Malathion |  |  |
| Methiocarb |  |  |
| Methomyl |  |  |
| Methoxychlor | |  |
| Paclobutrazole | |  |
| Parathion-methyl | |  |
| Pentachlorophenol | |  |
| Permethrin | | |
| Pirimiphos-methyl | |  |
| Prochloraz |  |  |
| Prometryn |  |  |
| Propiconazole | |  |
| Propoxur |  |  |
| Simazine |  |  |
| Tecnazene |  |  |
| Terbutryn |  |  |
| trans-Permethrin | |  |

*DDT, dichlorodiphenyltrichloroethane; DNOC, dinitro-ortho-cresol; 2,4-D, 2,4-dichlorophenoxyacetic acid.


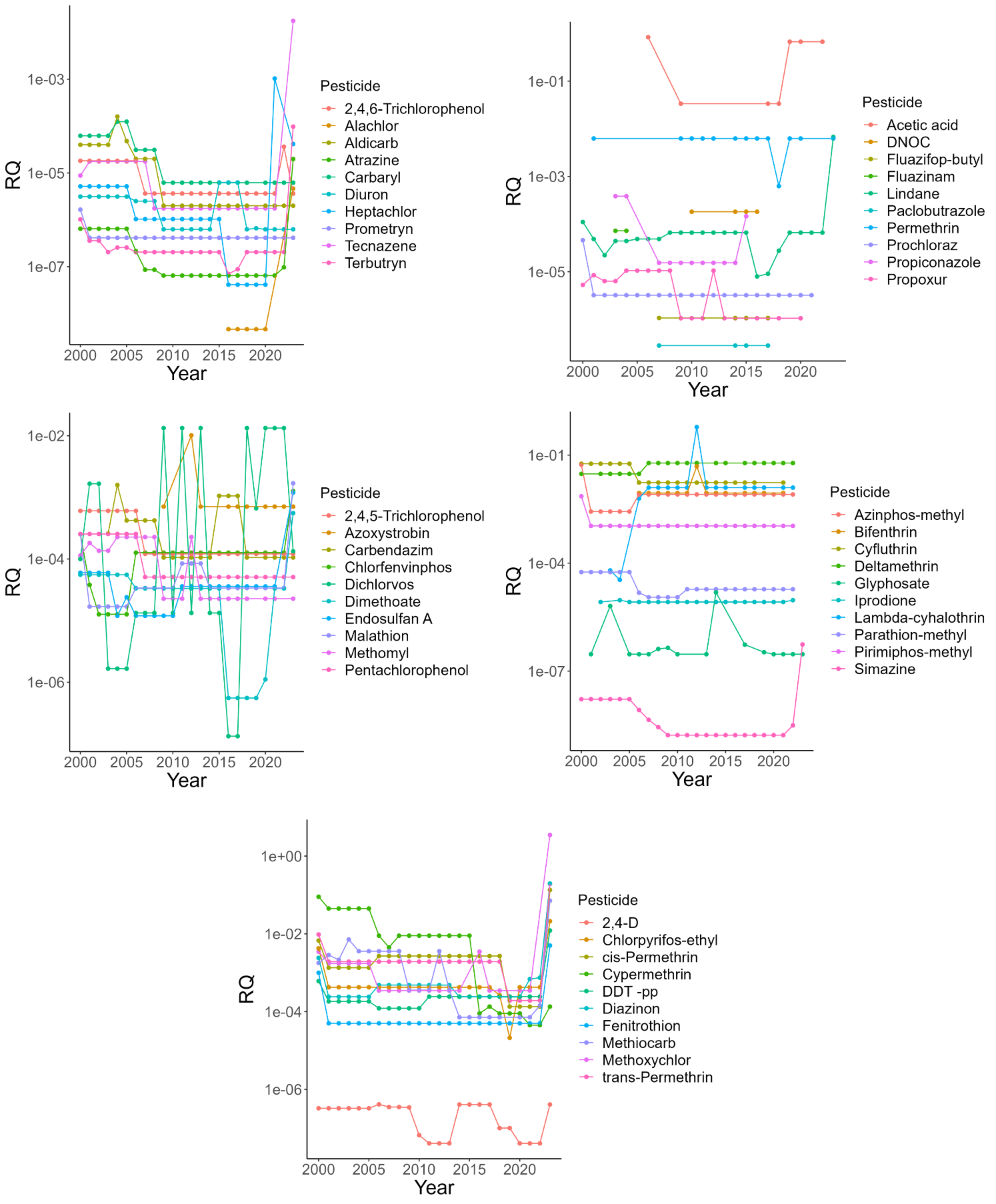


**Fig. S1.** Mean Risk Quotient (RQ) of pesticides over time from 2000 to 2023. See Table S8 for the number of field observations used to calculate the RQ per chemical per year. DDT, dichlorodiphenyltrichloroethane; DNOC, dinitro-ortho-cresol; 2,4-D, 2,4-dichlorophenoxyacetic acid.


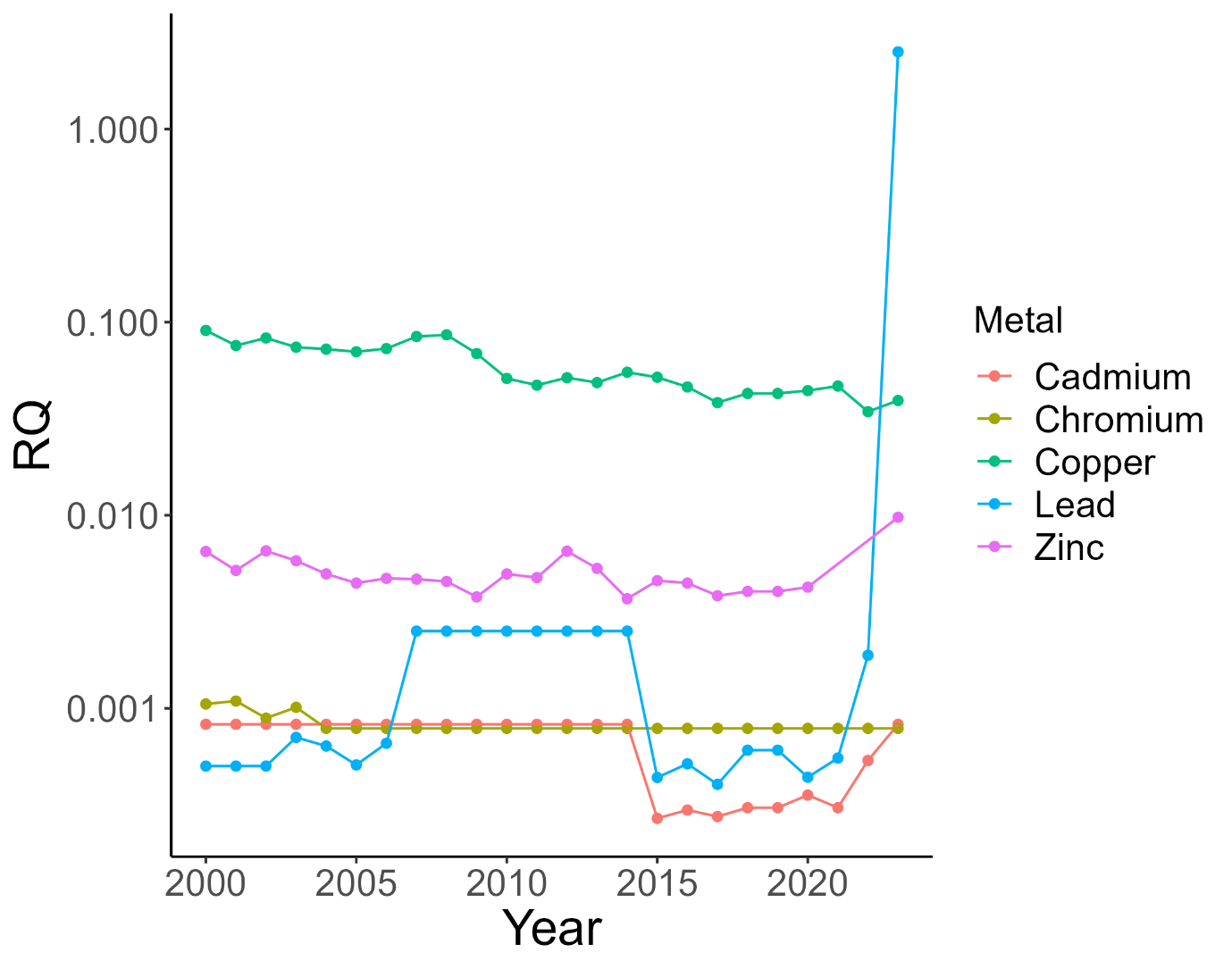


**Fig. S2.** Mean Risk Quotient (RQ) of metals over time from 2000 to 2023. See Table S8 for the number of field observations used to calculate the RQ per chemical per year.


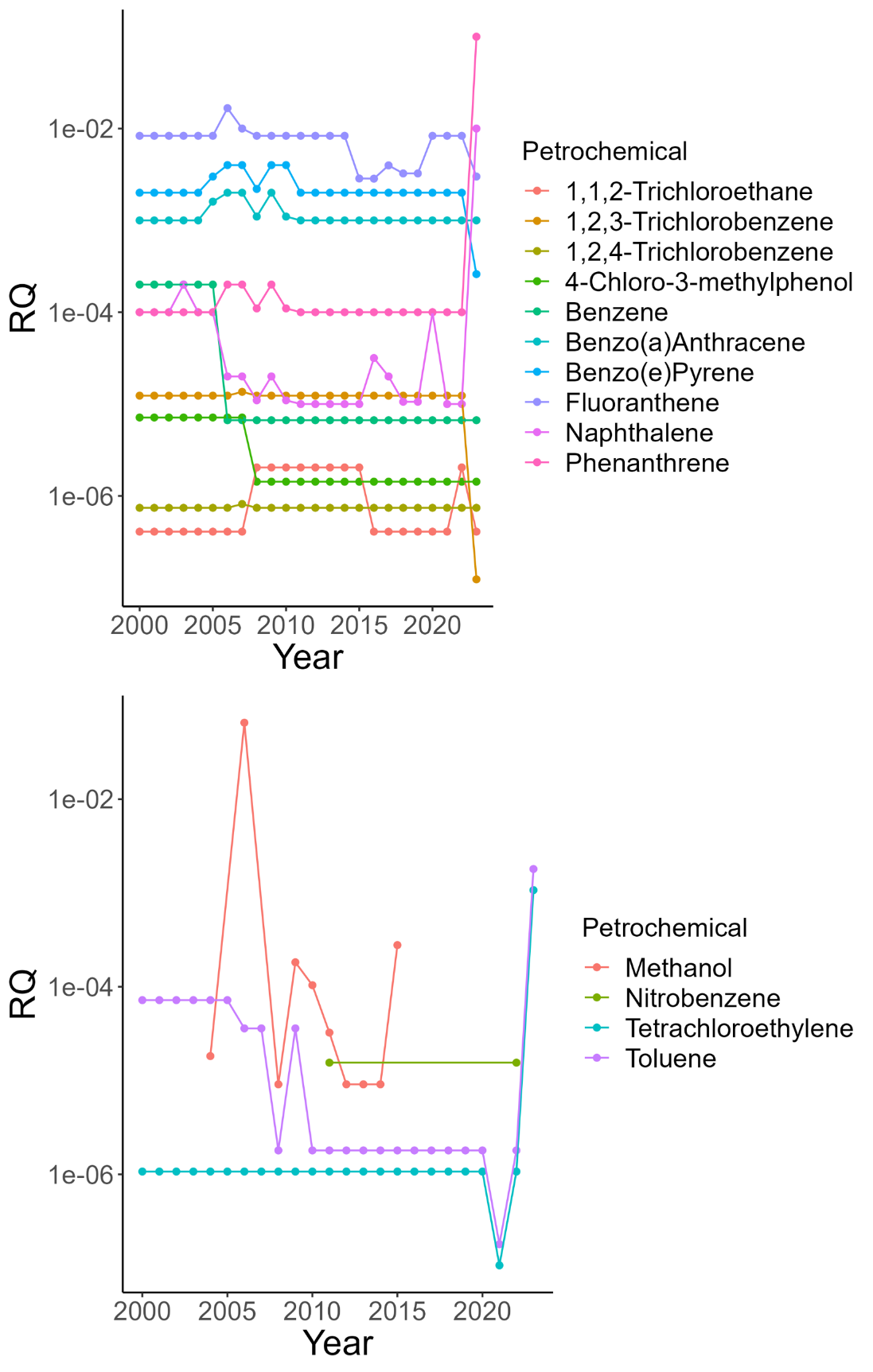


**Fig. S3.** Mean Risk Quotient (RQ) of petrochemicals over time from 2000 to 2023. See Table S8 for the number of field observations used to calculate the RQ per chemical per year.

**Table S8.** Number of observations of riverine concentrations for each chemical collected by the Environment Agency between 2000 and 2023. DDT, dichlorodiphenyltrichloroethane; DNOC, dinitro-ortho-cresol; 2,4-D, 2,4-dichlorophenoxyacetic acid.

| **Chemical group** | **Chemical** | **Number of observations per year** | | | | | | | | | | | | | | | | | | | | | | | | | **Total observations** | |
| --- | --- | --- | --- | --- | --- | --- | --- | --- | --- | --- | --- | --- | --- | --- | --- | --- | --- | --- | --- | --- | --- | --- | --- | --- | --- | --- | --- | --- |
|  |  | **2000** | **2001** | **2002** | **2003** | **2004** | **2005** | **2006** | **2007** | **2008** | **2009** | **2010** | **2011** | **2012** | **2013** | **2014** | **2015** | **2016** | **2017** | **2018** | **2019** | **2020** | **2021** | **2022** | **2023** |  | |  |
| Metal | Cadmium | 1852 | 2189 | 1980 | 1999 | 2008 | 2001 | 1707 | 1458 | 1061 | 844 | 1173 | 1637 | 2748 | 2981 | 5460 | 6336 | 5897 | 5343 | 4995 | 4403 | 1289 | 2797 | 2377 | 5812 | 70347 | |  |
| Metal | Chromium | 3857 | 4119 | 3824 | 4515 | 4292 | 4334 | 3418 | 2673 | 1980 | 1558 | 1867 | 1955 | 2312 | 2296 | 5245 | 6155 | 5405 | 4761 | 4697 | 3561 | 979 | 2143 | 1653 | 4471 | 82070 | |  |
| Metal | Copper | 17443 | 17666 | 17684 | 17794 | 17135 | 14875 | 14731 | 14341 | 14136 | 13902 | 11537 | 9870 | 9810 | 9534 | 6175 | 6823 | 6270 | 5432 | 5140 | 4479 | 1323 | 2871 | 2585 | 5543 | 247099 | |  |
| Metal | Lead | 3878 | 4314 | 4021 | 4674 | 4442 | 4484 | 3587 | 2798 | 1992 | 1544 | 1882 | 2315 | 2715 | 2757 | 5688 | 6553 | 5941 | 5292 | 5016 | 4410 | 1276 | 2788 | 2377 | 5830 | 90574 | |  |
| Metal | Zinc | 1827 | 2443 | 1795 | 1842 | 1917 | 1912 | 1633 | 1390 | 1109 | 885 | 1196 | 1642 | 1896 | 2401 | 5210 | 6088 | 5723 | 5175 | 4992 | 4272 | 1286 | 2802 | 2467 | 5391 | 67294 | |  |
| Pesticide | 2,4-D | 996 | 1004 | 1020 | 937 | 801 | 607 | 672 | 1610 | 870 | 806 | 800 | 654 | 675 | 739 | 862 | 707 | 328 | 430 | 122 | 69 | 31 | 618 | 764 | 644 | 16766 | |  |
| Pesticide | 2,4,5-Trichlorophenol | 127 | 120 | 119 | 121 | 102 | 110 | 90 | 78 | 33 | 183 | 95 | 41 | 46 | 47 | 116 | 98 | 90 | 73 | 106 | 18 | 13 | 180 | 25 | 378 | 2409 | |  |
| Pesticide | 2,4,6-Trichlorophenol | 340 | 298 | 302 | 280 | 262 | 243 | 194 | 180 | 47 | 188 | 147 | 44 | 50 | 47 | 185 | 162 | 154 | 136 | 158 | 55 | 22 | 180 | 25 | 378 | 4077 | |  |
| Pesticide | Acetic acid |  |  |  |  |  |  | 2 |  |  | 1 |  |  |  |  |  |  |  | 4 | 30 | 8 | 2 |  | 1 |  | 48 | |  |
| Pesticide | Alachlor |  |  |  |  |  |  |  |  |  |  |  |  |  |  |  |  | 696 | 1090 | 209 | 132 | 13 |  |  | 267 | 2407 | |  |
| Pesticide | Aldicarb | 150 | 176 | 178 | 199 | 195 | 173 | 194 | 183 | 61 | 28 | 19 | 15 | 23 | 19 | 18 | 17 | 17 | 7 | 1 |  | 8 | 522 | 689 | 804 | 3696 | |  |
| Pesticide | Atrazine | 896 | 871 | 953 | 834 | 830 | 802 | 871 | 678 | 723 | 1033 | 1149 | 972 | 878 | 876 | 1180 | 994 | 440 | 485 | 279 | 156 | 53 | 75 | 13 | 316 | 16357 | |  |
| Pesticide | Azinphos-methyl | 1042 | 787 | 892 | 858 | 839 | 740 | 773 | 602 | 578 | 814 | 921 | 802 | 688 | 689 | 971 | 784 | 299 | 346 | 122 | 28 | 17 | 49 | 3 |  | 13644 | |  |
| Pesticide | Azoxystrobin |  |  |  |  |  |  |  |  |  | 12 |  |  | 4 | 2 |  |  | 2 | 20 | 6 |  | 8 | 507 | 689 | 804 | 2054 | |  |
| Pesticide | Bifenthrin |  |  |  |  |  |  | 5 | 1 | 4 | 4 | 8 | 6 | 8 | 2 | 29 | 2 |  | 363 | 878 | 131 |  | 49 |  |  | 1490 | |  |
| Pesticide | Carbaryl | 217 | 231 | 260 | 278 | 273 | 236 | 272 | 265 | 141 | 96 | 79 | 25 | 21 | 19 | 18 | 17 | 17 | 8 | 3 |  | 8 | 522 | 689 | 804 | 4499 | |  |
| Pesticide | Carbendazim |  |  |  | 60 | 1 | 2 | 1 | 7 | 158 | 518 | 568 | 541 | 445 | 460 | 540 | 491 | 131 | 191 | 3 |  | 8 | 551 | 689 | 805 | 6170 | |  |
| Pesticide | Chlorfenvinphos | 701 | 591 | 638 | 550 | 516 | 507 | 513 | 456 | 427 | 677 | 783 | 646 | 567 | 597 | 820 | 670 | 307 | 373 | 143 | 44 | 22 | 49 | 3 | 290 | 10890 | |  |
| Pesticide | Chlorpyrifos-ethyl | 26 | 27 | 23 | 185 | 32 | 29 | 53 | 30 | 149 | 504 | 512 | 503 | 796 | 1061 | 745 | 501 | 139 | 185 | 12 | 7 | 12 | 44 | 3 | 273 | 5851 | |  |
| Pesticide | cis-Permethrin | 118 | 141 | 164 | 193 | 259 | 114 | 167 | 124 | 41 | 59 | 162 | 57 | 81 | 51 | 319 | 295 | 264 | 636 | 1024 | 554 | 139 | 937 | 716 | 755 | 7370 | |  |
| Pesticide | Cyfluthrin | 56 | 86 | 93 | 96 | 93 | 74 | 118 | 72 | 33 | 55 | 150 | 57 | 56 | 28 | 237 | 198 | 172 | 129 | 12 |  |  | 49 |  |  | 1864 | |  |
| Pesticide | Cypermethrin | 3 | 96 | 25 | 200 | 33 | 23 | 98 | 164 | 134 | 150 | 273 | 150 | 561 | 501 | 429 | 245 | 930 | 876 | 996 | 1123 | 441 | 1095 | 719 | 722 | 9987 | |  |
| Pesticide | DDT -pp | 615 | 596 | 543 | 668 | 642 | 602 | 658 | 558 | 562 | 636 | 694 | 881 | 919 | 854 | 949 | 835 | 755 | 746 | 842 | 619 | 141 | 8 | 3 | 302 | 14628 | |  |
| Pesticide | Deltamethrin | 6 |  | 3 | 104 | 3 | 4 | 5 | 1 | 4 | 4 | 8 | 6 | 6 |  | 23 | 2 |  | 325 | 783 | 462 | 87 | 447 | 101 |  | 2384 | |  |
| Pesticide | Diazinon | 877 | 803 | 824 | 718 | 694 | 686 | 693 | 676 | 606 | 832 | 926 | 673 | 619 | 669 | 846 | 676 | 987 | 1356 | 353 | 164 | 31 | 84 | 35 | 283 | 15111 | |  |
| Pesticide | Dichlorvos | 1042 | 974 | 952 | 876 | 851 | 744 | 782 | 690 | 643 | 847 | 977 | 845 | 724 | 726 | 1034 | 848 | 1041 | 1500 | 425 | 220 | 54 | 49 | 3 | 272 | 17119 | |  |
| Pesticide | Dimethoate | 301 | 385 | 374 | 369 | 276 | 67 | 83 | 60 | 162 | 502 | 536 | 516 | 575 | 659 | 677 | 508 | 824 | 1228 | 224 | 141 | 24 | 49 | 3 | 266 | 8809 | |  |
| Pesticide | Diuron | 570 | 622 | 597 | 543 | 516 | 489 | 535 | 514 | 479 | 717 | 756 | 630 | 595 | 557 | 653 | 595 | 241 | 291 | 61 | 20 | 15 | 539 | 689 | 812 | 12036 | |  |
| Pesticide | DNOC |  |  |  |  |  |  |  |  |  |  | 24 |  |  |  | 71 | 71 | 54 |  |  |  |  |  |  |  | 220 | |  |
| Pesticide | Endosulfan A | 654 | 576 | 574 | 628 | 597 | 514 | 550 | 388 | 278 | 274 | 317 | 290 | 269 | 245 | 367 | 278 | 109 | 91 | 62 | 28 | 9 | 1 |  | 274 | 7373 | |  |
| Pesticide | Fenitrothion | 1045 | 980 | 980 | 914 | 859 | 753 | 783 | 616 | 572 | 807 | 923 | 802 | 698 | 704 | 1002 | 825 | 340 | 394 | 155 | 45 | 22 | 49 | 3 | 270 | 14541 | |  |
| Pesticide | Fluazifop-butyl |  |  |  |  |  |  |  | 1 |  |  |  |  |  |  | 12 | 2 |  | 18 |  |  |  |  |  |  | 33 | |  |
| Pesticide | Fluazinam |  |  |  | 29 | 1 |  |  |  |  |  |  |  |  |  |  |  |  |  |  |  |  |  |  |  | 30 | |  |
| Pesticide | Glyphosate |  | 33 |  | 20 |  | 143 | 19 | 5 | 38 | 613 | 59 |  |  | 4 | 16 |  |  | 170 |  | 350 | 109 | 515 | 705 | 49 | 2848 | |  |
| Pesticide | Lindane | 859 | 801 | 712 | 810 | 764 | 710 | 780 | 688 | 622 | 709 | 789 | 737 | 697 | 646 | 716 | 626 | 988 | 1238 | 633 | 648 | 230 | 639 | 346 | 466 | 16854 | |  |
| Pesticide | Heptachlor | 344 | 328 | 360 | 364 | 326 | 315 | 367 | 227 | 65 | 46 | 41 | 44 | 47 | 48 | 58 | 38 | 751 | 1137 | 226 | 144 | 18 | 1 |  | 269 | 5564 | |  |
| Pesticide | Iprodione |  |  | 1 |  | 1 | 4 | 28 | 6 | 142 | 485 | 512 | 501 | 432 | 467 | 598 | 488 | 128 | 158 | 6 | 9 | 14 | 31 | 3 |  | 4014 | |  |
| Pesticide | Lambda-cyhalothrin |  |  |  | 183 | 8 |  | 5 | 1 | 4 | 4 | 6 | 6 | 55 | 2 | 29 | 2 |  | 381 | 895 | 131 |  | 49 | 14 |  | 1775 | |  |
| Pesticide | Malathion | 1044 | 954 | 985 | 920 | 853 | 751 | 781 | 614 | 576 | 823 | 951 | 830 | 706 | 716 | 1027 | 841 | 364 | 401 | 160 | 58 | 28 | 49 | 3 | 276 | 14711 | |  |
| Pesticide | Methiocarb | 25 | 23 | 12 | 146 | 32 | 21 | 36 | 27 | 4 | 83 | 5 | 1 | 5 | 3 | 2 | 2 | 2 | 2 | 3 |  | 8 | 507 | 689 | 804 | 2442 | |  |
| Pesticide | Methomyl | 25 | 24 | 12 | 21 | 24 | 21 | 36 | 28 | 4 | 15 | 5 | 1 | 5 | 3 | 2 | 2 | 2 | 2 | 3 |  | 8 | 522 | 689 | 799 | 2253 | |  |
| Pesticide | Methoxychlor | 1 | 25 | 25 | 20 | 20 | 27 | 51 | 8 | 9 | 3 | 1 | 4 | 8 | 10 | 17 |  | 13 | 7 | 4 |  | 2 | 1 |  | 1 | 257 | |  |
| Pesticide | Paclobutrazole |  |  |  |  |  |  |  | 1 |  |  |  |  |  |  | 12 | 2 |  | 18 |  |  |  |  |  |  | 33 | |  |
| Pesticide | Parathion-methyl | 656 | 571 | 574 | 504 | 468 | 468 | 488 | 419 | 517 | 781 | 896 | 766 | 667 | 684 | 971 | 784 | 298 | 347 | 125 | 28 | 17 | 49 | 3 |  | 11081 | |  |
| Pesticide | Pentachlorophenol | 691 | 702 | 680 | 637 | 563 | 549 | 518 | 424 | 336 | 599 | 565 | 474 | 479 | 478 | 577 | 528 | 337 | 343 | 336 | 239 | 71 | 180 | 23 | 488 | 10817 | |  |
| Pesticide | Permethrin |  | 21 |  |  |  |  |  |  |  | 15 | 94 | 16 | 15 |  | 230 | 238 | 219 | 216 | 151 | 67 | 17 |  |  | 5 | 1304 | |  |
| Pesticide | Pirimiphos-methyl | 23 | 20 | 23 | 25 | 25 | 28 | 52 | 30 | 149 | 498 | 512 | 499 | 432 | 471 | 599 | 495 | 132 | 195 | 6 | 1 | 11 | 44 | 3 |  | 4273 | |  |
| Pesticide | Prochloraz | 40 | 56 | 56 | 52 | 48 | 59 | 81 | 51 | 202 | 574 | 603 | 588 | 487 | 563 | 657 | 539 | 183 | 228 | 63 | 41 | 16 | 1 |  |  | 5188 | |  |
| Pesticide | Prometryn | 91 | 88 | 90 | 88 | 89 | 96 | 141 | 79 | 182 | 540 | 626 | 542 | 471 | 535 | 784 | 674 | 321 | 380 | 185 | 68 | 22 | 69 | 13 | 29 | 6203 | |  |
| Pesticide | Propiconazole |  |  |  | 192 | 11 |  |  | 10 | 21 | 47 | 47 | 46 | 47 | 47 | 36 | 2 |  |  |  |  |  |  |  |  | 506 | |  |
| Pesticide | Propoxur | 25 | 23 | 12 | 24 | 24 | 21 | 36 | 28 | 4 | 15 | 5 | 1 | 5 | 3 | 2 | 2 | 2 | 2 | 3 |  | 8 | 522 | 689 | 804 | 2260 | |  |
| Pesticide | Simazine | 891 | 870 | 915 | 789 | 789 | 778 | 943 | 1564 | 937 | 1061 | 1152 | 981 | 853 | 874 | 1180 | 995 | 442 | 502 | 286 | 165 | 53 | 75 | 13 | 312 | 17420 | |  |
| Pesticide | Tecnazene | 219 | 211 | 256 | 314 | 294 | 250 | 269 | 251 | 154 | 130 | 117 | 117 | 128 | 128 | 157 | 117 | 129 | 100 | 96 | 64 | 15 | 1 |  | 1 | 3518 | |  |
| Pesticide | Terbutryn | 126 | 113 | 114 | 109 | 85 | 95 | 141 | 88 | 204 | 588 | 676 | 586 | 514 | 581 | 797 | 674 | 992 | 1415 | 395 | 200 | 35 | 67 | 13 | 285 | 8893 | |  |
| Pesticide | trans-Permethrin | 118 | 141 | 164 | 193 | 259 | 114 | 168 | 124 | 41 | 59 | 162 | 57 | 55 | 29 | 296 | 257 | 235 | 590 | 962 | 504 | 116 | 948 | 714 | 703 | 7009 | |  |
| Petrochemical | 1,1,2-Trichloroethane | 406 | 442 | 454 | 379 | 345 | 252 | 292 | 162 | 153 | 314 | 201 | 205 | 192 | 153 | 243 | 165 | 13 | 71 | 68 | 56 | 39 | 29 | 31 | 373 | 5038 | |  |
| Petrochemical | 1,2,3-Trichlorobenzene | 572 | 530 | 473 | 525 | 482 | 470 | 531 | 377 | 368 | 456 | 534 | 474 | 455 | 411 | 488 | 386 | 204 | 238 | 201 | 458 | 223 | 29 | 10 | 314 | 9209 | |  |
| Petrochemical | 1,2,4-Trichlorobenzene | 572 | 530 | 471 | 525 | 482 | 470 | 518 | 381 | 368 | 456 | 534 | 475 | 455 | 408 | 488 | 386 | 205 | 242 | 199 | 476 | 230 | 29 | 11 | 167 | 9078 | |  |
| Petrochemical | 4-Chloro-3-methylphenol | 654 | 642 | 629 | 587 | 458 | 251 | 264 | 203 | 30 | 162 | 68 | 28 | 32 | 40 | 76 | 44 | 19 | 26 | 76 | 8 | 9 | 180 | 25 | 379 | 4890 | |  |
| Petrochemical | Benzene | 606 | 594 | 603 | 607 | 475 | 273 | 288 | 205 | 236 | 336 | 214 | 354 | 427 | 382 | 554 | 582 | 656 | 743 | 886 | 1035 | 452 | 96 | 91 | 460 | 11155 | |  |
| Petrochemical | Benzo(a)Anthracene | 323 | 263 | 319 | 293 | 295 | 288 | 275 | 184 | 60 | 101 | 97 | 115 | 160 | 154 | 99 | 85 | 46 | 115 | 176 | 147 | 41 | 27 | 24 | 313 | 4000 | |  |
| Petrochemical | Benzo(a)Pyrene | 463 | 350 | 381 | 430 | 501 | 513 | 510 | 497 | 529 | 631 | 661 | 641 | 1114 | 1296 | 511 | 922 | 3726 | 3580 | 1364 | 1310 | 253 | 733 | 925 | 903 | 22744 | |  |
| Petrochemical | Fluoranthene | 465 | 351 | 389 | 388 | 501 | 510 | 503 | 488 | 512 | 625 | 661 | 641 | 636 | 612 | 253 | 772 | 3611 | 3108 | 1246 | 930 | 145 | 747 | 920 | 886 | 19900 | |  |
| Petrochemical | Methanol |  |  |  |  | 3 |  | 2 |  | 2 | 4 | 4 | 4 | 1 | 3 | 1 |  | 2 |  |  |  |  |  |  | 14 | 40 | |  |
| Petrochemical | Naphthalene | 467 | 476 | 474 | 358 | 409 | 204 | 208 | 135 | 43 | 121 | 132 | 144 | 181 | 159 | 140 | 110 | 45 | 124 | 190 | 152 | 69 | 35 | 27 | 321 | 4724 | |  |
| Petrochemical | Nitrobenzene |  |  |  |  |  |  |  |  |  |  |  | 1 |  |  |  |  |  |  |  |  |  |  | 1 |  | 2 | |  |
| Petrochemical | Phenanthrene | 321 | 260 | 318 | 296 | 294 | 294 | 275 | 182 | 54 | 97 | 93 | 111 | 156 | 155 | 96 | 81 | 42 | 110 | 177 | 120 | 38 | 27 | 24 | 323 | 3944 | |  |
| Petrochemical | Tetrachloroethylene | 736 | 684 | 753 | 797 | 716 | 708 | 703 | 496 | 515 | 742 | 648 | 531 | 463 | 429 | 526 | 453 | 297 | 341 | 340 | 269 | 107 | 93 | 69 | 477 | 11893 | |  |
| Petrochemical | Toluene | 676 | 660 | 683 | 679 | 531 | 335 | 344 | 242 | 189 | 371 | 269 | 157 | 101 | 98 | 201 | 164 | 131 | 210 | 225 | 188 | 76 | 60 | 42 | 404 | 7036 | |  |

**References**

Agra, A.R., Soares, A.M.V.M. & Barata, C. (2011). Life-history consequences of adaptation to pollution. ‘*Daphnia longispina* clones historically exposed to copper’. *Ecotoxicology* **20**, 552–562.

Ashton, D., Hilton, M. & Thomas, K. V. (2004). Investigating the environmental transport of human pharmaceuticals to streams in the United Kingdom. *Science of The Total Environment* **333**, 167–184.

Balashova, N., Hiscock, K.M., Reid, B.J. & Reynolds, R. (2021). Trends in metaldehyde concentrations and fluxes in a lowland, semi-agricultural catchment in the UK (2008–2018). *Science of The Total Environment* **795**, 148858.

Baudouin, M.F. & Scoppa, P. (1974). Acute toxicity of various metals to freshwater zooplankton. *The Bulletin of Environmental Contamination and Toxicology* **12**, 745–751.

Bearcock, J.M., Smedley, P.L., Fordyce, F.M., Everett, P.A. & Ander, E.L. (2017). Controls on surface water quality in the River Clyde catchment, Scotland, UK, with particular reference to chromium and lead. *Earth and Environmental Science Transactions of The Royal Society of Edinburgh* **108**, 249–267.

Besser, J.M., Ivey, C.D., Steevens, J.A., Cleveland, D., Soucek, D., Dickinson, A., Van Genderen, E.J., Ryan, A.C., Schlekat, C.E., Garman, E., Middleton, E. & Santore, R. (2021). Modeling the Bioavailability of Nickel and Zinc to Ceriodaphnia dubia and Neocloeon triangulifer in Toxicity Tests with Natural Waters. *Environmental Toxicology and Chemistry* **40**, 3049–3062.

Bianchini, A. & Wood, C.M. (2008). Does sulfide or water hardness protect against chronic silver toxicity in Daphnia magna? A critical assessment of the acute-to-chronic toxicity ratio for silver. *Ecotoxicology and Environmental Safety* **71**, 32–40.

Bielmyer, G.K., Bell, R.A. & Klaine, S.J. (2002). Effects of ligand-bound silver on Ceriodaphnia dubia. *Environmental Toxicology and Chemistry* **21**, 2204–2208.

Biesinger, K.E. & Christensen, G.M. (2011). Effects of Various Metals on Survival, Growth, Reproduction, and Metabolism of *Daphnia magna*. *Journal of the Fisheries Board of Canada* **29**, 1691–1700.

Birge, W.J., Black, J.A., Westerman, A.G., Short, T.M., Taylor, S.B., Bruser, D.M. & Wallingford, E. (1985). Recommendations on Numerical Values for Regulating Iron and Chloride Concentrations for the Purpose of Protecting Warmwater Species of Aquatic Life in the Commonwealth of Kentucky. *Kentucky Energy and Environment Cabinet*, 1 – 74.

Boxall, A.B.A., Fogg, L., Blackwell, P.A., Kay, P. & Pemberton, E.J. (2002). Review of veterinary medicines in the environment. Environment Agency, 1 – 251.

Braginskij, L.P. & Shcherban, E.P. (1979). Acute toxicity of heavy metals to aquatic invertebrates under different temperature conditions. *Hydrobiology* **14**, 78–82.

Braginskiy, L.P. & Shcherban, E.P. (1978). Water toxicology and radioecology. Acute toxicity of heavy metals to aquatic invertebrates at different temperatures. *Hydrobiology* **14**, 78–82.

Breitholtz, M. & Bengtsson, B.E. (2001). Oestrogens have no Hormonal Effect on the Development and Reproduction of the Harpacticoid Copepod Nitocra spinipes. *Marine Pollution Bulletin* **42**, 879–886.

Bubb, J.M. & Lester, J.N. (1994). Anthropogenic heavy metal inputs to lowland river systems, a case study. The River Stour, U.K. *Water, Air, & Soil Pollution* **78**, 279–296.

Buglife (2017). Neonictinoid Insecticides in British Freshwaters 2016 Water Framework Directive Watch List Monitoring Results and Recommendations. *Buglife*, 1-66.

Buglife (2021). Pharmaceuticals in freshwater environments and their potential effects on freshwater invertebrates. *Buglife*, 1-63.

Burns, E.E., Carter, L.J., Kolpin, D.W., Thomas-Oates, J. & Boxall, A.B.A. (2018). Temporal and spatial variation in pharmaceutical concentrations in an urban river system. *Water Research* **137**, 72–85.

Burns, E.E., Thomas-Oates, J., Kolpin, D.W., Furlong, E.T. & Boxall, A.B.A. (2017). Are exposure predictions, used for the prioritization of pharmaceuticals in the environment, fit for purpose? *Environmental Toxicology and Chemistry* **36**, 2823–2832.

Buss, J.M. & Lester, J.N. (1995). The effect of final sewage effluent discharges upon the behaviour and fate of metals in a lowland river system.A question of dillition? *Environmental Technology (United Kingdom)* **16**, 401–417.

Casado, J., Santillo, D. & Johnston, P. (2018). Multi-residue analysis of pesticides in surface water by liquid chromatography quadrupole-Orbitrap high resolution tandem mass spectrometry. *Analytica Chimica Acta* **1024**, 1–17.

Casper, S.T., Mehra, A., Farago, M.E. & Gill, R.A. (2004). Contamination of surface soils, river water and sediments by trace metals from copper processing industry in the Churnet River Valley, Staffordshire, UK. *Environmental Geochemistry and Health* **26**, 59–67.

Castle, G.D., Mills, G.A., Bakir, A., Gravell, A., Schumacher, M., Snow, K. & Fones, G.R. (2018). Measuring metaldehyde in surface waters in the UK using two monitoring approaches. *Environmental Science: Processes & Impacts* **20**, 1180–1190.

Castle, G.D., Mills, G.A., Gravell, A., Leggatt, A., Stubbs, J., Davis, R. & Fones, G.R. (2019). Comparison of different monitoring methods for the measurement of metaldehyde in surface waters. *Environmental Monitoring and Assessment* **191**, 1–13.

Chapman, G.A., Ota, S. & Recht, F. (1980). Effects of Water Hardness on the Toxicity of Metals to *Daphnia magna*. *Desalination and Water Treatment* **184**, 80-85.

Cherry, D.S., J.H., Jr. Rodgers, Graney, R.L. & Cairns, J.C. (1980). Dynamics and control of the Asiatic Clam in the New River, Virginia. *Virginia Water Resources Research Center* **123**, 1-83.

Comber, S., Mistry, R. & Sturdy, L. (2012). Scoping study for Dangerous Substances Directive List II chemicals by Water Framework Directive-United Kingdom Technical Advisory Group (WFD-UKTAG). *Water Framework Directive,* 1-44.

Cooper, N.L., Bidwell, J.R. & Kumar, A. (2009). Toxicity of copper, lead, and zinc mixtures to *Ceriodaphnia dubia* and *Daphnia carinata*. *Ecotoxicology and Environmental Safety* **72**, 1523–1528.

Crémazy, A., Brix, K. V. & Wood, C.M. (2018). Chronic Toxicity of Binary Mixtures of Six Metals (Ag, Cd, Cu, Ni, Pb, and Zn) to the Great Pond Snail *Lymnaea stagnalis.* *Environmental Science and Technology* **52**, 5979–5988.

Croll, B.T. (1991). Pesticides in Surface Waters and Groundwaters. *Water and Environment Journal* **5**, 389–395.

De Schamphelaere, K., Laer, L.V., Deleebeeck, N., Muyssen, B.T., Degryse, F., Smolders, E. & Janssen, C. (2006). Nickel Speciation and Ecotoxicity in European Natural Surface Waters: Development, Refinement and Validation of Bioavailability Models. Ghent University. Laboratory of Environmental Toxicology and Chemistry, 1-125.

De Schamphelaere, K.A.C., Koene, J.M., Heijerick, D.G. & Janssen, C.R. (2008). Reduction of growth and haemolymph Ca levels in the freshwater snail Lymnaea stagnalis chronically exposed to cobalt. *Ecotoxicology and Environmental Safety* **71**, 65–70.

Deepartnership (2022) Water temperature research. Deepartnership, 1.

Dixon, E. & Gardner, M. (2014). Chemical Speciation & Bioavailability Reactive aluminium in UK surface waters. *Chemical Speciation & Bioavailability* **10**, 11–17.

Dowson, P., Chem, C., Biol, C., Scrimshaw, M.D., Nasir, J.M., Bubb, J.N. & Lester, J.N. (1996). The environmental impact of a chemical spill from a timber-treatment works on a lowland river system. *Water and Environment Journal* **10**, 235–244.

Du, J., Mei, C.F., Ying, G.G. & Xu, M.Y. (2016). Toxicity thresholds for Diclofenac, Acetaminophen and Ibuprofen in the Water Flea *Daphnia magna*. *Bulletin of Environmental Contamination and Toxicology* **97**, 84–90.

Egli, M., Rapp-Wright, H., Oloyede, O., Francis, W., Preston-Allen, R., Friedman, S., Woodward, G., Piel, F.B. & Barron, L.P. (2023). A One-Health environmental risk assessment of contaminants of emerging concern in London’s waterways throughout the SARS-CoV-2 pandemic. *Environment International* **180**, 108210.

Elnabarawy, M.T., Welter, A.N. & Robideau, R.R. (1986). Relative sensitivity of three daphnid species to selected organic and inorganic chemicals. *Environmental Toxicology and Chemistry* **5**, 393–398.

Enserink, E.L., Maas-Diepeveen, J.L. & Van Leeuwen, C.J. (1991). Combined effects of metals; an ecotoxicological evaluation. *Water Research* **25**, 679–687.

Enserink, L., de la Haye, M. & Maas, H. (1993). Reproductive strategy of *Daphnia magna*: implications for chronic toxicity tests. *Aquatic Toxicology* **25**, 111–123.

Environment Agency (2005). The effect of the Voluntary Initiative on water quality. *Environment Agency*, 1-151.

Environment Agency (2006) Targeted monitoring study for veterinary medicines in the environment. *Environment Agency*, 1-120.

Environment Agency (2007*a*) Climate change impacts and water temperature. *Environment Agency*, 1-111.

Environment Agency (2008*c*) Science Report-Assessment of Metal Mining-Contaminated River Sediments in England and Wales i Assessment of Metal Mining-Contaminated River Sediments in England and Wales. *Environment Agency*, 1-64.

Environment Agency (2019*a*) Cypermethrin: Sources, pathways and environmental data. *Environment Agency*, 1-39.

Environment Agency (2021) Poly- and perfluoroalkyl substances (PFAS): sources, pathways and environmental data - report. *Environment Agency*, 1-110.

Esbaugh, A.J., Brix, K. V., Mager, E.M. & Grosell, M. (2011). Multi-linear regression models predict the effects of water chemistry on acute lead toxicity to *Ceriodaphnia dubia* and *Pimephales promelas*. *Comparative Biochemistry and Physiology Part C: Toxicology & Pharmacology* **154**, 137–145.

Esbaugh, A.J., Brix, K. V., Mager, E.M., De Schamphelaere, K. & Grosell, M. (2012). Multi-linear regression analysis, preliminary biotic ligand modeling, and cross species comparison of the effects of water chemistry on chronic lead toxicity in invertebrates. *Comparative Biochemistry and Physiology Part C: Toxicology & Pharmacology* **155**, 423–431.

Fargašová, A. (1997). Comparative Study of Ecotoxicological Effect of Triorganotin Compounds on Various Biological Subjects. *Ecotoxicology and Environmental Safety* **36**, 38–42.

Faria, M., López, M.A., Fernández-Sanjuan, M., Lacorte, S. & Barata, C. (2010). Comparative toxicity of single and combined mixtures of selected pollutants among larval stages of the native freshwater mussels (*Unio elongatulus*) and the invasive zebra mussel (*Dreissena polymorpha*). *Science of The Total Environment* **408**, 2452–2458.

Farris, J.L., Belanger, S.E., Cherry, D.S. & Cairns, J. (1989). Cellulolytic activity as a novel approach to assess long-term zinc stress to Corbicula. *Water Research* **23**, 1275–1283.

Gale, N.L., Wixson, B.G. & Erten, M. (1992). An Evaluation of the Acute Toxicity of Lead, Zinc, and Cadmium in Missouri Ozark Groundwater. *Trace Substances in Environmental Health* **25**, 169–183.

Gardner, M.J., Comber, S.D.W. & Ellor, B. (2022). Summary of data from the UKWIR chemical investigations programme and a comparison of data from the past ten years’ monitoring of effluent quality. *Science of The Total Environment* **832**, 155041.

Geffard, O., Xuereb, B., Chaumot, A., Geffard, A., Biagianti, S., Noël, C., Abbaci, K., Garric, J., Charmantier, G. & Charmantier-Daures, M. (2010). Ovarian cycle and embryonic development in Gammarus fossarum: Application for reproductive toxicity assessment. *Environmental Toxicology and Chemistry* **29**, 2249–2259.

Gower, A.M., Myers, G., Kent, M. & Foulkes, M.E. (1994). Relationships between macroinvertebrate communities and environmental variables in metal-contaminated streams in south-west England. *Freshwater Biology* **32**, 199–221.

Griffitt, R.J., Luo, J., Gao, J., Bonzongo, J.C. & Barber, D.S. (2008). Effects of particle composition and species on toxicity of metallic nanomaterials in aquatic organisms. *Environmental Toxicology and Chemistry* **27**, 1972–1978.

Haeba, M.H., Hilscherová, K., Mazurová, E. & Bláha, L. (2008). Selected endocrine disrupting compounds (Vinclozolin, Flutamide, Ketoconazole and Dicofol): Effects on survival, occurrence of males, growth, molting and reproduction of *Daphnia magna*. *Environmental Science and Pollution Research* **15**, 222–227.

Hansen, L.K., Frost, P.C., Larson, J.H. & Metcalfe, C.D. (2008). Poor elemental food quality reduces the toxicity of fluoxetine on *Daphnia magna*. *Aquatic Toxicology* **86**, 99–103.

Hilton, M.J. & Thomas, K. V. (2003). Determination of selected human pharmaceutical compounds in effluent and surface water samples by high-performance liquid chromatography–electrospray tandem mass spectrometry. *Journal of Chromatography A* **1015**, 129–141.

Hu, J., Wang, D., Forthaus, B.E. & Wang, J. (2012). Quantifying the effect of nanoparticles on As(V) ecotoxicity exemplified by nano-Fe2O3 (magnetic) and nano-Al2O3. *Environmental Toxicology and Chemistry* **31**, 2870–2876.

Huggett, D.B., Brooks, B.W., Peterson, B., Foran, C.M. & Schlenk, D. (2002). Toxicity of select beta adrenergic receptor-blocking pharmaceuticals (B-blockers) on aquatic organisms. *Archives of Environmental Contamination and Toxicology* **43**, 229–235.

Jarvie, H.P., Neal, C., Burton, J.D. & Tappin, A.D. (2000). Patterns in trace element chemistry in the freshwater tidal reaches of the River Trent. *Science of The Total Environment* **251**–**252**, 317–333.

Jarvie, H.P., Neal, C., Rowland, A.P., Neal, M., Morris, P.N., Lead, J.R., Lawlor, A.J., Woods, C., Vincent, C., Guyatt, H. & Hockenhull, K. (2012). Role of riverine colloids in macronutrient and metal partitioning and transport, along an upland–lowland land-use continuum, under low-flow conditions. *Science of The Total Environment* **434**, 171–185.

Johnson, A.C., Donnachie, R.L., Sumpter, J.P., Jürgens, M.D., Moeckel, C. & Pereira, M.G. (2017). An alternative approach to risk rank chemicals on the threat they pose to the aquatic environment. *Science of The Total Environment* **599**–**600**, 1372–1381.

Kalender, L. (2010). Drainage Geochemistry Around the Abandoned Whittle Colliery, Northumberland, Uk. *Arabian Journal for Science and Engineering* **35**, 145–164.

Kasprzyk-Hordern, B., Dinsdale, R.M. & Guwy, A.J. (2008). The occurrence of pharmaceuticals, personal care products, endocrine disruptors and illicit drugs in surface water in South Wales, UK. *Water Research* **42**, 3498–3518.

Kay, P. & Grayson, R. (2014). Using water industry data to assess the metaldehyde pollution problem. *Water and Environment Journal* **28**, 410–417.

Kay, P., Hughes, S.R., Ault, J.R., Ashcroft, A.E. & Brown, L.E. (2017). Widespread, routine occurrence of pharmaceuticals in sewage effluent, combined sewer overflows and receiving waters. *Environmental Pollution* **220**, 1447–1455.

Keithly, J., Brooker, J.A., Deforest, D.K., Wu, B.K. & Brix, K. V. (2004). Acute and chronic toxicity of nickel to a cladoceran (Ceriodaphnia dubia) and an amphipod (*Hyalella azteca*). *Environmental Toxicology and Chemistry* **23**, 691–696.

Kolkmeier, M.A. & Brooks, B.W. (2013). Sublethal silver and NaCl toxicity in *Daphnia magna*: A comparative study of standardized chronic endpoints and progeny phototaxis. *Ecotoxicology* **22**, 693–706.

Lathouri, M. & Korre, A. (2015). Temporal assessment of copper speciation, bioavailability and toxicity in UK freshwaters using chemical equilibrium and biotic ligand models: Implications for compliance with copper environmental quality standards. *Science of The Total Environment* **538**, 385–401.

Lawlor, A.J. & Tipping, E. (2003). Metals in bulk deposition and surface waters at two upland locations in northern England. *Environmental Pollution* **121**, 153–167.

Lee, S., Jung, D., Kho, Y., Ji, K., Kim, P., Ahn, B. & Choi, K. (2015). Ecotoxicological assessment of cimetidine and determination of its potential for endocrine disruption using three test organisms: *Daphnia magna*, *Moina macrocopa*, and *Danio rerio*. *Chemosphere* **135**, 208–216.

Lee, S., Kim, C., Liu, X., Lee, S., Kho, Y., Kim, W.K., Kim, P. & Choi, K. (2021). Ecological risk assessment of amoxicillin, enrofloxacin, and neomycin: Are their current levels in the freshwater environment safe? *Toxics* **9**, 196.

Levy, L., Rushton, L. & Shuker, L. (1999). Institute for Environment and Health IEH report on benzene in the environment. *Occupational and Environmental Medicine* **58**, 2-13.

Li, S.W., Wang, Y.H. & Lin, A.Y.C. (2017). Ecotoxicological effect of ketamine: Evidence of acute, chronic and photolysis toxicity to *Daphnia magna*. *Ecotoxicology and Environmental Safety* **143**, 173–179.

Liber, K., De Rosemond, S. & Budnick, K. (2007). Uranium Toxicity to Regionally-Representative Algae and Invertebrate Species. *Canadian Council of Ministers of the Environment*, 1-121.

Long, J.L.A., House, W.A., Parker, A. & Rae, J.E. (1998). Micro-organic compounds associated with sediments in the Humber rivers. *Science of The Total Environment* **210**–**211**, 229–253.

Lu, Q., Johnson, A.C., Jürgens, M.D., Sweetman, A., Jin, L. & Whitehead, P. (2015). The distribution of Polychlorinated Biphenyls (PCBs) in the River Thames Catchment under the scenarios of climate change. *Science of The Total Environment* **533**, 187–195.

Lundy, L., Alves, L., Revitt, M. & Wildeboer, D. (2017). Metal Water-Sediment Interactions and Impacts on an Urban Ecosystem. *International Journal of Environmental Research and Public Health 2017, Vol. 14, Page 722* **14**, 722.

Madoni, P. (2000). The acute toxicity of nickel to freshwater ciliates. *Environmental Pollution* **109**, 53–59.

Mager, E.M., Brix, K. V., Gerdes, R.M., Ryan, A.C. & Grosell, M. (2011) Effects of water chemistry on the chronic toxicity of lead to the cladoceran, *Ceriodaphnia dubia*. *Ecotoxicology and Environmental Safety* **74**, 238–243.

Mayes, W.M., Potter, H.A.B. & Jarvis, A.P. (2010). Inventory of aquatic contaminant flux arising from historical metal mining in England and Wales. *Science of The Total Environment* **408**, 3576–3583.

Meyer, J.S., Boese, C.J. & Collyard, S.A. (2002). Whole-body accumulation of copper predicts acute toxicity to an aquatic oligochaete (*Lumbriculus variegatus*) as pH and calcium are varied. *Comparative Biochemistry and Physiology Part C: Toxicology & Pharmacology* **133**, 99–109.

Minagh, E., Hernan, R., O’Rourke, K., Lyng, F.M. & Davoren, M. (2009). Aquatic ecotoxicity of the selective serotonin reuptake inhibitor sertraline hydrochloride in a battery of freshwater test species. *Ecotoxicology and Environmental Safety* **72**, 434–440.

Minguez, L., Farcy, E., Ballandonne, C., Lepailleur, A., Serpentini, A., Lebel, J.M., Bureau, R. & Halm-Lemeille, M.P. (2014). Acute toxicity of 8 antidepressants: What are their modes of action? *Chemosphere* **108**, 314–319.

Munzinger, A. & Monicelli, F. (1991). A Comparison of the Sensitivity of Three *Daphnia magna* Populations Under Chronic Heavy Metal Stress. *Ecotoxicology and Environmental Saftey* **22**, 24–31.

Muyssen, B.T.A. & Janssen, C.R. (2007). Age and exposure duration as a factor influencing Cu and Zn toxicity toward *Daphnia magna*. *Ecotoxicology and Environmental Safety* **68**, 436–442.

Nagar, Y., Thakur, R.S., Parveen, T., Patel, D.K., Ram, K.R. & Satish, A. (2020). Toxicity assessment of parabens in *Caenorhabditis elegans*. *Chemosphere* **246**, 125730.

Nair, P.M.G., Park, S.Y., Lee, S.W. & Choi, J. (2011). Differential expression of ribosomal protein gene, gonadotrophin releasing hormone gene and Balbiani ring protein gene in silver nanoparticles exposed *Chironomus riparius*. *Aquatic Toxicology* **101**, 31–37.

Neal, C., Jarvie, H.P., Whitton, B.A. & Gemmell, J. (2000*a*). The water quality of the River Wear, north-east England. *Science of The Total Environment* **251**–**252**, 153–172.

Neal, C., Neal, M., Hill, L. & Wickham, H. (2006). The water quality of the River Thame in the Thames Basin of south/south-eastern England. *Science of The Total Environment* **360**, 254–271.

Neal, C. & Robson, A.J. (2000). A summary of river water quality data collected within the Land–Ocean Interaction Study: core data for eastern UK rivers draining to the North Sea. *Science of The Total Environment* **251**–**252**, 585–665.

Neal, C., Smith, C.J., Jeffery, H.A., Jarvie, H.P. & Robson, A.J. (1996) Trace element concentrations in the major rivers entering the Humber estuary, NE England. *Journal of Hydrology* **182**, 37–64. Elsevier.

Neal, C., Williams, R.J., Neal, M., Bhardwaj, L.C., Wickham, H., Harrow, M. & Hill, L.K. (2000*b*) The water quality of the River Thames at a rural site downstream of Oxford. *Science of The Total Environment* **251**–**252**, 441–457. Elsevier.

Niemi, L., Landová, P., Taggart, M., Boyd, K., Zhang, Z. & Gibb, S. (2022). Spatiotemporal trends and annual fluxes of pharmaceuticals in a Scottish priority catchment. *Environmental Pollution* **292**, 118295.

Nunes, B., Antunes, S.C., Santos, J., Martins, L. & Castro, B.B. (2014). Toxic potential of paracetamol to freshwater organisms: A headache to environmental regulators? *Ecotoxicology and Environmental Safety* **107**, 178–185.

Nys, C., Janssen, C.R. & De Schamphelaere, K.A.C. (2017). The effect of pH on chronic zinc toxicity differs between daphnid species: Development of a preliminary chronic zinc *Ceriodaphnia dubia* bioavailability model. *Environmental Toxicology and Chemistry* **36**, 2750–2755.

Oda, S., Tatarazako, N., Watanabe, H., Morita, M. & Iguchi, T. (2006). Genetic differences in the production of male neonates in *Daphnia magna* exposed to juvenile hormone analogs. *Chemosphere* **63**, 1477–1484.

Oropesa, A.L., Floro, A.M. & Palma, P. (2017). Toxic potential of the emerging contaminant nicotine to the aquatic ecosystem. *Environmental Science and Pollution Research* **24**, 16605–16616.

Palumbo-Roe, B., Banks, V.J., Bonsor, H.C., Hamilton, E.M. & Watts, M.J. (2017). Limitations on the role of the hyporheic zone in chromium natural attenuation in a contaminated urban stream. *Applied Geochemistry* **83**, 108–120.

Palumbo-Roe, B., Wragg, J. & Banks, V.J. (2012). Lead mobilisation in the hyporheic zone and river bank sediments of a contaminated stream: Contribution to diffuse pollution. *Journal of Soils and Sediments* **12**, 1633–1640.

Perkins, R., Whitehead, M., Civil, W. & Goulson, D. (2021). Potential role of veterinary flea products in widespread pesticide contamination of English rivers. *Science of The Total Environment* **755**, 143560.

Pokethitiyook, P., Upatham, E.S. & Leelhaphunt, O. (1987). Acute Toxicity of Various Metals to *Moina macrocopa*. *Natural History Bulletin of the Siam Society* **35**, 47–56.

Proctor, K., Petrie, B., Barden, R., Arnot, T. & Kasprzyk-Hordern, B. (2019). Multi-residue ultra-performance liquid chromatography coupled with tandem mass spectrometry method for comprehensive multi-class anthropogenic compounds of emerging concern analysis in a catchment-based exposure-driven study. *Analytical and Bioanalytical Chemistry* **411**, 7061–7086.

Ramage, S., Camacho-Muñoz, D. & Petrie, B. (2019). Enantioselective LC-MS/MS for anthropogenic markers of septic tank discharge. *Chemosphere* **219**, 191–201.

Raven, P.J. & George, J.J. (1989). Recovery by riffle macroinvertebrates in a river after a major accidental spillage of chlorpyrifos. *Environmental Pollution* **59**, 55–70.

Roberts, P.H. & Thomas, K. V. (2006). The occurrence of selected pharmaceuticals in wastewater effluent and surface waters of the lower Tyne catchment. *Science of The Total Environment* **356**, 143–153.

Rothwell, J.J., Dise, N.B., Taylor, K.G., Allott, T.E.H., Scholefield, P., Davies, H. & Neal, C. (2010). A spatial and seasonal assessment of river water chemistry across North West England. *Science of The Total Environment* **408**, 841–855.

RTT (2004). UK TECHNICAL ADVISORY GROUP ON THE WATER Type Specific Reference Condition Descriptions for Rivers in Great Britain FRAMEWORK DIRECTIVE Type Specific Reference Condition Descriptions for Rivers in Great Britain. *RTT*, 1-21.

Ryan, A.C., Tomasso, J.R. & Klaine, S.J. (2009). Influence of pH, Hardness, Dissolved Organic Carbon Concentration, and Dissolved Organic Matter Source on the Acute Toxicity of Copper to *Daphnia Magna* in Soft Waters: Implications for the Biotic Ligand Model. *Environmental Toxicology and Chemistry* **28**, 1663–1670.

Sankaramanachi, S.K. & Qasim, S.R. (1999). Metal toxicity evaluation using bioassay and microtox^TM^. *International Journal of Environmental Studies* **56**, 187–199.

Shepherd, K.A., Ellis, P.A. & Rivett, M.O. (2006). Integrated understanding of urban land, groundwater, baseflow and surface-water quality—The City of Birmingham, UK. *Science of The Total Environment* **360**, 180–195.

Sims, N., Holton, E., Archer, E., Botes, M., Wolfaardt, G. & Kasprzyk-Hordern, B. (2023). In-situ multi-mode extraction (iMME) sampler for a wide-scope analysis of chemical and biological targets in water in urbanized and remote (off-the-grid) locations. *Science of The Total Environment* **859**, 160034.

Spehar, R.L. & Fiandt, J.T. (1986). Acute and Chronic Effects of Water Quality Criteria-Based Metal Mixtures on Three Aquatic Species. *Environmental Toxicoloy and chemistry* **5**, 917–931.

Spurgeon, D., Wilkinson, H., Civil, W., Hutt, L., Armenise, E., Kieboom, N., Sims, K. & Besien, T. (2021). Worst-case ranking of organic substances detected in groundwater and surface waters in England. *Science of The Total Environment* **835**, 1–96.

Taylor, P.A., A.J. Stewart & Holt, L. (1988) Toxicity of common salts to three biotoxicity test organisms. *Engineers National Meeting*, 1.

Tišler, T. & Zagorc-Končan, J. (2002). Acute and chronic toxicity of arsenic to some aquatic organisms. *Bulletin of Environmental Contamination and Toxicology* **69**, 421–429.

Tsui, M.T.K. & Wang, W.X. (2005). Influences of maternal exposure on the tolerance and physiological performance of *Daphnia magna* under mercury stress. *Environmental Toxicology and Chemistry* **24**, 1228–1234.

Tsui, M.T.K., Wang, W.X. & Chu, L.M. (2005). Influence of glyphosate and its formulation (Roundup®) on the toxicity and bioavailability of metals to *Ceriodaphnia dubia*. *Environmental Pollution* **138**, 59–68.

Valencia-Avellan, M., Slack, R., Stockdale, A., John, R. & Mortimer, G. (2017). Understanding the mobilisation of metal pollution associated with historical mining in a carboniferous upland catchment. *Environmental Science* **19**, 1061.

Vareille-Morel, C. (1982). Natural tolerance and acclimation of different populations of *Austropotamobius pallipes* (Le.) to heavy metals (chromium and lead). *Acta Oecologica* **3**, 105–122.

Vellinger, C., Gismondi, E., Felten, V., Rousselle, P., Mehennaoui, K., Parant, M. & Usseglio-Polatera, P. (2013). Single and combined effects of cadmium and arsenate in *Gammarus pulex* (Crustacea, Amphipoda): Understanding the links between physiological and behavioural responses. *Aquatic Toxicology* **140**–**141**, 106–116.

Wilkinson, J.L., Boxall, A.B.A. & Kolpin, D.W. (2019). A novel method to characterise levels of pharmaceutical pollution in large-scale aquatic monitoring campaigns. *Applied Sciences* **9**, 1368.

Wollenberger, L., Halling-Sørensen, B. & Kusk, K.O. (2000). Acute and chronic toxicity of veterinary antibiotics to Daphnia magna. *Chemosphere* **40**, 723–730.

Yoshimura, H. & Endoh, Y.S. (2005). Acute toxicity to freshwater organisms of antiparasitic drugs for veterinary use. *Environmental Toxicology* **20**, 60–66.

Zhang, Y., Guo, P., Wang, M., Wu, Y., Sun, Y., Su, H. & Deng, J. (2021). Mixture toxicity effects of chloramphenicol, thiamphenicol, florfenicol in *Daphnia magna* under different temperatures. *Ecotoxicology* **30**, 31–42.

Zhang, Z.L. & Zhou, J.L. (2007). Simultaneous determination of various pharmaceutical compounds in water by solid-phase extraction–liquid chromatography–tandem mass spectrometry. *Journal of Chromatography A* **1154**, 205–213.

Zhou, J. & Broodbank, N. (2014). Sediment-water interactions of pharmaceutical residues in the river environment. *Water Research* **48**, 61–70.
